# Anti-interleukin-1 treatment in patients with rheumatoid arthritis and type 2 diabetes (TRACK): A multicentre, open-label, randomised controlled trial

**DOI:** 10.1371/journal.pmed.1002901

**Published:** 2019-09-12

**Authors:** Piero Ruscitti, Francesco Masedu, Saverio Alvaro, Paolo Airò, Norma Battafarano, Luca Cantarini, Francesco Paolo Cantatore, Giorgio Carlino, Virginia D'Abrosca, Micol Frassi, Bruno Frediani, Daniela Iacono, Vasiliki Liakouli, Roberta Maggio, Rita Mulè, Ilenia Pantano, Immacolata Prevete, Luigi Sinigaglia, Marco Valenti, Ombretta Viapiana, Paola Cipriani, Roberto Giacomelli

**Affiliations:** 1 Division of Rheumatology, Department of Biotechnological and Applied Clinical Sciences, University of L'Aquila, L'Aquila, Italy; 2 Division of Medical Statistics, Department of Biotechnological and Applied Clinical Science, University of L'Aquila, L'Aquila, Italy; 3 Rheumatology and Clinical Immunology Unit, Department of Clinical and Experimental Sciences, University of Brescia, Brescia, Italy; 4 Department of Rheumatology, Gaetano Pini Institute, Milan, Italy; 5 Research Center of Systemic Autoinflammatory Diseases and Behçet's Disease and Rheumatology-Ophthalmology Collaborative Uveitis Center, Department of Medical Sciences, Surgery and Neurosciences, University of Siena, Siena, Italy; 6 Rheumatology Clinic, Department of Medical and Surgical Sciences, University of Foggia Medical School, Foggia, Italy; 7 Rheumatology Service, ASL Lecce—DSS Casarano and Gallipoli (LE), Casarano (LE), Italy; 8 Division of Rheumatology, Department of Precision Medicine, University of Campania ‘Luigi Vanvitelli’, Naples, Italy; 9 Rheumatology Unit, S.Orsola-Malpighi Teaching Hospital, Bologna, Italy; 10 Rheumatology Unit, Azienda Ospedaliera San Camillo-Forlanini, Rome, Italy; 11 Rheumatology Unit, Department of Medicine, University of Verona, Verona, Italy; Harvard Medical School, UNITED STATES

## Abstract

**Background:**

The inflammatory contribution to type 2 diabetes (T2D) has suggested new therapeutic targets using biologic drugs designed for rheumatoid arthritis (RA). On this basis, we aimed at investigating whether interleukin-1 (IL-1) inhibition with anakinra, a recombinant human IL-1 receptor antagonist, could improve both glycaemic and inflammatory parameters in participants with RA and T2D compared with tumour necrosis factor (TNF) inhibitors (TNFis).

**Methods and findings:**

This study, designed as a multicentre, open-label, randomised controlled trial, enrolled participants, followed up for 6 months, with RA and T2D in 12 Italian rheumatologic units between 2013 and 2016. Participants were randomised to anakinra or to a TNFi (i.e., adalimumab, certolizumab pegol, etanercept, infliximab, or golimumab), and the primary end point was the change in percentage of glycated haemoglobin (HbA1c%) (EudraCT: 2012-005370-62 ClinicalTrial.gov: NCT02236481).

In total, 41 participants with RA and T2D were randomised, and 39 eligible participants were treated (age 62.72 ± 9.97 years, 74.4% female sex). The majority of participants had seropositive RA disease (rheumatoid factor and/or anticyclic citrullinated peptide antibody [ACPA] 70.2%) with active disease (Disease Activity Score-28 [DAS28]: 5.54 ± 1.03; C-reactive protein 11.84 ± 9.67 mg/L, respectively). All participants had T2D (HbA1c%: 7.77 ± 0.70, fasting plasma glucose: 139.13 ± 42.17 mg). When all the enrolled participants reached 6 months of follow-up, the important crude difference in the main end point, confirmed by an unplanned ad interim analysis showing the significant effects of anakinra, which were not observed in the other group, led to the study being stopped for early benefit. Participants in the anakinra group had a significant reduction of HbA1c%, in an unadjusted linear mixed model, after 3 months (β: −0.85, *p* < 0.001, 95% CI −1.28 to −0.42) and 6 months (β: −1.05, *p* < 0.001, 95% CI −1.50 to −0.59). Similar results were observed adjusting the model for relevant RA and T2D clinical confounders (male sex, age, ACPA positivity, use of corticosteroids, RA duration, T2D duration, use of oral antidiabetic drug, body mass index [BMI]) after 3 months (β: −1.04, *p* < 0.001, 95% CI −1.52 to −0.55) and 6 months (β: −1.24, *p* < 0.001, 95% CI −1.75 to −0.72). Participants in the TNFi group had a nonsignificant slight decrease of HbA1c%. Assuming the success threshold to be HbA1c% ≤ 7, we considered an absolute risk reduction (ARR) = 0.42 (experimental event rate = 0.54, control event rate = 0.12); thus, we estimated, rounding up, a number needed to treat (NNT) = 3. Concerning RA, a progressive reduction of disease activity was observed in both groups. No severe adverse events, hypoglycaemic episodes, or deaths were observed. Urticarial lesions at the injection site led to discontinuation in 4 (18%) anakinra-treated participants. Additionally, we observed nonsevere infections, including influenza, nasopharyngitis, upper respiratory tract infection, urinary tract infection, and diarrhoea in both groups. Our study has some limitations, including open-label design and previously unplanned ad interim analysis, small size, lack of some laboratory evaluations, and ongoing use of other drugs.

**Conclusions:**

In this study, we observed an apparent benefit of IL-1 inhibition in participants with RA and T2D, reaching the therapeutic targets of both diseases. Our results suggest the concept that IL-1 inhibition may be considered a targeted treatment for RA and T2D.

**Trial registration:**

The trial is registered with EU Clinical Trials Register, EudraCT Number: 2012-005370-62 and with ClinicalTrial.gov, number NCT02236481.

## Abstract 2

Roberto Giacomelli and co-authors study treatment of comorbid rheumatoid arthritis and type 2 diabetes via interleukin-1 blockade.

## Author summary

**Why was this study done?:**

- A growing body of evidence suggests the inflammatory contribution to type 2 diabetes (T2D) as observed in rheumatoid arthritis (RA).
- Interleukin-1 (IL-1) would be a common pathogenic mediator in T2D and RA, suggesting a possible common therapeutic target.
- We investigated whether IL-1 inhibition with anakinra, a human IL-1-receptor antagonist, could improve both glycaemic and inflammatory parameters in participants with RA and T2D compared with tumour necrosis factor (TNF) inhibitors (TNFis).

**What did the researchers do and find?:**

- In a multicentre, open-label, randomised controlled trial, 39 participants with RA and T2D (age 62.72 ± 9.97 years, 74.4% female sex) were randomised to anakinra or to TNFi in order to evaluate the efficacy of these drugs in controlling the metabolic alterations of T2D.
- Anakinra showed a significant improvement of metabolic alteration (reduction of percentage of glycated haemoglobin [HbA1c%]) after both 3 months and 6 months of therapy (crude difference of 0.93 HbA1c% between groups), whereas TNFi did not show any significant improvement on these features.
- No severe adverse events, hypoglycaemic episodes, or deaths were observed.

**What do these findings mean?:**

- Our results suggest the concept that IL-1 inhibition may be considered a targeted treatment for RA and T2D.
- Managing the inflammatory disease and the metabolic comorbidity by an agent inhibiting IL-1 may lead to a consequent beneficial impact on participants’ compliance, their overall cardiovascular (CV) risk, and the burden of healthcare costs.
- Our study has some limitations, mainly due to open-label design, and future studies are necessary to fully clarify this topic.

## Introduction

The management of rheumatoid arthritis (RA) has been significantly improved over the last 2 decades by the introduction of biologic disease-modifying antirheumatic drugs (bDMARDs) associated with the treat-to-target approach [1]. However, despite the significant reduction of the joint structural damages, several studies have shown that cardiovascular (CV) events are emerging as the leading cause of death in these patients, thus pointing out the close association between RA and CV disease (CVD) [2]. In fact, the ‘traditional’ CV risk factors and the systemic proinflammatory process during RA may synergise the enhancement of CVD burden in these patients [2,3]. As far as the role of traditional CV risk factors in RA is concerned, an increased association has been highlighted between RA and aberrant glucose metabolism, explaining the elevated prevalence of type 2 diabetes (T2D) and insulin resistance (IR) in these patients [3]. Remarkably, interleukin-1β (IL-1β), IL-6, and tumour necrosis factor (TNF), which are involved in the pathogenesis of RA, may also play a pivotal role in the development of IR [4]. Interestingly, the increased glucose levels stress the pancreatic islets and insulin-sensitive tissues, leading to hyperproduction of IL-β via nucleotide-binding oligomerization domain-like receptors-, leucine-rich repeat-, and pyrin domain–containing 3 (NLRP3) inflammasome [4,5]. This overexpressed IL-1β contributes to pathogenesis of T2D, leading to both dysfunction and apoptosis of β-cells, with consequent decreased insulin production [5]. Furthermore, IL-1β could directly inhibit glucose-stimulated insulin secretion and trigger the intrinsic mitochondrial apoptotic pathway in β-cells [5]. The recent knowledge of the contribution of inflammatory processes to the pathogenesis of T2D has suggested new antidiabetic therapeutic strategies in which bDMARDs, which are commonly used in the treatment of RA, may be effective in improving glucose abnormalities [5]. However, despite the growing body of evidence from preclinical and clinical studies confirming the role of targeting inflammatory cytokines in improving clinical and laboratory outcomes in T2D patients [5], no clinical trial specifically designed to evaluate the glycaemic outcome in patients with RA and T2D has been planned, so far.

RA and T2D share the treat-to-target approach, in which an intensive pharmacological strategy is devoted to achieving the predetermined therapeutic goal, which has been shown to be associated with better long-term outcome and decreased mortality. On this basis, a single treatment controlling both these diseases seems to be a promising choice to improve the management of those patients with RA and T2D [6]. In fact, any multidrug approach is frequently associated with decreased patient compliance; usually, the number of medications prescribed is inversely proportional to the adherence to therapies [7], and any single therapeutic strategy concurrently treating 2 different diseases may also help the deciders of health policies to optimise the social costs while maintaining the quality of treatments. Finally, although these comorbidities are frequently observed in patients with RA [2,3], the evidence deriving from randomised clinical trials designed to evaluate the safety and efficacy of any drug, using strict enrolment criteria, not mirroring real life, cannot fully elucidate the effect on comorbidities that are generally included in the exclusion criteria, thus decreasing the generalisability of the results [8]. To overcome these limitations, in a specifically designed study, we aimed to investigate whether IL-1 inhibition could induce the improvement of both glycaemic and inflammatory parameters in participants with RA and T2D, when compared with participants treated with a TNF inhibitor (TNFi), in order to improve the management of these participants in a multicentre, randomised, open-label, prospective, controlled, parallel-group trial.

## Methods

### Study design

The Treatment of Rheumatoid Arthritis and Comorbidities with Kineret (anakinra) (TRACK) study was designed as a multicentre, randomised, open-label, prospective, controlled, parallel-group study to investigate whether IL-1 inhibition could induce improvement in both metabolic and inflammatory parameters in participants with RA and T2D when compared with participants treated with TNFis (S1 Fig). This study was designed as a nonprofit study, according to Italian law ‘Decreto Ministero della Salute 17 Dicembre 2004’, to support independent research in Italy. The study protocol is available in S1 Text and S2 Text.

**Fig 1:**
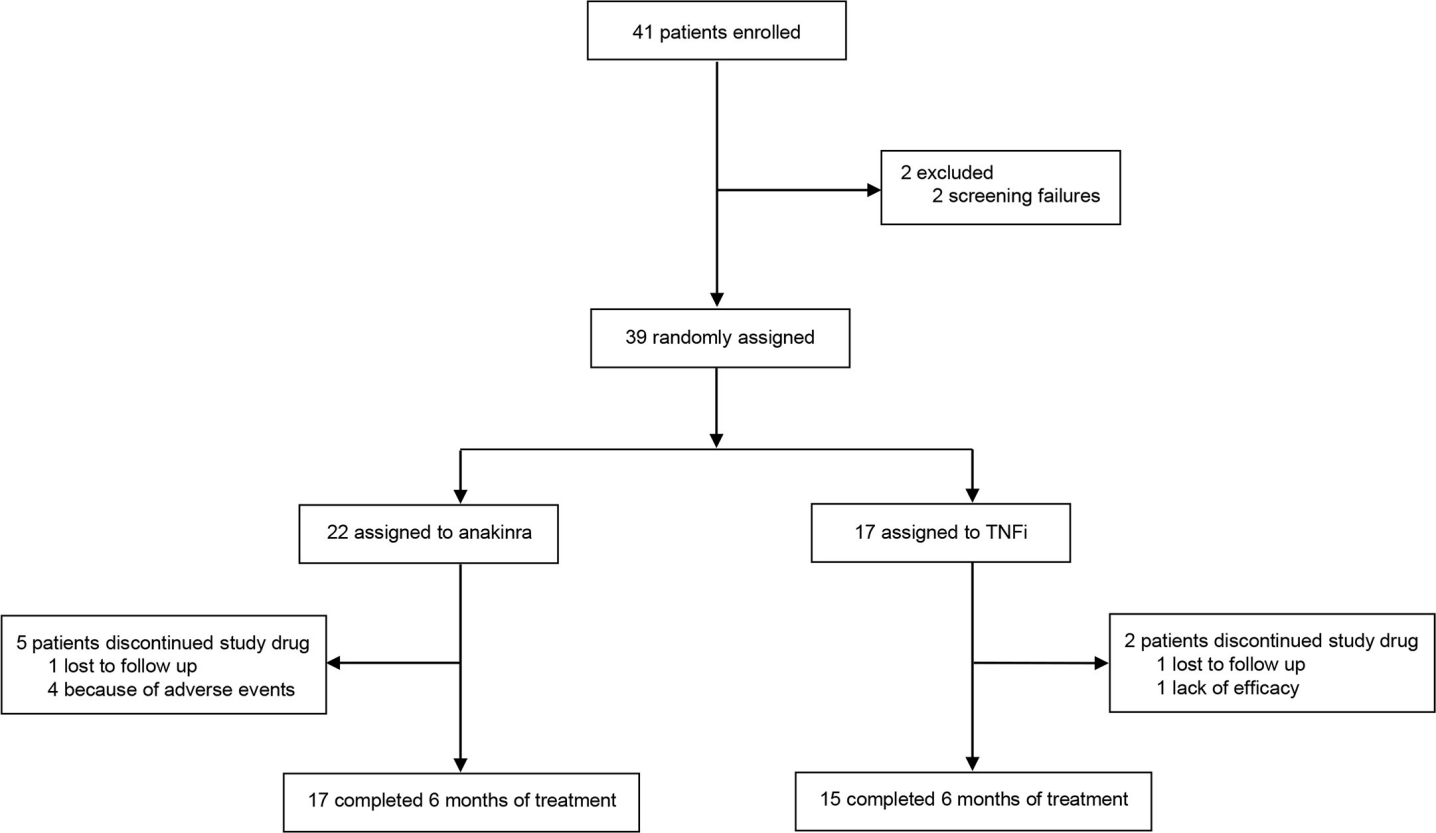
Trial profile. Participants were recruited from June 2013 to March 2016 and were randomised to either once-daily recombinant human interleukin-1-receptor antagonist (100 mg of anakinra) by daily subcutaneous self-administration or TNFi administered according to relevant data sheets. TNFi, tumour necrosis factor inhibitor.

We enrolled 41 participants, who were recruited from June 2013 to March 2016, in 12 Italian rheumatologic units (Fig 1). Out of 41 participants, 39 were randomised in the 2 arms, the first arm receiving anakinra, a human IL-1-receptor antagonist, and the other arm receiving TNFis. Participants continued their baseline antidiabetic therapy, their own dietary and lifestyle habits, and their baseline RA therapy.

The study protocol was reviewed and approved by the local ethics committee (Comitato Etico ASL1 Avezzano-Sulmona-L’Aquila, L’Aquila, Italy; protocol number 0020902/13), and the study was performed according to the Good Clinical Practice guidelines and the latest Declaration of Helsinki. Written informed consent was obtained from all participants before randomisation and any study-related procedure. Our study was monitored by an external agency. This agency monitored all the procedures of the study. Data monitoring was undertaken by the lead investigators and this agency.

This study is reported per the Consolidated Standards of Reporting Trials (CONSORT) guideline (S1 CONSORT Checklist).

When all the enrolled participants reached 6 months of follow-up, the important crude difference in the main end point, confirmed by ad interim analysis showing the significant effects of anakinra, which were not observed in the other group, induced stoppage of the study for early benefit (see below, detailed in Sample size).

### Participants

Eligible participants were as follows: male and female participants aged ≥18 years; affected by RA, classified according to American College of Rheumatology/European League Against Rheumatism (ACR/EULAR) criteria; with moderate to severe RA, with an inadequate response to previous treatment with methotrexate (MTX) characterised by a Disease Activity Score-28 (DAS28) > 3.2; affected by T2D, classified according to American Diabetes Association (ADA) criteria and of at least 6 months’ duration; and with percentage of glycated haemoglobin (HbA1c%) > 7% and < 10% and a body mass index (BMI) < 35. For participants previously treated with a bDMARD, an appropriate washout period, according to the relevant data sheets, was mandatory; stable corticosteroids (CCSs) therapy at the lowest effective dosage and, furthermore, not more than 7.5 mg daily of prednisone equivalent were allowed [9]. Participants treated with antirheumatic drugs at baseline could not change drug administration and dosage regimen for at least 1 month before enrolment. Similarly, participants treated with antidiabetic drugs at baseline could not change drug administration and dosage regimen for at least 3 months before enrolment.

Exclusion criteria were as follows: T2D diagnosed more than 10 years prior to the study; ongoing acute or chronic infection; increased (>30 mg/L) levels of C-reactive protein (CRP); fever; ongoing antibiotic therapy; chronic granulomatous infections, such as tuberculosis; history of recurrent infections; fasting C-peptide values < 0.5 ng/mL (0.1665 nmol/L); presence of neutropenia (white blood count < 2,000/mm^3^) or anaemia (haemoglobin < 11 g/dL for men and 10 g/dL for women); presence of one or more contraindications reported in the data sheet of anakinra or TNFi; presence of one or more contraindications to MTX; previous ischaemic attack or myocardial infarction; heart failure of New York Heart Association (NYHA) class III or IV; hepatic or progressive liver disease (values of alanine aminotransferase/aspartate aminotransferase [ALAT/ASAT] elevated by at least 2-fold compared with normal values); pregnancy, or women not using contraceptive measures; breastfeeding; participation in another clinical study up to 6 months before randomisation; depressive syndrome or other serious psychiatric illness; presence of known malignancy; clinically significant history of alcohol abuse or drug addiction; any condition that, in the opinion of the investigator, could preclude the possibility of use of study drugs in compliance with data sheet indications; and any other condition or laboratory parameter that, in the opinion of the investigator, could preclude the participation of the subject in the study. The exclusion criteria mainly derived from the data sheets of the administered medications.

### Randomisation and masking

The research was designed as a multicentre, randomised, open-label, prospective, controlled, parallel-group study. Participants were assigned to each treatment group using the method of randomised block randomisation to control for possible unbalancing excess between trial arms. The randomisation list was generated by the coordinating centre. The study protocol was open label (not masked) for all participants, physicians, and statisticians. However, the clinical activities were open label to both participants and physicians, whereas the statisticians performed an external blind analysis of the deidentified collected data.

### Interventions

After the screening phase, participants were included in the study and randomised to receive either anakinra (100 mg) by daily subcutaneous self-administration or TNFi administered in accordance with corresponding data sheets. Follow-up visits were scheduled at baseline, 3 months, 6 months, 12 months, and 24 months. The study underwent an early stoppage, when all the enrolled participants reached a follow-up of 6 months, and consequently, we analysed data only after 3 and 6 months. During each scheduled visit, participants were assessed by complete medical history, and RA and T2D features were recorded; the study was associated with an extensive activity in monitoring drugs compliance. We developed a specific form to be filled by the participants and to be checked by investigators during each scheduled visit. It was not possible to increase the antidiabetic and the antirheumatic therapy to reduce possible biases in the analysis of end points. At the same time, the possible reduction of the therapy was allowed to limit the participants’ exposure to unnecessary risk of side effects. This was mainly related to the issue of hypoglycaemia during T2D. The study was associated with extensive activity in monitoring also diet habit and lifestyle, and BMI was used as a proxy of these features.

### Outcomes

The predefined primary end point was the change in HbA1c% levels between baseline and subsequent visits. After the early stoppage of the trial, we analysed the primary end point after 3 and 6 months of follow-up. According to available literature, an absolute difference of 0.25% was considered to be clinically significant between the 2 arms [10]. A number of secondary end points were planned to evaluate the effect of study drugs on RA signs and symptoms and additional metabolic parameters. DAS28, simplified disease activity index (SDAI), percentage of participants reaching good EULAR clinical response and remission, physician global assessment (PGA), visual analogue scale (VAS) of pain, erythrocyte sedimentation rate (ESR), and CRP were investigated in assessing RA features. Fasting plasma glucose (FPG), microalbuminuria, the presence of retinopathy, and BMI were investigated in assessing T2D parameters.

Safety of the treatment was evaluated during all scheduled visits, and any suspected adverse event (AE) was recorded and coded according to the Medical Dictionary for Regulatory Activities (MedDRA) system organ class (SOC) classification. Compliance with study drugs was assessed by checking the patient’s diary, in which participants were asked to record each administration of medications.

### Sample size

The study was designed to demonstrate the superiority of anakinra compared with treatment with a TNFi drug in terms of efficacy in improving glycaemic control as well as in reducing the signs and symptoms of RA and T2D. The sample size, including both arms of the trial, was 168 participants, assuming a power of 90% and an α of 5%, considering the HbA1c% difference expected between treatments of 0.25% [10], with a standard deviation of 0.5. Considering a 10% withdrawal rate as well as an inflation factor of 1.01 deriving from the use of O'Brien-Fleming's stopping boundaries [11], resulting from an ad interim analysis scheduled after 12 months from the beginning of the recruitment, the total sample had to be at least 200. The sample size was deliberately rounded up to 100 participants for each arm. After 12 months from the study onset (timing for the planned ad interim analysis), we recruited 10 participants who underwent anakinra and 9 participants who underwent TNFi (all participants were followed at least for 3 months). These samples and the recruitment rate raised issues of achievable statistical power and concerns about study feasibility. In fact, assuming a very high Cohen size effect (0.9), given a significance level of 0.05, with arms having 10 and 9 participants, respectively, the study power was lowered from the planned 90% to the low power of 45%. However, from a clinical perspective, we observed a 0.71 crude clinical difference in HbA1c% between anakinra (6.96) and TNFi (7.67) after 3 months, suggesting a plausible relevant difference, which deserved to be better assessed. When all the enrolled participants reached 6 months of follow-up (20% of the planned sample size), we performed an unplanned ad interim analysis, aimed to achieve at least a statistical power of 80%, assumed as the lowest acceptable level. Given these premises, we stated that assuming the previous very high Cohen size effect (0.9), given a significance level of 0.05, the power requested is coherent with arms containing about 20% of the planned study sample size. Because of the large and relevant clinical results concerning the difference in HbA1c%, we assessed our data in order to achieve at least a power of 80% (the lowest acceptable power) but considering a high (disadvantageous) effect size to be reached. The accumulating data were evaluated by an unplanned ad interim analysis, mirroring a continuous sequential design model. This ad hoc approach has been frequently adopted by a number of statistical reviewers faced with problems of unplanned ad interim analyses during the review process of clinical trials [12]. The significant effect of anakinra in decreasing HbA1c%, showed by the ad interim analysis, which was not observed in the other group (crude difference of 0.93 HbA1c% between groups), induced an early stoppage of the study for early benefit [13,14]. Furthermore, we checked the fulfilment of the early stoppage by using the O'Brien-Fleming boundaries. The O'Brien-Fleming boundaries could be calculated planning 3 interim analyses or 2 interim analyses, not considering the first sample and clinical appraisal we performed (10 participants versus 9 participants). The latter, despite being less conceptually appropriate, nonetheless is statistically more demanding in terms of the O'Brien-Fleming boundary and its corresponding *p*-value. In this case, we considered as appropriate the more demanding O'Brien-Fleming boundary, which is 2.782 (*p* = 0.0054) and could be compared with our study t statistics of 3.959 (*p* < 0.001). Finally, we reassessed the results concerning the main end point considering a higher threshold of statistical significance (*p* < 0.001 instead of *p* < 0.05).

### Statistical analysis

The statistical analysis provided descriptive statistics, graphical data inspection, and linear mixed models for the primary end point (HbA1c% response during the follow-up) as well as for main secondary end points (FPG and DAS28 analysed as score), setting the type I error at 5%. For descriptive purpose, *t* tests with explorative intent without accounting for the longitudinal design were performed in analysing primary and secondary end points. Similarly, the proportion of participants reaching a good response according to EULAR (DAS28 analysed as categories) as well as other analyses of proportions were managed with Fisher’s exact test. Concerning the main end points (HbA1c%, FPG, and DAS28 analysed as score), linear mixed models were set up as random intercept and random slope models, assuming an unstructured covariance matrix. The interaction between time and treatment was the major issue in the identification of models. In addition to male sex and age, some clinical variables were added in the model of the primary end point concerning HbA1c% (male sex, age, anticyclic citrullinated peptide antibody [ACPA] positivity, use of CCSs, RA duration, T2D duration, use of oral antidiabetic drug, BMI) because of clinical relevance of these features. Fixed and random effects were estimated using the maximum-likelihood method, using an unstructured covariance matrix to account for the longitudinal design. Overall model fitting was assessed by calculating log-likelihood statistics. Furthermore, assuming the success threshold to be HbA1c% ≤ 7, number needed to treat (NNT) was estimated. The noncompliance with the treatment assigned by randomisation was treated using an intent-to-treat analytic model. Statistics and model parameters were calculated using the statistical STATA software, version 14 (StataCorp, College Station, TX, United States).

## Results

### Baseline characteristics

The current analysis included 39 of the 41 participants with RA who were enrolled and randomised to receive either anakinra or TNFis, from June 2013 to March 2016, as detailed in Fig 1. Two participants were classified as screening failures, not fulfilling inclusion/exclusion criteria. The majority of participants had a seropositive RA disease (70.2%). All participants had an active disease (DAS28: 5.54 ± 1.03; ESR: 32.79 ± 18.78 mm per hour; CRP 11.84 ± 9.67 mg/L, respectively). All participants were treated with MTX, 10.3% of participants received combination therapy with MTX and hydroxychloroquine (HCQ), and 7.7% of participants received combination therapy with MTX and sulfasalazine (SSZ). Sixty-six percent of the enrolled participants were treated with CCSs, at the lowest effective dosage, and not more than 7.5 mg daily of prednisone equivalent. All participants had T2D (HbA1c%: 7.77 ± 0.70, FPG: 139.13 ± 42.17 mg; microalbuminuria 10.88 ± 9.33 mg/L, respectively). According to the inclusion criterion stating a disease duration of <10 years, all patients with T2D had acceptable disease duration (median 1 year [25%: 0.6; 75%: 2]). All participants received antidiabetic medications: 74.4% were treated with antidiabetic oral drugs (mainly metformin), and 25.6% were treated with insulin. Furthermore, 68.2% of participants were affected by high blood pressure, 25.6% of participants by osteoporosis, 20.5% by dyslipidaemia, 20.5% by thyroidopathies, and 5.1% by atrial fibrillation. The baseline characteristics of 22 participants receiving anakinra (anakinra group) and the 17 participants receiving TNFi (TNFi group) are summarised in Table 1. During the follow-up, we lost to follow-up 2 participants, who missed the scheduled visits and did not come back to the centres. When all the enrolled participants reached 6 months of follow-up, the important crude difference in the main end point (crude difference of 0.93 HbA1c% between groups), confirmed by ad interim analysis showing the significant effects of anakinra, which were not observed in the other group, led to an early stoppage of the study.

**Table 1:** Baseline clinical characteristics of the randomised participants.

Baseline clinical characteristics	Enrolled participants (*n* = 39)	Anakinra (*n* = 22)	TNFi (*n* = 17)
Age, mean ± SD (years)	62.72 ± 9.97	62.86 ± 9.70	62.53 ± 10.60
Female, *n* (%)	29 (74.4%)	17 (77.2%)	12 (70.6%)
RA clinical characteristics			
RF, *n* (%)	22 (56.4%)	12 (54.5%)	10 (45.4%)
ACPA, *n* (%)	24 (61.5%)	14 (63.6%)	10 (58.8%)
RA duration (years), median (25%; 75%)	2 (0.6; 5)	2 (0.8; 5)	1 (0.6; 5)
DAS28, mean ± SD	5.54 ± 1.03	5.43 ± 1.18	5.70 ± 0.80
SDAI, mean ± SD	35.38 ± 22.66	34.98 ± 25.17	35.86 ± 19.68
Physician global assessment, mean ± SD	62.00 ± 19.28	62.00 ± 19.81	62.00 ± 19.17
Patient global assessment, mean ± SD	66.51 ± 20.99	63.95 ± 24.35	69.82 ± 15.73
VAS, mean ± SD	67.77 ± 26.47	66.86 ± 29.46	68.94 ± 22.86
ESR (mm/h), mean ± SD	32.79 ± 18.78	35.55 ± 19.13	29.24 ± 18.26
CRP (mg/L), mean ± SD	11.84 ± 9.67	12.66 ± 10.14	10.78 ± 9.23
CCSs, *n* (%)	26 (66.7%)	13 (59.1%)	13 (76.5%)
MTX, *n* (%)	39 (100%)	22 (100%)	17 (100%)
HCQ, *n* (%)	4 (10.3%)	2 (9.1%)	2 (11.8%)
SSZ, *n* (%)	3 (7.7%)	2 (9.1%)	1 (5.9%)
Anakinra, *n* (%)		22 (100%)	
TNFi, *n* (%)			17 (100%)
ADA, *n* (%)			7 (41.2%)
CZP, *n* (%)			3 (17.6%)
ETN, *n* (%)			3 (17.6%)
IFX, *n* (%)			2 (11.8%)
GOL, *n* (%)			2 (11.8%)
T2D clinical characteristics			
T2D duration (years), median (25%; 75%)	1 (0.6; 2)	0.7 (0.6; 1)	2 (0.8; 3)
C peptide (mg/dL), mean ± SD	2.66 ± 1.34	2.92 ± 1.42	2.32 ± 1.19
HbA1c (%), mean ± SD	7.77 ± 0.70	7.73 ± 0.67	7.83 ± 0.76
FPG, mean ± SD	139.13 ± 42.17	139.05 ± 50.09	139.25 ± 29.55
Microalbuminuria (mg/L), mean ± SD	10.88 ± 9.33	12.14 ± 11.93	8.15 ± 5.98
Diabetic retinopathy, *n* (%)	5 (12.8%)	3 (13.6%)	2 (11.8%)
BMI, mean ± SD	27.93 ± 4.04	27.59 ± 4.49	28.37 ± 3.47
Total cholesterol (mg/dL), mean ± SD	190.52 ± 69.32	182.56 ± 65.12	195.52 ± 59.35
Triglycerides (mg/dl), mean ± SD	122.85 ± 54.59	123.78 ± 45.12	126.52 ± 49.09
Oral antidiabetic drugs, *n* (%)	29 (74.4%)	18 (81.8%)	11 (64.7%)
Insulin therapy, *n* (%)	10 (25.6%)	6 (27.3%)	4 (23.5%)
Statins, *n* (%)	5 (12.8%)	3 (13.6%)	2 (11.8%)
Comorbidities, *n* (%)	30 (76.9%)	18 (81.8%)	12 (70.6%)

Abbreviations: ACPA, anticyclic citrullinated peptide antibody; ADA, adalimumab; BMI, body mass index; CCS, corticosteroid; CRP, C-reactive protein; CZP, certolizumab pegol; DAS28, Disease Activity Score-28; ESR, erythrocyte sedimentation rate; ETN, etanercept; FPG, fasting plasma glucose; GOL, golimumab; HbA1c%, percentage of glycated haemoglobin; HCQ, hydroxychloroquine; IFX, infliximab; MTX, methotrexate; RA, rheumatoid arthritis; RF, rheumatoid factor; SDAI, simplified disease activity index; SSZ, sulfasalazine; T2D, type 2 diabetes; TNFi, tumour necrosis factor inhibitor; VAS, visual analogue scale

### The primary end point

**Fig 2:**
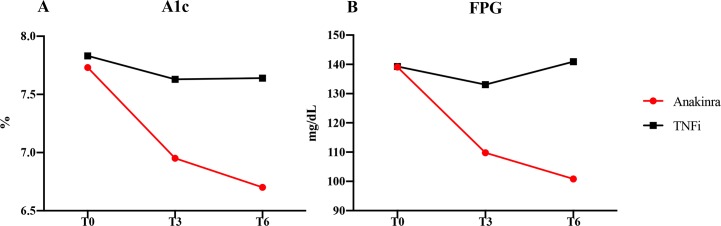
Measures of glycaemic control and bodyweight. Metabolic measures of glycaemic control at baseline (T0) and after 3 (T3) and 6 (T6) months of treatment with anakinra or TNFi. (A) HbA1c%. T0: anakinra group (7.73% ± 0.67) versus TNFi group (7.83% ± 0.76); T3: anakinra group (6.95% ± 0.61) versus TNFi group (7.63% ± 0.68), *p* = 0.0038; T6: anakinra group (6.70% ± 0.67) versus TNFi group (7.64% ± 0.65), *p* < 0.001. (B) FPG. T0: anakinra group (139.05 ± 50.09 mg/dL) versus TNFi group (139.25 ± 29.55 mg/dL); T3: anakinra group (109.78 ± 30.58 mg/dL) versus TNFi group (133.06 ± 27.72 mg/dL), *p* = 0.027; T6: anakinra group (100.81 ± 11.11 mg/dL) versus TNFi group (140.93 ± 39.45 mg/dL), *p* < 0.001. FPG, fasting plasma glucose; HbA1c%, percentage of glycated haemoglobin; TNFi, tumour necrosis factor inhibitor.

During the trial, we observed a progressive reduction of HbA1c% in anakinra-treated participants when compared with TNFi-treated participants, as shown in Fig 2.

In descriptive terms, at baseline, no difference was observed between the 2 arms when analysing HbA1c values (anakinra group: 7.73% [61 mmol/mol] ± 0.67 versus TNFi group: 7.83% [62 mmol/mol] ± 0.76). After 3 months of treatment, there was a significant reduction in HbA1c% in anakinra-treated participants when compared with TNFi-treated participants (anakinra group: 6.95% [51 mmol/mol] ± 0.61 versus TNFi group: 7.63% [60 mmol/mol] ± 0.68, *p* = 0.0038). A further significant reduction of HbA1c% was observed in anakinra-treated participants when compared with TNFi-treated participants after 6 months of treatment (anakinra group: 6.70% [50 mmol/mol] ± 0.67 versus TNFi group: 7.64% [60 mmol/mol] ± 0.65, *p* < 0.001). These findings are summarised in S1 Table.

We further analysed the effects of the study drugs on the reduction of HbA1c% using linear mixed models. First, we observed a significant effect of anakinra treatment on the overall reduction of HbA1c% (β: −0.51, *p* = 0.02, 95% CI −0.91 to −0.10). In a nonadjusted linear mixed model, we observed a significant effect of anakinra treatment on reduction of HbA1c% after 3 months (β: −0.85, *p* < 0.001, 95% CI −1.28 to −0.42) and 6 months (β: −1.05, *p* < 0.001, 95% CI −1.50 to −0.59), and similar results were observed adjusting the model for possible RA and T2D clinical confounders (male sex, age, ACPA positivity, use of CCSs, RA duration, T2D duration, use of oral antidiabetic drug, BMI) after 3 months (β: −1.04, *p* < 0.001, 95% CI −1.52 to −0.55) and 6 months (β: −1.24, *p* < 0.001, 95% CI −1.75 to −0.72). On the contrary, TNFi treatment did not show significant effects on the levels of HbA1c% in the same time period. These findings are summarised in Table 2. Assuming the success threshold to be HbA1c% ≤ 7, we considered an absolute risk reduction (ARR) = 0.42 (experimental event rate [EER] = 0.54, control event rate [CER] = 0.12) so that we estimated, rounding up, a number needed to treat (NNT) = 3.

**Table 2:** Analysis of HbA1c% as clinical response, linear mixed models analysing the effect of study drugs on overall clinical response and adjusted for participants’ characteristics.

HbA1c%	β	SE	*p*	95% CI
Linear mixed model analysing the effect of study drug on overall clinical response				
Anakinra overall effect	−0.51	0.21	**0.02**	−0.91 to −0.10
Linear mixed model unadjusted for participants’ characteristics analysing the effect of study drug				
TNFi (3 months)	−0.16	0.11	0.13	−0.39 to 0.05
TNFi (6 months)	−0.06	0.14	0.66	−0.34 to 0.22
Anakinra (3 months)	−0.85	0.22	**<0.001**	−1.28 to −0.42
Anakinra (6 months)	−1.05	0.23	**<0.001**	−1.50 to −0.59
Linear mixed model adjusted for participants’ characteristics analysing the effect of study drug				
TNFi (3 months)	−0.17	0.11	0.13	−0.39 to 0.05
TNFi (6 months)	−0.06	0.14	0.68	−0.34 to 0.22
Anakinra (3 months)	−1.04	0.25	**<0.001**	−1.52 to −0.55
Anakinra (6 months)	−1.24	0.26	**<0.001**	−1.75 to −0.72
Male sex	0.07	0.25	0.77	−0.42 to 0.56
Age	−0.01	0.01	0.24	−0.03 to 0.01
RA duration (years)	0.10	0.04	0.06	0.02 to 0.17
ACPA	0.19	0.20	0.34	−0.20 to 0.58
CCSs	−0.29	0.23	0.20	−0.75 to 0.16
T2D duration (years)	−0.11	0.06	0.08	−0.24 to 0.01
Oral antidiabetic drug	−0.41	0.22	0.06	−0.84 to 0.02
BMI	−0.01	0.03	0.69	−0.07 to 0.04

Statistical significance was expressed by a *p*-value < 0.05. Bolded values indicate statistically significant results.

Abbreviations: ACPA, anticyclic citrullinated peptide antibody; BMI, body mass index; CCS, corticosteroids; HbA1c%, percentage of glycated haemoglobin; RA, rheumatoid arthritis; T2D, type 2 diabetes; TNFi, tumour necrosis factor inhibitor

### Metabolic secondary end points

Paralleling HbA1c%, there was a progressive reduction of FPG in anakinra-treated participants when compared with TNFi-treated participants, as shown in Fig 2. At baseline, no difference was observed between the arms in FPG values (anakinra group: 139.05 ± 50.09 mg/dL versus TNFi group: 139.25 ± 29.55 mg/dL). A significant reduction in FPG was observed in anakinra-treated participants when compared with TNFi-treated participants after 3 months of treatment (anakinra group: 109.78 ± 30.58 mg/dL versus TNFi group: 133.06 ± 27.72 mg/dL, *p* = 0.027) and after 6 months of treatment (anakinra group: 100.81 ± 11.11 mg/dL versus TNFi group: 140.93 ± 39.45 mg/dL, *p* < 0.001). These findings are summarised in S2 Table.

In a linear mixed model, we observed a significant effect of anakinra treatment on the reduction of FPG after 3 months (β: −29.08, *p* = 0.017, 95% CI −53.06 to −5.11) and 6 months (β: −39.66, *p* = 0.001, 95% CI −63.42 to −15.89). In contrast, TNFi treatment did not show significant effects on the levels of FPG in the same time period. These findings are summarised in Table 3.

**Table 3:** Analysis of FPG as clinical response, linear mixed model analysing the effect of study drugs on FPG.

FPG	β	SE	*p*	95% CI
Linear mixed model analysing the effect of study drug on overall clinical response				
Anakinra overall effect	−26.65	6.43	**<0.001**	−39.27 to −14.04
Linear mixed model unadjusted for participants’ characteristics analysing the effect of study drug				
TNFi (3 months)	−6.19	10.25	0.55	−26.29 to 13.91
TNFi (6 months)	1.43	12.84	0.91	−23.74 to 26.60
Anakinra (3 months)	−29.08	12.23	**0.017**	−53.06 to −5.11
Anakinra (6 months)	−39.66	12.12	**0.001**	−63.42 to −15.89

Statistical significance was expressed by a *p*-value < 0.05. Bolded values indicate statistically significant results.

Abbreviations: FPG, fasting plasma glucose; TNFi, tumour necrosis factor inhibitor

We analysed additional metabolic end points, but no statistical difference was observed between anakinra-treated participants and TNFi-treated participants in albuminuria, BMI, and diabetic retinopathy (Fig 2, S3 Table, S4 Table, S5 Table). During the study, we did not observe any change of diet habit and lifestyle, and we did not report any statistical difference in BMI.

### RA secondary end points

**Fig 3:**
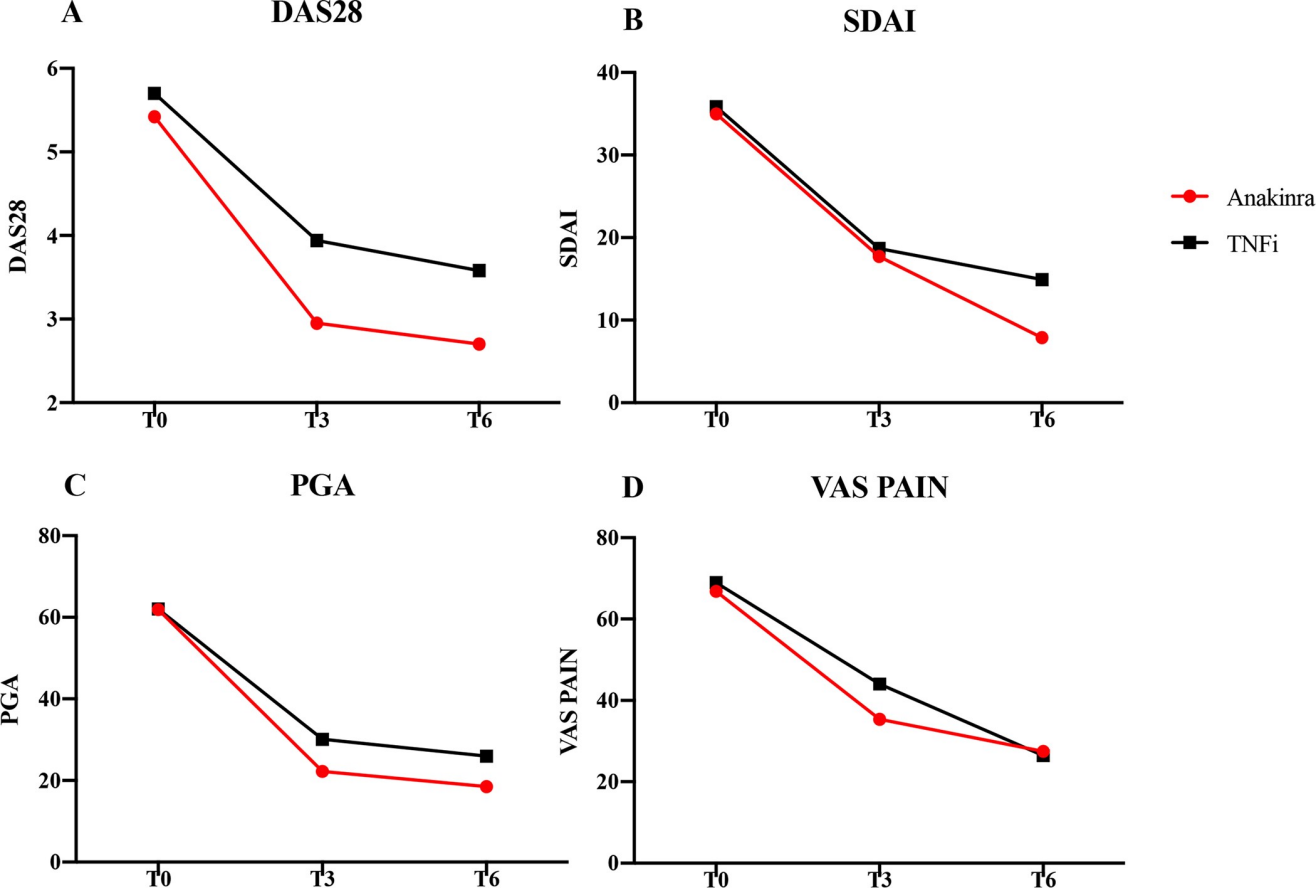
Measures of rheumatoid arthritis disease activity. Measures of rheumatoid arthritis disease activity at baseline (T0) and after 3 (T3) and 6 months (T6) of treatment with anakinra or TNFi. (A) DAS28. T0: anakinra group (5.42 ± 1.18) versus TNFi group (5.70 ± 0.80); T3: anakinra group (2.95 ± 1.58) versus TNFi group (3.94 ± 1.01), *p* = 0.039, T6: anakinra group (2.70 ± 1.16) versus TNFi group (3.58 ± 1.45), *p* = 0.08. (B) SDAI. T0: anakinra group (34.98 ± 25.18) versus TNFi group (35.86 ± 3.47); T3: anakinra group (17.70 ± 10.53) versus TNFi group (18.69 ± 29.55), *p* = 0. 90; T6: anakinra group (7.89 ± 9.23) versus TNFi group (14.93 ± 9.92), *p* = 0.0048. (C) PGA. T0: anakinra group (61.90 ± 19.17) versus TNFi group (62.00 ± 17.81); T3: anakinra group (22.21 ± 21.86) versus TNFi group (30.13 ± 19.66), *p* = 0.27; T6: anakinra group (18.53 ± 23.53) versus TNFi group (25.97 ± 19.99), *p* = 0.45. (D) VAS of pain. T0: anakinra group (66.86 ± 29.46) versus TNFi group (68.94 ± 22.86); T3: anakinra group (35.37 ± 23.74) versus TNFi group: (44.00 ± 21.98), *p* = 0.28; T6: anakinra group (27.47 ± 21.67) versus TNFi group (26.46 ± 28.38), *p* = 0.24. DAS28, Disease Activity Score-28; PGA, physician global assessment; SDAI, simplified disease activity index; TNFi, tumour necrosis factor inhibitor; VAS, visual analogue scale.

At baseline, both groups showed high disease activity without significant differences (anakinra group: 5.42 ± 1.18 versus TNFi group: 5.70 ± 0.80 mg/dL). During the study period, we observed a progressive reduction of DAS28 in both groups (Fig 3), and this reduction persisted for 6 months. These findings are summarised in S6 Table.

Analysing our results according to good EULAR response, a significant percentage of anakinra-treated participants reached a good EULAR clinical response when compared with TNFi-treated participants (anakinra group: 95.00% versus TNFi group: 62.50%, *p* = 0.030), as shown in S7 Table. Assessing the effects of the study drugs on the reduction of DAS28 after 3 months by using a linear mixed model, there were significant effects of anakinra treatment (β: −2.85, *p* < 0.001, 95% CI −3.57 to −2.12) as well as of TNFi treatment (β: −1.75, *p* < 0.001, 95% CI −2.28 to −1.22) on disease activity in both groups. The results after 6 months mirrored those observed after 3 months (anakinra: β: −3.02, *p* < 0.001, 95% CI −3.83 to −2.20; TNFi: β: −2.05, *p* < 0.001, 95% CI −2.72 to −1.38, respectively). These findings are summarised in Table 4.

**Table 4:** Analysis of DAS28 as clinical response, linear mixed model analysing the effect of study drugs on DAS28.

DAS28	β	SE	*p*	95% CI
Linear mixed model analysing the effect of study drug on overall clinical response				
Anakinra overall effect	−0.61	0.32	**0.0482**	−1.24 to −0.97
Linear mixed model unadjusted for participants’ characteristics analysing the effect of study drug				
TNFi (3 months)	−1.75	0.27	**<0.001**	−2.28 to −1.22
TNFi (6 months)	−2.05	0.34	**<0.001**	−2.72 to −1.38
Anakinra (3 months)	−2.85	0.37	**<0.001**	−3.57 to −2.12
Anakinra (6 months)	−3.02	0.42	**<0.001**	−3.83 to −2.20

Statistical significance was expressed by a *p*-value < 0.05. Bolded values indicate statistically significant results.

Abbreviations: DAS28, Disease Activity Score-28; TNFi, tumour necrosis factor inhibitor

Furthermore, we performed a correlation between HbA1c% levels and DAS28 scores after 6 months of follow-up. The Pearson correlation coefficient turned out to be 0.53 (*p* = 0.002), pointing out a monotonic effect between these 2 variables.

The SDAI values paralleled those observed for DAS28, as shown in Fig 3 and S8 Table. PGA and VAS pain values were reduced in both anakinra-treated participants and TNFi-treated participants, without any difference between the 2 arms (Fig 3, S9 Table, and S10 Table).

### Safety

The safety profile of study drugs in the TRACK trial was favourable, as shown in Table 5. No severe AEs or deaths were observed. In the anakinra group, the most common AE was urticarial lesions at the site of injection leading to discontinuation in 4 out of 22 anakinra-treated participants, suggesting the favourable safety profile. In addition, we observed nonsevere infections, including influenza, nasopharyngitis, upper respiratory tract infection, urinary tract infection, and diarrhoea, in both groups of participants. The pattern of AEs was consistent with previous reports on anakinra and TNFi [1,15], with no new safety signals identified. Interestingly, despite the reduction of HbA1c% and FPG in anakinra-treated participants, no hypoglycaemic episode was reported during the study.

**Table 5:** Safety profile of the TRACK trial.

AEs	Anakinra (*n* = 22)	TNFi (*n* = 17)
Severe AEs	0	0
Deaths	0	0
AE leading to discontinuation	4 (18%)	0
Urticarial lesions leading to discontinuation	4 (18%)	0
Urticarial lesions not leading to discontinuation	2 (9%)	2 (12%)
Influenza	1 (4%)	2 (12%)
Nasopharyngitis	0	1 (6%)
Upper respiratory tract infection	0	1 (6%)
Urinary tract infection	0	1 (6%)
Diarrhoea	1 (4%)	1 (6%)

Abbreviation: AE, adverse event; TNFi, tumour necrosis factor inhibitor; TRACK, Treatment of Rheumatoid Arthritis and Comorbidities with Kineret

## Discussion

We did an open-label, randomised, parallel-group trial in patients with RA and T2D recruited from 12 Italian rheumatologic clinics to investigate whether IL-1 inhibition could improve both glycaemic and inflammatory parameters when compared with participants treated with TNFi. When all the enrolled participants reached 6 months of follow-up, the important crude difference in the main end point, confirmed by ad interim analysis showing the significant effects of anakinra, which were not observed in the other group, led to stoppage of the study for early benefit. The study hypothesis was unexpectedly proven earlier than the predesignated timetable schedule, and with a larger percentage of anakinra-treated participants meeting the primary end point than TNFi-treated participants. Our results suggest that inhibition of IL-1 by anakinra may enable therapeutic targeting of both disorders, and use of a single agent may help in the management of both inflammatory and metabolic disease.

In our study, we observed a significant reduction in HbA1c% in anakinra-treated participants, suggesting the metabolic efficacy of IL-1 inhibition. Our findings are in line with those of a previous trial in that the anakinra-treated T2D participants showed a decrease of HbA1c and ratio of proinsulin to insulin [10]. Of interest, the extension of this study showed that this improvement of glycaemic parameters was still present 39 weeks after discontinuation of anakinra [16], confirming that anakinra is able to improve insulin secretion [4,5]. Furthermore, comparing our results to the previous study on T2D [10], we observed a more evident reduction of HbA1c%, suggesting that the inflammatory pathogenic mechanisms of T2D could be exaggerated in the context of RA. In fact, we observed a significant correlation between the decreasing levels of HbA1c% and the reduction in the disease activity. On the contrary, anakinra therapy did not show any effect in participants with type 1 diabetes (T1D), probably because the pancreatic insulitis in T1D is mainly driven by an autoimmune-mediated process, rather than by an autoinflammatory process, as suggested in T2D, thereby not supporting the benefits of IL-1 inhibition in T1D [5,17]. In fact, during T2D, the excessive levels of nutrients, including glucose and free fatty acids, stress the pancreatic islets and insulin-sensitive tissues, leading to hyperproduction of IL-β via NLRP3 inflammasome activation, a sensor of metabolic danger [4]. In addition, high-glucose concentrations induce the overexpression of the proapoptotic FAS receptor on β-cells [18]. Consequently, IL-1β and FAS may contribute, on the one hand, to the glucose-induced impairment of β-cell secretory function and, on the other hand, may lead to β-cell apoptosis [4,5,18,19]. In this study, we observed a reduction of FPG in anakinra-treated participants, paralleling the reduction of HbA1c%. Of interest, despite the improvement of HbA1c% and FPG in anakinra-treated participants, we did not observe symptomatic hypoglycaemia, as previously reported [4,10,17]. Indeed, limitations of antidiabetic treatments, such as insulin and sulfonylureas, may include the onset of unpredictable symptomatic hypoglycaemia. Conversely, it has been suggested that, following the improvement of β-cell function, by using IL-1 antagonism, these cells should release appropriate insulin amounts after metabolic stimulation, thus reducing the risk of hypoglycaemia [4,5]. Finally, the BMI of participants remained stable, thus excluding the possibility that the improvement of the metabolic parameters may be associated with a possible anorexigenic effect of anakinra.

Regarding TNFi treatment, we did not observe a statistically significant effect on HbA1c% during the follow-up. Despite the experimental evidence suggesting a possible role of TNF in regulating insulin production and function, the translation from basic studies to clinical setting failed to confirm a role for TNFi in the treatment of human T2D [20–22].

On this basis, we decided on the early stoppage of this trial for reasons relating to efficacy. The study hypothesis was unexpectedly proven earlier than the predesignated timetable schedule, and with a larger percentage of anakinra-treated participants meeting the primary end point than TNFi-treated participants. In a scenario in which the magnitude of results between the 2 groups largely exceeds the preestablished primary end point, physicians have to face the ethical dilemma between the rigidity of the protocol and the equipoise. This is the Hippocratic responsibility of physicians: to provide the optimal care to every participant [13,14,23–25]. The present trial was stopped because of the failure of equipoise, advocating the early stoppage for the large unbalanced clinical effects between the groups. In this context, WHO Declaration of Helsinki clearly states that ‘The interests of science and society should never take precedence over considerations related to the wellbeing of the subject’ [24]. The early stoppage of our study for early benefit was also supported by different statistical features, including very high Cohen size effect, more demanding O'Brien-Fleming boundary, and finally, level of statistical significance ‘of proof beyond reasonable doubt’. The latter, which could be statistically supported by the threshold of *p* < 0.001, as previously suggested [25], was largely achieved in our study. Considering all these features, because of the large and relevant clinical results concerning the difference in HbA1c%, we assessed our data in order to achieve at least a power of 80% (the lowest acceptable power) but, considering a high (disadvantageous) effect size to be reached, more demanding O’Brien-Fleming boundary (to support the early stoppage), and finally, we considered a higher level of statistical significance (*p* < 0.001 instead of 0.05) in primary end point.

In our study, we also analysed the RA clinical response, and a significant decrease in DAS28 values was observed in both groups, confirming the efficacy of both bDMARDs in RA, as already reported by meta-analytic data [1,15].

It is well accepted that CVD is the leading cause of mortality in RA. The participants enrolled in this study, with 2 independent risk factors for CVD, may have a higher CV risk [26–28]. We showed that a significant improvement of glycaemic abnormalities as well as RA disease activity in anakinra-treated participants could counteract the synergy between ‘traditional’ CV risk factors and inflammation in accelerating the atherosclerotic process. For every 1% decrease in the level of HbA1c%, the risk of CVD decreased by approximately 15% [29]. Recently, this hypothesis was confirmed in the Canakinumab Anti-inflammatory Thrombosis Outcome Study (CANTOS) trial, in which the IL-1β antagonism was able to reduce CVD in participants with previous myocardial infarction and high CRP levels, confirming the role of inflammation in CVD [30]. On these bases, the achievement of optimal therapeutic targets for both RA and T2D, as observed in our study, could consequently improve CVD risk, suggesting new therapeutic perspectives in these participants, although future long-term studies are necessary to entirely clarify this topic on CV burden of these patients.

As far as the safety profile is concerned, apart from self-limited local reactions at the injection site, no difference in the frequency of AEs between the anakinra group and the TNFi group was observed. The results concerning safety mirrored meta-analytic data without new identified signals [25,31,32]. In the last decades, the poor compliance, mainly due to daily injections and urticarial lesions, reduced the clinical usability of anakinra [1,25]. However, recently, new technologies are being tested to increase the half-life of the drug, enhancing the possible competitiveness in clinical setting. The processes of PEGylation and HESylation, the attachment of polyethylene glycol (PEG) or hydroxyethyl starch (HES) to a drug molecule, have shown to increase the half-life of anakinra without any effect on the protein’s secondary structure, thus developing a possibly more suitable molecule to be used in clinical setting [33].

Our study has some limitations, mainly due to the open-label design and a previously unplanned ad interim analysis, which is more prone to biases compared with a double-blind controlled trial. According to the design of the study and Italian law, in which only well-established routine tests for the management of T2D and/or CVD were allowed, we could not plan some evaluations, such as C peptide, insulin endogenous plasma IL-1Ra, as well as laboratory markers of endothelial dysfunction, which would be of interest [10,16,34–36]. In addition, because of the ‘real-life’ study design, the ongoing use of other drugs could affect the outcome, such as CCSs and MTX, although conflicting results are available [37–41]. Because of the magnitude of the results in the anakinra group, we would speculate that such a reduction could not be influenced by the open-label design and the lack of a placebo arm. In this, the very large magnitude of primary end point, which we observed, cannot be discarded. The probability to observe such an effect, assuming the groups are therapeutically indifferent, cannot be imputed to mere chance, according to the type I error assumed. In addition, despite all the penalties we applied to the analysis of our data, the results maintained their statistical significance in spite of the low number of enrolled patients. Furthermore, in a real-life setting, the randomisation to placebo of participants affected by an active disease could raise some ethical issues, limiting the possible benefit of well-known standard therapies. The results of our study, despite the low number of enrolled participants, showing that the primary end point was achieved only in the anakinra group, partially solved this scientific issue.

Looking forward to the era of personalised medicine, a better profile of patients could allow the physician to select the best therapeutic strategy in order to improve the clinical benefits, thus reducing the potential failure of treatment [42]. Our study suggests that, in patients with RA and T2D, anakinra could be considered a targeted treatment, leading to an improvement of metabolic parameters as well as inflammatory signs, tailoring the medical treatment to the individual characteristics [43–45]. Furthermore, considering the confirmed effect of IL-1 inhibition in the prevention of CVD [30], it is possible to suggest that IL-1 inhibition may decrease the burden of CV risk in RA. The results of our study could also open the way for subsequent confirmatory studies analysing the efficacy of therapeutic strategy targeting IL-1 in RA with T2D. In fact, it must be pointed out that despite the newer antidiabetic therapeutic strategies, almost 30% of patients with T2D are currently treated with insulin because the duration of diabetes is still a strong, independent determinant of insulin use, still lacking disease-modifying drugs [46,47]. Finally, new IL-1-inhibiting agents, including canakinumab, gevokizumab, and rilonacept, which are not associated with the discomfort of daily injection, already showed some efficacy in RA and T2D [6,36,46,48–50].

In conclusion, results of this study suggest a positive effect of IL-1 inhibition in patients with RA and T2D, reaching the therapeutic targets of both diseases and improving the main outcome of enrolled participants. Anakinra-treated participants reached the primary end point (decrease of HbA1c%) in a very short time. No significant decrease of HbA1c% was observed in TNFi-treated participants. Our results suggest that IL-1 inhibition may be considered as a targeted treatment for people with both RA and T2D. Based on our pilot study, future studies are needed to further assess the use of IL-1-inhibiting agents in patients with both RA and T2D and to assess long-term outcomes on CVD. Future studies might include the possible use of these drugs in monotherapy and as disease-modifying drugs and of the timing of the therapy.

### Protocol

The original protocol of the trial is accessible in S1 Text and S2 Text.

## Supporting information

S1 CONSORT Checklist CONSORT checklist. CONSORT, Consolidated Standards of Reporting Trials.
(DOCX)

S1 Fig General trial diagram. (PDF)

S1 Text English version of the study protocol. (DOC)

S2 Text Italian version of the study protocol. (DOC)

S1 Table Mean values of HbA1c in anakinra- and TNFi-treated participants. HbA1c, glycated haemoglobin; TNFi, tumour necrosis factor inhibitor.
(DOCX)

S2 Table Mean values of FPG in anakinra- and TNFi-treated participants. FPG, fasting plasma glucose; TNFi, tumour necrosis factor inhibitor.
(DOCX)

S3 Table Mean values of albuminuria in anakinra- and TNFi-treated participants. TNFi, tumour necrosis factor inhibitor.
(DOCX)

S4 Table Mean values of BMI in anakinra- and TNFi-treated participants. BMI, body mass index; TNFi, tumour necrosis factor inhibitor.
(DOCX)

S5 Table Diabetic retinopathy in anakinra- and TNFi-treated participants. TNFi, tumour necrosis factor inhibitor.
(DOCX)

S6 Table Mean values of DAS28 in anakinra- and TNFi-treated participants. DAS28, Disease Activity Score-28; TNFi, tumour necrosis factor inhibitor.
(DOCX)

S7 Table Percentage of participants reaching good EULAR clinical response and remission. EULAR, European League Against Rheumatism.
(DOCX)

S8 Table Mean values of SDAI in anakinra- and TNFi-treated participants. SDAI, simplified disease activity index; TNFi, tumour necrosis factor inhibitor.
(DOCX)

S9 Table Mean values of PGA in anakinra- and TNFi-treated participants. PGA, physician global assessment; TNFi, tumour necrosis factor inhibitor.
(DOCX)

S10 Table Mean values of VAS of pain in anakinra- and TNFi-treated participants. TNFi, tumour necrosis factor inhibitor; VAS, visual analogue scale.
(DOCX)

S1 Data Minimal data set with the values behind the means and used to build graphs. (XLS)

## Abbreviations

ACPA: anticyclic citrullinated peptide antibody
ACR/EULAR: American College of Rheumatology/European League Against Rheumatism
ADA: American Diabetes Association
AE: adverse event
ALAT: alanine aminotransferase
ARR: absolute risk reduction
ASAT: aspartate aminotransferase
bDMARD: biologic disease-modifying antirheumatic drug
BMI: body mass index
CANTOS: Canakinumab Anti-inflammatory Thrombosis Outcome Study
CCS: corticosteroid
CER: control event rate
CONSORT: Consolidated Standards of Reporting Trials
CRP: C-reactive protein
CV: cardiovascular
CVD: CV disease
DAS28: Disease Activity Score-28
EER: experimental event rate
ESR: erythrocyte sedimentation rate
FPG: fasting plasma glucose
HbA1c%: percentage of glycated haemoglobin
HCQ: hydroxychloroquine
HES: hydroxyethyl starch
IL-1: interleukin-1
IR: insulin resistance
MedDRA: Medical Dictionary for Regulatory Activities
MTX: methotrexate
NLRP3: nucleotide-binding oligomerization domain-like receptors-, leucine-rich repeat-, and pyrin domain–containing 3
NNT: number needed to treat
NYHA: New York Heart Association
PGA: physician global assessment
PEG: polyethylene glycol
RA: rheumatoid arthritis
SDAI: simplified disease activity index
SOC: system organ class
SSZ: sulfasalazine
T1D: type 1 diabetes
T2D: type 2 diabetes
TNF: tumour necrosis factor
TNFi: TNF inhibitor
TRACK: Treatment of Rheumatoid Arthritis and Comorbidities with Kineret (anakinra)
VAS: visual analogue scale.
