# Supplementary figures and images for: Anti-interleukin-1 treatment in patients with rheumatoid arthritis and type 2 diabetes (TRACK): A multicentre, open-label, randomised controlled trial
Source: PLoS Med. 2019 Sep 12;16(9):e1002901. doi: 10.1371/journal.pmed.1002901 (PMC6742232; doi:10.1371/journal.pmed.1002901)

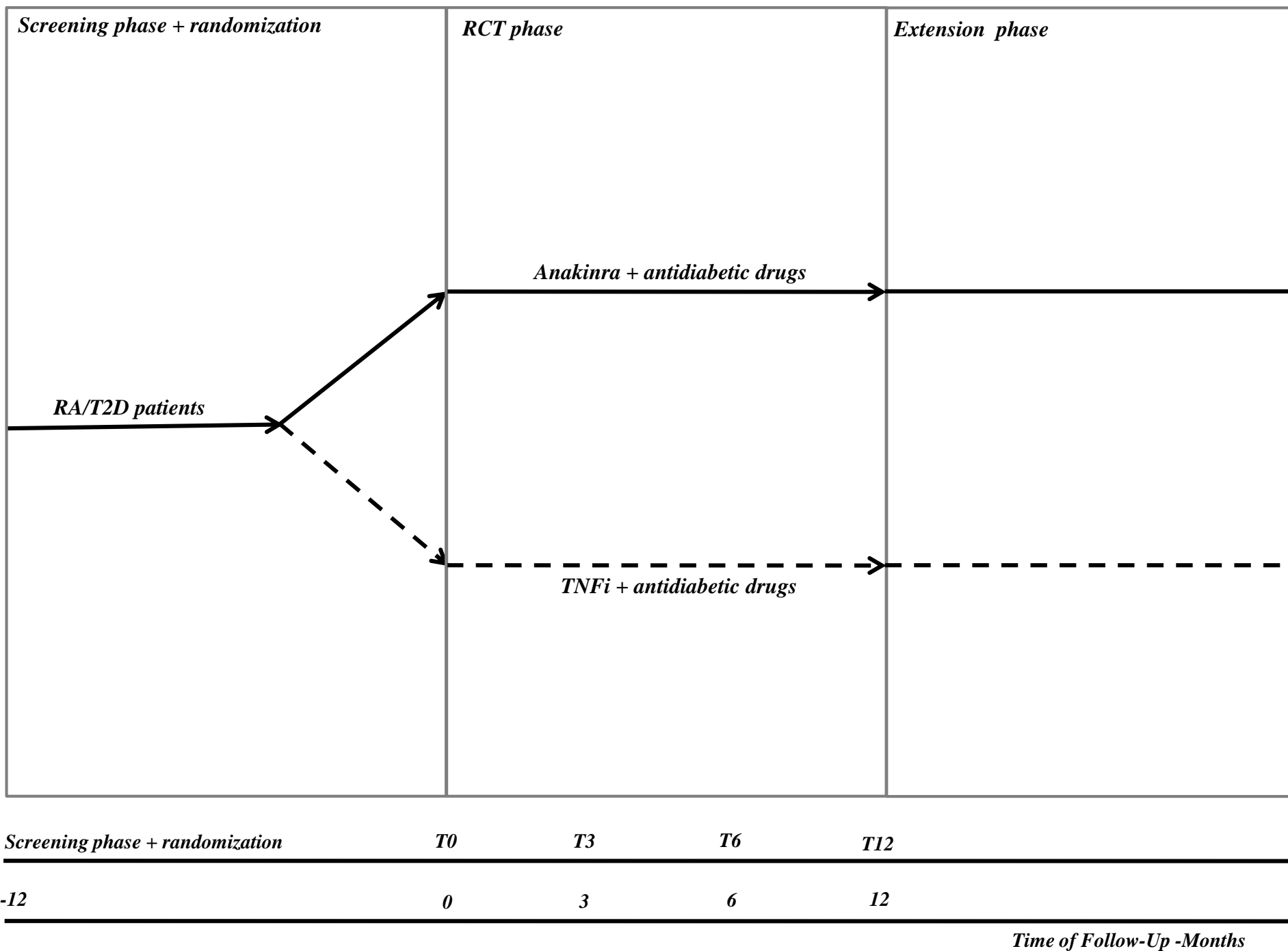

Supplement: S1 Fig — (PDF) [file pmed.1002901.s002.pdf]
