# Supplementary material for: Anti-interleukin-1 treatment in patients with rheumatoid arthritis and type 2 diabetes (TRACK): A multicentre, open-label, randomised controlled trial
Source: PLoS Med. 2019 Sep 12;16(9):e1002901. doi: 10.1371/journal.pmed.1002901 (PMC6742232; doi:10.1371/journal.pmed.1002901)
Supplement: S1 Text — (DOC) [file pmed.1002901.s003.doc]

**No Profit Study**

**(Decreto Ministero della Salute 17 dicembre 2004)**

**Title**

**"NO-PROFIT" CLINICAL STUDY FOR THE IMPROVEMENT OF CLINICAL PRACTICE, TO EVALUATE THE EFFICACY OF ANAKINRA IN THE REDUCTION OF GLYCATED HEMOGLOBIN IN SUBJECTS WITH RHEUMATOID ARTHRITIS AND DIABETES; RANDOMIZED, CONTROLLED, OPENED CLINICAL STUDY FOR PARALLEL GROUPS.**

*Sponsor and principal investigator****:***

Division of Rheumatology,

Department of Biotechnological and Applied Clinical Sciences,

University of L'Aquila,

L'Aquila, Italy

### Product: Anakinra (Kineret®)

### Protocol: ANAKIN-RA-DIABETES

### Version: Final

### Date: 15.11.12

*The main procedural details of the trials

*Key details of the planned statistical analyses, including interim analysis and criteria for stoppage defined in the protocol

*Details of the power calculation used to estimate the sample size

# INTRODUCTION

## Rationale

Rheumatoid arthritis (RA) is the most common inflammatory arthritis and affects about 0.5% of the adult population. It is characterized by a progressive inflammation of the synovial membrane which causes joint tenderness and pain. Over time there is a progressive structural damage of affected joints, which can be highlighted radiologically and can reduce joint function. The most promising targets for therapeutic intervention are two inflammatory cytokines, the tumor necrosis factor-α (TNF-α) and interleukin-1 (IL-1); they represent the critical factors for the initiation and progression of synovial membrane inflammation and for the degradation of the joint matrix (1).

Currently, the research is focused on the regulation mechanisms of cytokines that act to maintain or restore their homeostasis, both in physiological and pathological conditions. These "natural inhibitors", made available by cloning and gene expression in bacteria or eukaryotic cells, can be used to support or amplify the effects of the activity of endogenous cytokine inhibitors, thus presenting the possibility of correcting the imbalance in the regulation of these molecules in physiopathological conditions.

Over the last few decades, it has become increasingly clear that chronic immune system activation, as in the case of AR pathogenesis, is associated with changes in the intermediate metabolism, and potentially leads to an increased risk of cardiovascular disease (CVD) (2). Several reports have discussed the association between the status of chronic inflammation and metabolic disorders, specifically with peripheral insulin resistance (IR) (3). In patients with RA, the reduction of muscle tone and the sedentary lifestyle related to the disease can probably further contribute to inducing the development of IR. Although we could expect the prevalence of type 2 diabetes (DM2) to increase in patients with RA, several cross-sectional studies have not established a definite association between these two conditions. In addition, several independent studies have indicated that RA, IR and type 2 diabetes (T2D) are associated with the presence of molecules able to act on different immunoregulatory pathways, including TNF-α, interleukin-6 (IL-6) and IL- 1 (4-5).

Experimental studies on humans and animals suggest that T2D is associated with an imbalance between IL-1 and the factors that antagonize its activity; in particular, an abnormal production of IL-1 by beta cells occurs, due to the glucose-induced activation of the caspase-1 inflammosome and to the decrease of the IL-1 receptor antagonist synthesis (IL-1Ra). The idea that in T2D a relative deficiency of IL-1 antagonist may lead to glucose-induced malfunction of beta cells is confirmed by clinical studies demonstrating that, in these patients, the IL-1 antagonism by using anakinra (a recombinant and slightly modified version of the human IL-1Ra) is able to improve glycaemia and beta cell function. In fact, in the course of T2D, IL-1Ra induces the reduction of both glycated hemoglobin (A1c) and glycaemia and improves the function of beta cells, but does not affect body weight or body mass index and insulin sensitivity.

Recently, Larsen et al. (6) confirmed the above reported data on the ability of anakinra to improve glycemic control in patients with T2D probably due to an improvement of the secretory function of beta cells. In fact, it has been shown, in patients treated by anakinra, an improvement of glycaemia and of secretory capacity of beta cells. However, in the same patients, no changes in insulin sensitivity evaluated by hyperglycemic-hypoglycemic clamp, insulin sensitivity indices based on glucose tolerance test, insulin-regulated gene expression in skeletal muscle or serum adipokines levels were observed. Finally, the body mass index (BMI) levels of the patients did not change thus excluding an anorexigenic effect of the drug. However, the authors did not rule out that higher doses of anakinra may improve insulin sensitivity.

## Study drug

Anakinra (Kineret®) is a recombinant molecule consisting of the synthetic, non-glycosylated form of the IL-1Ra antagonist, produced in E.Coli cells. It mimics the endogenous mechanism underlying IL-1 regulation. It has a molecular weight of 17.3 kilodaltons and is composed of 153 amino acids. The main difference between human endogenous Kineret® and IL-1Ra is the inclusion of a single methionine residue in the terminal amino acid of Kineret®. Kineret® has been designed to reduce the imbalance in IL-1 metabolism; it acts by competitively inhibition of the binding between IL-1 and its type 1 receptor (IL-1R1), thus blocking its biological activity. This process mimics the activity of the endogenous IL-1Ra.

## Risk/benefit ratio

The Committee for Medicinal Products for Human Use (CHMP) has determined that the benefits of Kineret® outweigh its risks, in the treatment of signs and symptoms of rheumatoid arthritis, in combination with methotrexate, in patients with inadequate response to methotrexate alone.

The Committee recommended the granting of the marketing authorization for Kineret® (EPAR - EMEA/H/C/363).

# AIMS

The purpose of this no-profit clinical study is to verify whether the administration of anakinra in patients with RA with comorbid T2D is able to determine an improvement in glycemic control as well as of signs and symptoms of RA, thus determining an improvement in the clinical practice of the use of this drug, which could control both the morbidity of such patients.

During the course of the study, anakinra will be used following the authorized indication and dosage, as prescribed by the Summary of Product Characteristics (SmPC), with the aim of improving the normal clinical practice.

# EXPERIMENTAL DESIGN

## Study endpoints

Primary endpoints

The predefined primary end point waill be the change in the percent A1c levels between baseline and subsequent visits. According to available literature, an absolute difference of 0.25 was considered to be statistically significant between the two arms (Larsen 2007).

*Secondary endpoints*

A number of secondary endpoints will be planned to evaluate the effect of study drugs on RA signs and symptoms and additional metabolic parameters in baseline and subsequent visits.

*RA features:*

disease activity score in 28 joints (DAS28)

simplified activity score (SDAI),

percentage of patients reaching good EULAR clinical response,

percentage of patients reaching remission,

physician global assessment (PGA),

visual analogue scale (VAS) of pain,

erythrocyte sedimentation rate (ESR),

C-reactive protein (CRP).

*T2D features:*

fasting plasma glucose (FPG),

microalbuminuria,

the presence of retinopathy,

BMI.

***Safety***

Safety of the treatment was evaluated during all scheduled visits, and any suspected adverse event (AE) was recorded and coded according to the Medical Dictionary for Regulatory Activities (MedDRA) system organ class (SOC) classification. Compliance with study drugs was assessed by checking the patient’s diary, in which patients were asked to record each administration of medications.

## Study design

This study is designed as a "no-profit" clinical study for the improvement of clinical practice, randomized, controlled, for parallel groups, in open, multicentre trial to verify the possibility that the administration of anakinra (Kineret®) could lead to an improvement in glycemic control as well as signs and symptoms of RA in patients with RA and T2D as comorbidity. Patients will be randomized into two treatment arms. In both arms the drugs will be used in the indication and in the authorized dosages, according to the normal clinical practice. Anakinra will be administered to the arm being treated with the study drug, while the control group will be given another drug used to treat RA as part of the TNF-α inhibitor class. The study includes an initial screening phase and a 24-month treatment period.

## Randomization

The research was designed as a multicentre, randomised, open, prospective, controlled, parallel-group study. Patients were assigned to each treatment group using the method of randomized block randomization to control for possible unbalancing excess between trial arms. The randomization list will be generated by the coordinating centre. The trial was open label (not masked) for all participants, investigators and the statistician.

# STUDY POPULATION

## Sample Size

See statistical analysis (see below)

## Inclusion/Exclusion Criteria

### Inclusion/exclusion criteria

Eligible participants must fulfil any of the following inclusion criteria: male and female patients aged ≥18 years; affected by RA, classified according to American College of Rheumatology/European League Against Rheumatism (ACR/EULAR) criteria; with moderate to severe RA, with an inadequate response to previous treatment with methotrexate (MTX) characterised by a Disease Activity Score-28 (DAS28) >3.2; affected by T2D, classified according to American Diabetes Association (ADA) criteria and of at least six months duration; with glycated haemoglobin (A1c) >7%<10% and a body mass index (BMI) <35. For patients previously treated with a biologic DMARD, an appropriate washout period, according to the relevant datasheets, was mandatory; stable and not increasing corticosteroids (CCSs) therapy, at the lowest effective dosage and, furthermore, not more than 7.5 mg/daily of prednisone-equivalent would be allowed. Patients treated with anti-rheumatic drugs at baseline could not change drug administration and dosage regimen for at least one month before enrolment. Similarly, patients treated with antidiabetic drugs at baseline could not change drug administration and dosage regimen for at least three months before enrolment.

Eligible participants must not fulfil any of the following inclusion criteria: T2D diagnosed more than ten years prior to the study; ongoing acute or chronic infection; increased (>30 mg/L) levels of C-reactive protein (CRP); fever; ongoing antibiotic therapy; chronic granulomatous infections, such as tuberculosis; history of recurrent infections; C-peptide values <0·5 ng/mL (0·1665 nmol/L); presence of neutropenia (white blood count <2000/mm^3^) or anaemia (haemoglobin <11g/dL for men and 10g/dL for women); presence of one or more contraindications reported in the datasheet of study (ANA) or control (TNFis) drugs; presence of one or more contraindications to MTX; previous ischaemic attack or myocardial infarction; heart failure of New York Heart Association (NYHA) class III or IV; hepatic or progressive liver disease (values of alanine aminotransferase/aspartate aminotransferase [ALAT/ASAT] elevated by at least two-fold compared with normal values); pregnancy, or women not using contraceptive measures; breast-feeding; participation in another clinical study up to six months before randomisation; depressive syndrome or other serious psychiatric illness; presence of known malignancy; clinically significant history of alcohol abuse or drug addiction; any condition that, in the opinion of the investigator, could preclude the possibility of use of study drugs in compliance with datasheet indications; any other condition or laboratory parameter that, in the opinion of the investigator, could preclude the participation of the subject in the study.

## Criteria for the withdrawal of the subjects from the study

Patients should be informed of the possibility of interrupting the study at any time they consider appropriate. Each interruption must be fully documented in the CRF by the investigator.

The Investigator has to make every reasonable effort to keep the Patient in the study protocol. However, if it is necessary to exclude the Patient, a complete conclusive assessment should be performed. All results of this evaluation and a description of the reasons that led to the exclusion of the patient have to be recorded in the CRF.

Patients may discontinue the study prematurely if any of the following conditions occur:

• Patient request, even if not motivated;

• Withdrawal of consent by the Patient;

• Appearance of serious adverse reactions which, in the opinion of the Investigator, make the continuation of the study impossible;

• Violation of the study protocol which, in the opinion of the investigator, jeopardize the participation of the Subject to the study;

• Any situation in which the investigator believes that exclusion from the study is in the patient's best interest;

• Occurrence of exclusion criteria.

As far as possible, withdrawal from the study should take place after mutual consultation. In the case of early closure all documentation must be returned to CRO Pharmaceutical Development and Services srl.

It will be the responsibility of the Investigator to follow the Patients for an appropriate period of time, in relation to the event that determined their withdrawal from the study, in order to verify the clinical conditions, any laboratory tests, and/or the occurrence of adverse events also after some time from the cessation of therapy. The involved Authorities must be informed.

Data from patients who discontinued the study will be included in the overall assessment of the efficacy of the drug and its tolerability.

# TREATMENTS OF SUBJECTS

Both the study drug and the control drugs will be used in the context of normal clinical practice, in accordance with the indication and according to the dosages provided for by the respective CPR.

In the present study, the effect of anakinra (Kineret®) on the reduction of glycated hemoglobin is evaluated, while there is no scientific evidence regarding an effect of control drugs (TNF-α inhibitors) on this parameter. TNF-α inhibitors are instead the "usual care" in the treatment of patients with RA. Consequently, only anakinra (Kineret®) is considered the "product under experimentation", in line with the guidelines for Good Clinical Practice (Ministerial Decree dated 15 July 1997, Annex 1 Glossary Paragraph 1.33).

## Treatments

### Dosages and administration routes

Patients will be randomized into two treatment groups.

Subjects treated by study drug:

Subjects will be treated with anakinra (Kineret®) 100 mg/die subcutaneously; in accordance with the provisions of the datasheet. The drug will be administered by self-injection subcutaneously into the abdomen or thigh.

All Patients will be instructed by the study staff on the procedure for the correct administration of the drug with sterile subcutaneous injection. The administration of the first dose will take place in the experimental center under direct control of the staff of the center. This supervision of the Investigator will allow to verify the Patient's safety and the appropriate technique of subcutaneous injection so that all subsequent doses can be administered outside the center.

Subjects treated by control drugs:

### Subjects will be treated with a drug belonging to the TNF-alpha inhibitor class (ATC L04AB); these drugs represent an "usual care" treatment for patients with RA; the choice of the specific medicinal product is left to the investigator’s judgment. The dosage and method of administration must comply with the datasheet of the administered drug.

### Concomitant treatments

- *Methotrexate*

All Patients will also be given methotrexate, in accordance with the datasheet at a dose of between 15 and 20 mg per week (according to the investigator).

- *Anti-diabetic treatments*

Subjects will be invited to maintain the eating habits and lifestyle in progress at the time of randomization.

At the time of enrollment, the subjects will maintain the hypoglycemic pharmacological treatment in progress before the study. During the course of the study, hypoglycemic treatment can be only reduced according to the guidelines of the investigator. No increase of anti-diabetic therapy will be allowed.

- *Treatment with NSAIDs and CCSs*

### At the time of enrollment, Subjects will maintain treatment with steroidal and non-steroidal anti-inflammatory drugs (NSAIDs) in progress prior to the study. During the course of the study, treatment with NSAID may be changed at the discretion of the physician; the study is discontinued if the dosage of steroidal anti-inflammatory drugs is increased. CCSs will be allowed at the lowest effective dosage and, furthermore, not more than 7.5 mg/daily of prednisone-equivalent/die.

### Other treatments

### For the duration of the study all subjects will receive 5 mg of folic acid every week. Each treatment that the Subject underwent in the month before to the inclusion in the study, at the time of enrollment or who receives during the study must be recorded in the medical record and reported in the CRF. Any variation of concomitant treatments during the study period must also be recorded. For any concomitant treatment, the following should be indicated: the name of the active substance, the dose, the start and end date of treatment.

Special warnings and precautions for use

See datasheet of study drug (Kineret® datasheet)

Packaging, labeling of the drug

The study is open-label; the Kineret® medicinal product will be administered. The packs used during the study are those intended for commercial use (see Kineret® datasheet).

Anakinra will be supplied in pre-filled syringes (100 mg Anakinra in 0.67 ml) for self-injection. The drug will be administered subcutaneously in the abdomen or thigh. The unused medicinal product and waste derived from this medicinal product must be disposed of in accordance with local regulations.

Storage of the drug

### Store in a refrigerator (2 ° C - 8 ° C). Do not freeze. Store in the original package in order to protect from light (see Kineret® datasheet).

Distribution of the drug to patients

Being a study for the improvement of normal clinical practice, in accordance with the provisions of art. 2, paragraph 1 of *Decreto Ministero della Salute 17 dicembre 2004* (“no-profit study”) the drug remains the responsibility of the National Health System. The drug will be provided to the patient according to the procedures provided by the various hospital centers participating in the study. The investigator has the obligation to keep accounts of all the drug administered to patients for the duration of the study, using the prepared forms.

### Drug Accountability

The investigator will invite the participants to the study to fill in a specific diary in which the drug will be recorded. The diary will be used for drug accountability. There is no provision for patients to return empty and unused packs of medicines.

## Compliance

The Investigator should instruct patients to report the diary attesting to taking the study drug at each check-up. The assessment of Patient compliance will be performed by evaluating the data reported in the diary. This count must be reported in the appropriate section of the CRF.

## Study follow-up

The study protocol is designed on 24 months of follow-up.

## Interruption/Discontinuation of the treatment

The Patient must stop the treatment, and follow the standard procedures established by par. 4.3 of the protocol, if after 6 months of treatment the following cases occur:

• Patients do not achieve clinical remission of RA or at least moderate clinical improvement (according to the investigator's assessment), which allows a satisfactory balance in quality of life;

• Patients show a worsening of the glycemic balance (according to the investigator's evaluation).

The study is discontinued if the dosage of steroidal anti-inflammatory drugs is increased.

# STUDY PROCEDURES

## Primary Endpoint

The primary endpoint of the study is the change in the level of A1c, compared to baseline conditions, after 3 months, 6 months, 12 months and 24 months.

The results of the A1c should be reported using the IFCC (mmol/mol) units and the NGSP derived units (%), using the IFCC-NGSP general equation to perform the conversion. The relationship between the two modes of expression of the result is as follows (7):

A1c (%) = 0.0915 × HbA1c "aligned IFCC" (mmol mol) + 2.15

## Secondary endpoints

### DAS28

Evaluation of the percentage of patients in remission and improved patients using the DAS28 evaluation scale (*EULAR criteria*). The DAS28 is an evaluation index of RA. It is calculated using the formula that includes the number of swollen joints and the number of tender joints (28 joints):

*DAS28 = 0.56 • √ (t28) + 0.28 • √ (sw28) + 0.70•Ln(ESR) + 0.014•GH*

where:

t28 = number of tender joints over 28

sw28 = number of swollen joints over 28

Ln (ESR) = Natural logarithm of ESR (mm / hour)

GH = Overall health status (visual-analogue scale)

The DAS is applicable to the assessment of disease activity at a given time and to the assessment of the change in the time of disease activity.

### The DAS28 evaluation criteria are as follows:

### improved patient: reduction in disease activity score from> 5.1 (high disease activity) to <3.2 (low disease activity) or from <3.2 (low disease activity) to <2.6 (remission).

### patient in remission: score <2.6

### SDAI

Evaluation of the percentage of patients in remission and of patients improved using the SDAI evaluation scale (*EULAR criteria*). The SDAI is a simplified measure for the calculation of disease activity. It is obtained from the arithmetic sum of 5 parameters: number of tender joints (0-28), number of swollen joints (0-28), overall assessment of the activity of the disease by the patient by VAS (0-10 cm), global evaluation of the activity of the disease by the doctor using VAS (0-10 cm), serum concentration of the CRP.

The SDAI evaluation criteria are as follows:

- Improved patient: score reduction from> 26 (high disease activity) to </ = 11 (low disease activity)
- Patient in remission: <3.3

### Count of swollen joints

66 joints will be evaluated; the number of swollen joints will be reported which will be classified as "present" or "absent" on the evaluation form. Only 28 specific joints will be considered as a calculation for DAS28 and SDAI. The joints treated pharmacologically by intra-articular injection will not be evaluated for a time-period of 3 months after treatment.

### Count of tender joints

68 articulations painful of passive mobilization or pressure on the articular line will be evaluated expressing the pain in the joints as "present" or "absent" in the evaluation form. Only 28 specific joints will be considered in the DAS28 calculation. The joints treated pharmacologically by intra-articular injection will not be evaluated for a period of 3 months after treatment.

### VAS Patient

Evaluation of the activity of the disease by the patient with visual analog scale (VAS). The Patient will be given a sheet with a horizontal line (not graduated, 10 cm long, with writing on the left "absent" side and on the right end "maximum activity") and He will be asked to mark on this line the intensity of the just pain.

### PGA physician

Evaluation of the disease's activity by the Investigator using physician global assessment (PGA). The Investigator's judgment will be evaluated on the overall degree of activity of the disease expressed by visual analogue scale referring to the previous clinical survey. The physician will be given a sheet with a horizontal line (not graduated, 10 cm long, with written on the left end "absence of activity of the disease" and at the right end "maximum activity of the disease); the investigator will have to mark in this line the overall degree of activity of the disease.

### VAS Pain

#### Pain assessment with visual analog pain scale (VAS). The Patient will be given a sheet with a horizontal line (not graduated, 10 cm long, with writing on the left "absent" side and on the right "maximum pain") and he will be asked to mark on this line the intensity of the pain.

Health assessment questionnaire (HAQ)

The Stanford Health Assessment Questionnaire HAQ; The Italian version of the functional disability index of the health assessment questionnaire. A reliable instrument for multicenter studies on rheumatoid arthritis. *Ranza et al Clin exp Rheumatol 1993* is one of the components of the ACR criteria and assigns a score to the ability to perform daily activities from 0 (low disability) to 3 (very severe disability). The HAQ questionnaire and the criteria for the assignment of the score are shown in the attachment (Annex 3).

Laboratory parameters for the evaluation of the RA:

The following parameters will be measured using the methods normally used in the laboratories of the experimental centers:

• CRP (C-reactive protein);

• ESR (erythrocyte sedimentation rate);

• rheumatoid factor;

• anti-citrulline antibodies (anti-CCP) evaluated by second generation tests, CCP2/ELISA or a third-generation test, CCP3/ELISA.

Laboratory parameters for the evaluation of the T2D:

The following parameters will be measured using the methods normally used in the laboratories of the experimental centers:

• fasting blood glucose, triglycerides, total cholesterol, HDL cholesterol and LDL cholesterol.

• microalbuminuria assessed by the albumin/creatinine ratio on a sample of urine collected in the morning.

Evaluation of diabetic retinopathy

Retinopathy will be assessed by ocular fundus examination, in accordance with the provisions of the AMD 2002 guidelines.

### Body Mass Index (BMI)

The body mass index will be evaluated using the following formula:

BMI = weight (kg) / height^2^ (m^2^)

### Safety Endpoints

#### Evaluation of vital signs and objective examination;

#### Evaluation of laboratory and instrumental parameters;

#### Evaluation of the type, frequency and intensity of adverse events (AE) according to the procedure defined by the study protocol (see chapter 7);

#### Evaluation of hypoglycemic episodes, classified as:

#### *Severe:* an episode that endangers the patient's life and requires immediate medical intervention;

#### *Moderate:* plasma glucose levels <56 mg / dl and / or presence of symptomatology that does not require immediate medical intervention;

#### *Mild:* plasma glucose levels> 56 mg / dl or presence of symptoms that did not require blood glucose measurement; it will also be indicated if the hypoglycemic episode will occur during the night between 23:00 and 5:59 inclusive.

## Additional laboratory assessment

### In addition to the tests already listed in section 6.2, the following laboratory tests will be carried out.

Hemochromocytometric examination with leukocyte formula and platelet count; blood azotemia (BUN), creatinine, bilirubin, transaminase (ALAT/ASAT), alkaline phosphatase, sodium, potassium, calcium, uric acid, total proteins, albumin; complete urinalysis.

All the aforementioned tests will be performed using the methods normally used in the laboratories of the experimental centers.

Blood sampling to be performed after the evaluation of pain, the administration of the questionnaires and the evaluation of vital signs.

## Scheduled Visits

Each patient will be subjected to 6 visits:

Visit 0 (Screening visit);

Visit 1 - month 0 (Study visit, within 10 days from visit 0);

Visit 2 - month 3 (3 months after visit 1);

Visit 3 - month 6 (after 6 months from visit 1);

Visit 4 - month 12 (after 12 months from visit 1);

Visit 5 - month 24 (Final visit, 24 months after visit 1).

For each patient, the duration of the study will be 24 months in total.

***Visit 0 (Screening visit)***

During the visit 0, the Patient selection will take place. The Investigator, after having thoroughly informed the Patient and after having left him all the time necessary for the clarifications of the case, will invite him to sign the Informed Consent. After signing the Informed Consent, the Patient will be subjected to a medical examination, blood and urine samples, and specific instrumental tests to verify compliance with the inclusion/exclusion criteria.

Specifically, the following evaluations will be carried out:

• Signing of informed consent;

• Demographic data (initials, date of birth, sex, height, weight);

• History;

• Evaluation of vital signs (arterial pressure, heart rate, body temperature);

• Objective examination;

• BMI;

• Clinical evaluation of RA by using: DAS28; SDAI; count of tender joints (66 joints); counts of swollen joints (68 joints); HAQ.

• Biochemical evaluation of RA by: CRP, ESR, anti-CCP, anti-dsDNAs, ANAs, rheumatoid factor;

• A1c evaluation;

• Fasting blood glucose, triglycerides, total cholesterol, HDL cholesterol, LDL cholesterol;

• Specific test for the diagnosis of tuberculosis (PPD and / or QFT);

• X-ray examination of the thorax (not necessary if already done in the 3 months before to the visit);

• 12-lead electrocardiogram;

• Evaluation of signs and symptoms of ischemic heart disease;

• Hemochromocytometric examination with leukocyte formula and platelet count;

• Clinical chemistry examinations;

• Complete examination of urine and microalbuminuria;

• Pregnancy test;

• Recordings of concurrent diseases;

• Registration of concomitant pharmacological treatments and / or those undertaken by the patient in the 3 months before to the enrollment visit.

Some of the above evaluations may not be carried out if the Patient has already the results. The usability of the results already in possession of the patient for the purposes of the study will be left to the judgement of the Investigator, with the exception of the chest-X ray (which must have been carried out in the 3 months prior to the visit). At the end of the visit, the Patient will be invited to show up for the next visit (Visit 1 - Study visit), which have to be done after the Investigator and/or the Patient will be in possession of all the results of the evaluations carried out in the Visit 0, and in any case no later than 10 days from the date of the Visit 0.

***Visit 1 (Study start visit - month 0)***

This visit has to be made no later than 10 days from visit 0. During the visit 1, the results of the laboratory tests and the instrumental examinations carried out during the visit 0 will be verified. Subsequently the Investigator, after checking that all the inclusion criteria are respected and no exclusion criteria are occurred, will proceed to enrollment of Patients deemed suitable and their randomization in one of the two treatment groups.

After randomization, Patients will undergo a series of procedures and, subsequently, the Patient's Diary will be delivered to take note of the drug intake, the patient will be instructed by the investigator on completing the diary. The first dose of the investigational drug and the control drug will then be administered; the Patient will be instructed by the investigator on the methods of self-administration of drugs. The administration of the first dose of drugs should be recorded in the CRF and in the Patient's diary. The investigational drug and the control drug will be delivered to the patient, through the hospital pharmacy or according to the procedures established by the procedures in force at the various experimental centers.

The Investigator will invite the patient to new visit after 3 months (± 7 days) from the date of visit 1 taking with him the patient's diary; he will also instruct the patient to promptly contact him in the eventuality of the occurrence of disorders of any kind.

Specifically, during the visit 1, the following evaluations will be carried out:

• Evaluation of inclusion/exclusion criteria;

• Randomization;

• Evaluation of echocardiographic parameters: Functional changes of the left ventricle evaluated by TDI; Coronary flow variations assessed by Pulse Wave Velocity; Morphological changes of the left ventricle evaluated RWT;

• Evaluation of diabetic retinopathy by examination of the ocular fundus;

• Administration of the first dose of the experimental or control drug;

• Delivery of the patient's diary;

• Specific anti-tuberculosis prophylaxis for patients with no current disease, but positive results on chest X-ray examination and / or specific tuberculosis diagnosis test (PPD and / or QFT).

***Visit 2 (month 3)***

Visit 2 will be made after 3 months (± 7 days) from the date of visit 1.

During the Visit 2, after verification of the inclusion/exclusion criteria, the detection of adverse events and the assessment of treatment compliance (carried out by examining the Patient's diary), the patient will be submitted to the planned assessments. At the end of the visit, the Patient's Diary will be delivered to the patient. The investigational drug and the control drug will be delivered to the patient, through the hospital pharmacy or according to the procedures established by the procedures in force at the various experimental centers.

The Investigator will invite the Patient to new visit after 3 months (± 7 days) from the date of visit 2 bringing with him the Patient's Diary and he will also instruct the patient to contact him promptly in the eventuality of disturbances of any kind.

Specifically, during the visit 2, the following evaluations will be carried out:

• Verification of the applicable inclusion and exclusion criteria;

• Detection of adverse events;

• Compliance verification through evaluation of the patient's diary;

• Registration of concurrent diseases;

• Registration of concurrent treatments;

• Evaluation of vital signs (arterial pressure, heart rate, body temperature);

• Objective examination;

• BMI;

• Clinical evaluation of RA by using: DAS28; SDAI; count of tender joints (66 joints); counts of swollen joints (68 joints); HAQ;

• Biochemical evaluation of RA by: CRP, ESR;

• A1c evaluation;

• Fasting blood glucose, triglycerides, total cholesterol, HDL cholesterol, LDL cholesterol;

• Hemochromocytometric examination with leukocyte formula and platelet count;

• Complete examination of urine and microalbuminuria;

• Collection of the completed patient diary and delivery of the next diary.

***Visit 3 (month 6)***

Visit 3 will be made after 3 months (± 7 days) from the date of visit 2. During the visit 3, after verification of the inclusion/exclusion criteria, the detection of adverse events and the assessment of treatment compliance, carried out by examining the patient's diary, the patient will be submitted to the planned evaluations. At the end of the visit, the Patient's Diary will be delivered to the Patient. The investigational drug and the control drug will be delivered to the patient, through the hospital pharmacy or according to the procedures established by the procedures in force at the various experimental centers.

The Investigator will invite the Patient to new visit after 6 months (± 7 days) from the date of visit 3, bringing with him the Patient's Diary; he will also instruct the patient to promptly contact him in the eventuality of the occurrence of disorders of any kind.

In particular, during the visit 3, the following evaluations will be carried out:

• Verification of the applicable inclusion and exclusion criteria;

• Detection of adverse events;

• Compliance verification through evaluation of the patient's diary;

• Registration of concurrent diseases;

• Registration of concurrent treatments;

• Evaluation of vital signs (arterial pressure, heart rate, body temperature);

• Objective examination;

• BMI;

• Clinical evaluation of RA by using: DAS28; SDAI; count of tender joints (66 joints); counts of swollen joints (68 joints); HAQ;

• Biochemical evaluation of RA by: CRP, ESR;

• A1c evaluation;

• Fasting blood glucose, triglycerides, total cholesterol, HDL cholesterol, LDL cholesterol;

• Hemochromocytometric examination with leukocyte formula and platelet count;

• Complete examination of urine and microalbuminuria;

• Evaluation of echocardiographic parameters: Functional changes of the left ventricle evaluated by TDI; Coronary flow variations assessed by Pulse Wave Velocity; Morphological changes of the left ventricle evaluated RWT;

• Evaluation of diabetic retinopathy by examination of the ocular fundus;

• Administration of the first dose of the experimental or control drug;

• Delivery of the patient's diary;

• Specific anti-tuberculosis prophylaxis for patients with no current disease, but positive results on chest X-ray examination and / or specific tuberculosis diagnosis test (PPD and / or QFT).

***Visit 4 (month 12)***

Visit 4 will be made after 6 months (± 7 days) from the date of visit 3. During the visit 4, after verification of the inclusion/exclusion criteria, the detection of adverse events and the assessment of treatment compliance, carried out by examining the patient's diary, the Patient will be submitted to the planned evaluations. At the end of the visit, the Patient's Diary will be delivered to the Patient. The investigational drug and the control drug will be delivered to the patient, through the hospital pharmacy or according to the procedures established by the procedures in force at the various experimental centers.

The Investigator will invite the Patient to new visit after 6 months (± 7 days) from the date of visit 3, bringing with him the Patient's Diary; he will also instruct the patient to promptly contact him in the eventuality of the occurrence of disorders of any kind.

Specifically, during the Visit 4, the following evaluations will be carried out:

• Verification of the applicable inclusion and exclusion criteria;

• Detection of adverse events;

• Compliance verification through evaluation of the patient's diary;

• Registration of concurrent diseases;

• Registration of concurrent treatments;

• Evaluation of vital signs (arterial pressure, heart rate, body temperature);

• Objective examination;

• BMI;

• Clinical evaluation of RA by using: DAS28; SDAI; count of tender joints (66 joints); counts of swollen joints (68 joints); HAQ;

• Biochemical evaluation of RA by: CRP, ESR;

• A1c evaluation;

• Fasting blood glucose, triglycerides, total cholesterol, HDL cholesterol, LDL cholesterol;

• Hemochromocytometric examination with leukocyte formula and platelet count;

• Complete examination of urine and microalbuminuria;

• Evaluation of echocardiographic parameters: Functional changes of the left ventricle evaluated by TDI; Coronary flow variations assessed by Pulse Wave Velocity; Morphological changes of the left ventricle evaluated RWT;

• Evaluation of diabetic retinopathy by examination of the ocular fundus;

• Delivery of the patient's diary.

***Visit 5 (Final Visit - month 24)***

Visit 5 will be made after 12 months (± 7 days) from the date of visit 4. During the visit 5, after verification of the inclusion/exclusion criteria, the detection of adverse events and the assessment of treatment compliance, carried out by examining the Patient's diary, the Patient will be submitted to the planned evaluations.

Specifically, during the Visit 5, the following evaluations will be carried out:

• Verification of the applicable inclusion and exclusion criteria;

• Detection of adverse events;

• Compliance verification through evaluation of the patient's diary;

• Registration of concurrent diseases;

• Registration of concurrent treatments;

• Evaluation of vital signs (arterial pressure, heart rate, body temperature);

• Objective examination;

• BMI;

• Clinical evaluation of RA by using: DAS28; SDAI; count of tender joints (66 joints); counts of swollen joints (68 joints); HAQ;

• Biochemical evaluation of RA by: CRP, ESR;

• A1c evaluation;

• Fasting blood glucose, triglycerides, total cholesterol, HDL cholesterol, LDL cholesterol;

• Hemochromocytometric examination with leukocyte formula and platelet count;

• Complete examination of urine and microalbuminuria;

• Evaluation of echocardiographic parameters: Functional changes of the left ventricle evaluated by TDI; Coronary flow variations assessed by Pulse Wave Velocity; Morphological changes of the left ventricle evaluated RWT;

• Evaluation of diabetic retinopathy by examination of the ocular fundus;

• Conclusion of the study.

# ASSESSMENT OF SAFETY AND TOLLERABILITY

During the whole study the experimenters will systematically monitor each subject for the presence of clinical signs and laboratory results indicative of adverse events.

The investigator will evaluate and record the adverse events in detail using the appropriate section of the CRF, and inserting the date and time of onset, the description, the severity, duration and outcome, the relationship with the drug in study, the final diagnosis, if known, and any action taken. On the CRF both the adverse events due to a specific report reported by the health personnel and those spontaneously reported by the subject will be recorded. All side effects will be monitored until they are properly resolved.

## Definitions

### Adverse Event (AE)

### Safety of the treatment will be evaluated during all scheduled visits, and any suspected adverse event (AE) will be recorded and coded according to the Medical Dictionary for Regulatory Activities (MedDRA) system organ class (SOC) classification.

### An adverse event is defined as an unfavorable medical event in a subject, or in a subject undergoing clinical trials, to which a pharmaceutical product has been administered; this event does not necessarily present a causal relationship with the treatment administered. An adverse event can therefore be any negative and unwanted sign (including abnormal laboratory values), symptom or disease that has a temporal association with the administration of an investigational drug, whether it is considered correlated or not with the treatment itself.

### This event may derive either from the use of the drug as foreseen by the protocol or from the printed matter, or from an overdose, accidental or intentional, then from its abuse or suspension. Any worsening of a pre-existing condition or illness is considered an adverse event. Laboratory abnormalities or changes in vital signs are considered to be adverse events only if they result in discontinuation of the study, require medical intervention and / or if the researcher considers them to be adverse events.

### A "treatment-emergent" adverse event is defined as any adverse event with onset or worsening reported by the subject from the time the first dose of study drug is administered until five half-lives have elapsed after discontinuation of dosing.

### A specific scheduled surgery/medical procedure that takes place during the course of the study will not be considered an adverse event if performed for a pre-existing condition and if scheduled prior to entry into the study (prior to the signing of informed consent). However, if the pre-existing condition unexpectedly worsens during the study (for example if the surgery is performed earlier than expected), in this case the worsening of the clinical condition that resulted in the surgery/procedure will be considered an adverse event.

### Serious Adverse Event (SAE)

### Serious AEs (SAEs) are defined as follows:

### a) Patient's death: an event that causes the patient's death.

### b) Danger of life: an event which, in the opinion of the investigator, would have been immediately fatal if medical intervention had not been carried out. This definition does not include an event that would have been fatal if it had taken place in a more severe form.

### c) Hospitalization: an event that determines hospitalization for a period of time of any duration. This definition does not include a visit to the emergency room or an outpatient facility.

### d) Extension of hospitalization: an event that occurs while the subject in the study is admitted to hospital and which prolongs the duration of hospitalization.

### e) Congenital anomaly: an abnormality detected at or after birth, or any anomaly that results in fetal loss.

### f) Persistent or significant disability / incapacity: an event that results in a condition that interferes substantially with the activities of the daily life of a subject participating in a clinical trial. The definition of disability is not intended to include minor medical events, such as headaches, nausea, vomiting, diarrhea, flu, and accidental trauma (for example, sprained ankle).

### g) Important medical event requiring medical or surgical intervention to prevent serious outcomes: an important medical event that may not endanger life or cause death or hospitalization, but which, according to the physician's judgment, may compromise the integrity of the subject or may require medical or surgical intervention to prevent one of the above outcomes (ie, death of the subject, life threatening, hospitalization, prolonged hospitalization, congenital anomaly, persistent or significant disability / incapacity ). Examples of such events are: allergic bronchospasm requiring intensive treatment in the emergency room or at home; blood dyscrasia; convulsions that do not involve the patient's admission; development of drug dependence or abuse of the same.

### h) Transmission of infectious agents by the study drug.

### i) Miscarriage: spontaneous abortion by the subject participating in the study.

### j) Elective abortion: elective abortion to which the subject participating in the study is subjected.

### If an adverse event meets one of the above criteria, it must be reported to the security officer of the security organization of the clinical research organization (CRO) as an SAE according to the procedures described in paragraph 7.5 (within 24 hours of the occurrence of the event or notification by the experimental center).

### Adverse Drug Reaction (ADR)

### As for the medicinal products already on the market, such as those used in this study, an adverse drug reaction (ADR) is a drug response that is harmful and unwanted.

### Suspected Unexpected Serious Adverse Reaction (SUSAR)

For unexpected adverse drug reaction (Suspected Unexpected Serious Adverse Reaction (SUSAR) means an ADR whose type or intensity does not match the existing information on the product. In the present study, the drugs are administered according to the indication and dosage required by the Summary of Product Characteristics; therefore, the reference document concerning the existing information on the product is represented by the RCP itself, which replaces the Investigator's Brochure.

## Severity of AEs

## The Investigator will use the following definitions to determine the degree of severity of AEs:

## *Mild*: the adverse event is transient and well tolerated by the subject and does not affect the normal daily activities of the subject.

## *Moderate*: the adverse event causes a discomfort to the subject and determines the interruption of normal activities

## *Severe*: the adverse event causes considerable interference with the normal activity of the subject and can determine the inability to perform normal activities or endanger their life.

## 7.3 Relationship with study drug

The investigator will use the causality categories defined by WHO to evaluate the relationship between adverse event and study drug administration; the categories are as follows:

| **Term of causality** | **Evaluation Criteria** |
| --- | --- |
| **Certain** | • Event or alteration of a laboratory parameter with a plausible temporal relationship with drug intake.  • It cannot be explained by the disease for which the drug is used or by concomitant medication.  • Plausible response (pharmacologically or clinically) to drug withdrawal. ("De-challenge")  • Pharmacologically or clinically well-defined event (eg an objective and specific pathology, or a pharmacologically recognized phenomenon)  • A satisfactory response to the possible re-administration of the drug ("re challenge"). |
| **Probable** | • Event or alteration of a laboratory parameter with a reasonable temporal relationship with drug intake.  • It is unlikely to be attributable to the disease for which the drug is used or to concomitant medications.  • Reasonable clinical response to drug withdrawal. ("Dechallenge")  • Re-administration of the drug ("re-challenge") is not required. |
| **Possible** | • Event or alteration of a laboratory parameter with a reasonable temporal relationship with drug intake.  • It could also be explained by the disease or by concomitant medication.  • Information on discontinuation of medication may be insufficient or unclear. |
| **Improbable** | • Event or alteration of a laboratory parameter with a temporal relationship with the intake of the drug that makes the relationship unlikely (but not impossible).  • The disease or concomitant medications provide a plausible explanation. |
| **Unrelated** | • Event or alteration of a laboratory parameter for which the temporal relationship with drug administration excludes the causal relationship.  • The clinical event is reasonably determined by other drugs, chemicals or concomitant diseases. |

## Registration of AEs

The SAEs must be registered from the moment the patient signs the informed consent until 5 half-lives have passed after the interruption of the administration of the drug, whether these events were collected during telephone contact and / or scheduled visits, or that they they have been spontaneously reported by the patient.

All non-serious AEs occurring from the time the study drug is initiated will be collected, until 5 half-lives have elapsed after discontinuation of the drug, and whether these events were collected during telephone contact and / or scheduled visits, whether they have been spontaneously reported by the patient.

Episodes of hypoglycemia will be recorded in a specific field of CRF.

## Reporting of AEs

The methods for reporting AEs are as follows:

SUSAR with lethal outcome / life-threatening

The Investigator will immediately communicate, or within 24 hours, from the moment in which it becomes aware, to the Sponsor and to the security manager of the security unit of CRO Pharmaceutical Development and Services srl the SUSARs that have had a lethal outcome or that put into life danger of the subjects; the contact details to be used for communication are as follows:

Sponsor – Università of L’Aquila

Prof. Roberto Giacomelli

[roberto.giacomelli@cc.univaq.it](mailto:roberto.giacomelli@cc.univaq.it)

Tel: 0862433395

PharmaD&S

Dr. Irene Barneschi

[ibarneschi@pharmades.it](mailto:ibarneschi@pharmades.it )

Tel: 055-7224179

The Investigator shall record the aforesaid events in the Pharmacovigilance (RNF) Nation Network according to the methods and times envisaged by the pharmacovigilance legislation currently in force.

### Severe AE (SAE)

With the exception of the SUSARs referred to in the previous paragraph, each SAE, both related and not related to the drug, must be communicated by the Investigator to the Sponsor and to the safety manager of the CRO Pharmaceutical Development and Services safety unit. within 3 days from the moment in which the SAE occurred or from the moment the Investigator became aware of it; the contact details to be used for communication are as follows:

Sponsor – Università of L’Aquila

Prof. Roberto Giacomelli MD-PhD

[roberto.giacomelli@cc.univaq.it](mailto:roberto.giacomelli@cc.univaq.it)

Tel: 0862433395

PharmaD&S

Dr. Irene Barneschi

ibarneschi@pharmades.it

Tel: 055-7224179

The Investigator provides for the registration of the SAEs in the RNF, according to the procedures and timescales envisaged by the pharmacovigilance legislation currently in force.

**7.6 Pregnancy**

Patients who remain pregnant during the study will be discontinued administration of the drug.

Spontaneous or induced abortion is considered an SAE and must be communicated within 24 hours from the time the investigator became aware of it.

# STATISTICAL ANALYSIS

## Sample Size

This clinical study was designed to demonstrate the superiority of anakinra compared to treatment with an anti-TNF-α drug in terms of efficacy in improving glycemic control as well as in reducing the signs and symptoms of rheumatoid arthritis in patients with rheumatoid arthritis and Type 2 diabetes as a comorbidity. The demonstration of the superiority of anakinra is based on the determination of a mean difference of 0.25 percentage points of glycated hemoglobin between the two treatment groups (Larsen, 2007).

It has been estimated that by assuming:

- Type I error: α = 0.05
- Power: 1-β = 0.90
- Difference to the numerator of: 0.0625 (glycated hemoglobin difference expected between treatments)
- Standard deviation: 0.5


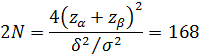


A sample of 168 patients, 84 per treatment group, will be evaluated to calculate the superiority of the study drug with respect to the control drug.

Considering a 10% withdrawal rate, as well as an inflation factor of 1.01 deriving from the use of O'Brian-Fleming's stopping boundaries (see 8.6), the total sample have to be at least 200, ie 100 subjects per group of treatment.

## Statistical Model

### Statistical analysis

Statistical analysis will compare the effects of treatment in the two groups, adjusting covariance as sex and age, using mixed linear model


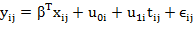


which will address the longitudinal character of the study design.

## Randomization

## Patients are assigned to each treatment group using the method of randomized block randomization to avoid possible imbalances.

## When the patient enters the study, he is assigned to one of the two treatment groups defined as A and B. Within each block, the order of treatment is randomized and the size of the group remains unknown to the physician, to avoid any distortion in the assignment. For example, let's assume a block of dimension 4, with two A and two B.

For each group of 4 there are
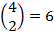
 possible combinations to assign the treatment, i.e:

{(A A B B); (A B A B); (A B B A); (B A A B); (B A B A); (B B A A)}.

The randomization scheme chooses one of these six possibilities with equal probability (ie 1/6) and proceeds as follows. The first four patients entering the clinical study are assigned to treatment A and B according to the chosen permutation. For the next block of 4, another combination of treatment is chosen randomly between the six permutations above and the next four patients are assigned to treatments A or B according to the model. This process continues until the end of the study. The advantage, using this scheme, is that the difference in the number of patients receiving A against B cannot exceed 2, regardless of when the study ends.

## Level of significance

For the type I error, a significance level of 0.05 is assumed.

## Management of missing data

The non-compliance with the treatment assigned by randomization will be treated using an intent-to-treat analytic model: the comparison between treatments is based on the difference between the average responses among randomized groups, ignoring the fact that some patients are non-compliant.

**8.6. Interim analysis**

In order to confirm the possible superiority of the study drug, on the bases of intermediate data or, alternatively, the confirmed non-existence of a trend of interest, an *ad interim analysis* is planned after 12 months from the beginning of the recruitment by group-sequential methods using O'Brian-Fleming's stopping boundaries. The sample size of the trial takes into account the inflation factor deriving from the use of the thresholds.

# ACCESS TO THE ORIGINAL DOCUMENTS

The Investigator must allow the Regulatory Authorities and the personnel designated by the Ethics Committee or the Investigator/Sponsor direct access to all the original documentation of the study, including the Informed Consent forms signed by the subjects included in the study and the hospital records and/or outpatient records. Those who have direct access to such documentation must take all reasonable precautions to keep the identity of the subjects and the confidential information owned by the Investigator/Sponsor confidential, in compliance with the applicable regulatory provisions.

# MANAGEMENT OF CLINICAL DATA

Only the Investigator/Sponsor is the owner of all data and results of the study.

Data management and registration will be entrusted to CRO Pharmaceutical Development and Services srl.

# MONITORING PROCEDURES AND QUALITY ASSESSEMENT

## Data Collection Sheet (CRF)

Data will be collected by electronic data collection (eCRF). The PheedIt program will be used for data collection and management. Pheedit software is compliant with current legislation (Eudralex Vol.4 GMP_Annex 11).

## Monitoring procedures

The CRO, through specific recruited personnel, before the beginning of the study will illustrate to the Investigator, and to any other personnel of the staff involved, the protocol, the CRF, and all the operational aspects related to the study.

The study will be monitored by CRO Pharma D & S. The monitor will review the relevant original documents in accordance with the Standard Operating Procedures (SOP) and with the rules of Good Clinical Practice (GCP), as applicable to "no-profit" studies. The original documents include the reports of the instrumental and laboratory tests requested by the study, and any other medical documentation necessary to confirm the information contained in the data sheets.

The Investigator must be available for monitoring visits whenever the Sponsor deems it necessary. The Investigator and his staff will have to cooperate with the Monitor during the monitoring procedures and provide all the necessary information.

All monitoring activities will be reported on appropriate modules, which will be archived in the Trial Master File.

## Audit and Inspections

The Investigator and the Sponsor must allow the competent Institutions and the Regulatory Authorities to carry out the audits and inspections, as an integral part of the quality assurance system.

In any case, data privacy will be maintained. All audits and inspections will then be documented and kept in the study file.

# ETHICAL FEATURES

## Ethic authorizations

The study will be started only after the ethical opinion issued by the Ethics Committee and the authorization issued by the Competent Authority.

Before the study could begin, the Investigator must have received a copy of the approval and authorization document.

For each substantive amendment protocol the approval of the Ethics Committee will be required.

## Informed consent

Before the start of the study, the "Written Information for the Patient" to be provided to the subjects and the Informed Consent form(s) must be submitted for examination and approval by the local Ethical Committee, together with the protocol.

The Informed Consent must be requested, obtained and documented by the Investigator in compliance with the applicable regulatory provisions, the GCP and the ethical principles deriving from the Helsinki Declaration.

The guarantees relating to the protection and protection of personal data pursuant to Legislative Decree 30.6.03 n. 196 ("privacy code") will be provided in "Written Information for the Patient" or in accordance with the procedures and forms in force at the individual experimental centres.

The Investigator is responsible for obtaining informed consent from patients or their legal representatives before enrolment in the study; therefore, before to screening procedures, he will inform patients and/or their legal representatives in a comprehensible language about the type of study, the purpose, the risks and benefits deriving from it and will answer all the questions concerning the study. Once the necessary information has been obtained, the informed consent form will be signed and dated by the patient and / or legal representative and the Investigator. A copy of these will be given to the subject or legal representative, while the originals will be included in the study documentation and will be kept by the Investigator for prescribed time.

## Good Clinical Practice

All the parties involved in the study agree and verify that this experimentation is conducted in accordance with the ethical principles, which derive their origin from the Helsinki Declaration (Appendix I), to the principles of Good Clinical Practice (Ministerial Decree No. 162 of 15/07/ 97, as applicable to "no-profit" studies) and to the applicable regulatory provisions.

The study will be conducted taking into account regulatory requirements and legal obligations.

# ADMINISTRATIVE SETTLEMENT

## Storage

The Investigator/Sponsor must provide for the conservation of the essential documents of the study as specified by the principles of the GCP and in accordance with the applicable regulatory provisions. The Investigator/Sponsor must take the necessary measures to prevent accidental or premature destruction.

The Investigator/Institution must keep the essential documents for at least 7 years from the end of the study in accordance with Legislative Decree 200/2007, art. 18, paragraph 1. However, these documents must be kept for longer periods if required by the applicable regulatory provisions.

## Privacy

The data collected during the study will be processed in compliance with the law relating to the protection of personal data (Legislative Decree 30.6.03 No. 196, "Privacy Code") and subsequent amendments and additions.

The data indicated will be collected by the study physician, anonymously, exclusively according to the realization of the study and participation in it. In the Data Collection Sheet (CRF), only the patient's number and his initials must appear and if the patient's name appears on any document (eg laboratory tests or other reports) the name must be deleted before the copy of the document be delivered to the Investigator/Sponsor.

The investigator must keep a list of patients so that their identification is possible.

## Insurance

The study is covered by the specific insurance policy of the HDI-Gerling insurance company.

# RESPONSABILITY OF THE INVESTIGATOR

The Investigator is required to conduct the study in accordance with the study protocol, in accordance with the principles of Good Clinical Practice (DM No. 162 of 15/07/97 and subsequent additions), with the principles of the Helsinki Declaration (Appendix I) and in accordance with applicable legislation.

The Investigator must ensure that all personnel involved in the study are adequately informed about the protocol, study treatment and controls to be performed. The Investigator will maintain a list of qualified personnel who have been assigned certain tasks related to the study.

The Investigator is aware of being responsible for all actions delegated by him to the other members of his staff designated to conduct the study.

# PUBBLICATION OF THE DATA

The study was included in the EudraCT database, as required by current legislation. The results of the study will be published in International Journals with impact factor. The name and order of the authors will be agreed between the Sponsor and the Investigators.

# REFERENCES

1. *McInnes IB, Schett G.* [*The pathogenesis of rheumatoid arthritis.*](http://www.ncbi.nlm.nih.gov/pubmed/22150039) *N Engl J Med. 2011 Dec 8;365(23):2205-19.*
2. *Avina-Zubieta JA, Thomas J, Sadatsafavi M, Lehman AJ, Lacaille D.* [*Risk of incident cardiovascular events in patients with rheumatoid arthritis: a meta-analysis of observational studies.*](http://www.ncbi.nlm.nih.gov/pubmed/22425941) *Ann Rheum Dis. 2012 Sep;71(9):1524-9.*
3. *Wasko MC, Kay J, Hsia EC, Rahman MU.* [*Diabetes mellitus and insulin resistance in patients with rheumatoid arthritis: risk reduction in a chronic inflammatory disease.*](http://www.ncbi.nlm.nih.gov/pubmed/21452264) *Arthritis Care Res (Hoboken). 2011 Apr;63(4):512-21)*
4. *Gonzalez-Gay MA, De Matias JM, Gonzalez-Juanatey C, Garcia-Porrua C, Sanchez-Andrade A, Martin J, Llorca J.* [*Anti-tumor necrosis factor-alpha blockade improves insulin resistance in patients with rheumatoid arthritis.*](http://www.ncbi.nlm.nih.gov/pubmed/16539824) *Clin Exp Rheumatol. 2006 Jan-Feb;24(1):83-6*
5. *Yudkin JS, Kumari M, Humphries SE, Mohamed-Ali V.* [*Inflammation, obesity, stress and coronary heart disease: is interleukin-6 the link?*](http://www.ncbi.nlm.nih.gov/pubmed/10657556) *Atherosclerosis 2000 Feb;148(2):209-14.*
6. *Larsen CM, Faulenbach M, Vaag A, Vølund A, Ehses JA, Seifert B, Mandrup-Poulsen T, Donath MY. Interleukin-1-receptor antagonist in type 2 diabetes mellitus. N Engl J Med. 2007 Apr 12;356(15):1517-26*
7. *Mosca A, et al, Recommendations for the implementation of the International standardization of glycated hemoglobin measurementin Italy G It Diabetol Metab 2009;29:184-188*
