# Supplementary material for: Anti-interleukin-1 treatment in patients with rheumatoid arthritis and type 2 diabetes (TRACK): A multicentre, open-label, randomised controlled trial
Source: PLoS Med. 2019 Sep 12;16(9):e1002901. doi: 10.1371/journal.pmed.1002901 (PMC6742232; doi:10.1371/journal.pmed.1002901)
Supplement: S2 Text — (DOC) [file pmed.1002901.s004.doc]

**STUDIO NO-PROFIT**

**(Decreto Ministero della Salute 17 dicembre 2004)**

**Titolo dello Studio**

**STUDIO CLINICO “NO-PROFIT” PER IL MIGLIORAMENTO DELLA PRATICA CLINICA, PER VALUTARE L’EFFICACIA DI ANAKINRA NELLA RIDUZIONE DELL’EMOGLOBINA GLICATA IN SOGGETTI CON ARTRITE REUMATOIDE E DIABETE; STUDIO CLINICO RANDOMIZZATO, CONTROLLATO, IN APERTO, PER GRUPPI PARALLELI.**

*Sponsor e Sperimentatore Principale****:***

Prof. Roberto Giacomelli

Cattedra di Reumatologia

Università degli Studi di L’Aquila

Unità Operativa complessa di Reumatologia

Ospedale San Salvatore

L’Aquila

### Prodotto/codice: Anakinra (Kineret®)

### Protocollo n: ANAKIN-RA-DIABETES

### Versione: Finale

### Data: 15.11.12

**PAGINA DELLE FIRME DI APPROVAZIONE**

Io sottoscritto dichiaro di aver preso visione e di approvare il presente protocollo dal titolo: “Studio clinico “no-profit”per il miglioramento della pratica clinica, per valutare l’efficacia di anakinra nella riduzione dell’emoglobina glicata in soggetti con artrite reumatoide e diabete; studio clinico randomizzato, controllato, in aperto, per gruppi paralleli”.

*Sponsor e Sperimentatore Principale****:***

Prof. Roberto Giacomelli

Cattedra di Reumatologia

Università degli Studi di L’Aquila

Unità Operativa complessa di Reumatologia

Ospedale San Salvatore

L’Aquila.

____________, ­­­________

Luogo e data

____________________________________________________________

Firma Prof. Giacomelli

**SINOSSI**

| **Titolo dello studio** | Studio clinico “no-profit”per il miglioramento della pratica clinica, per valutare l’efficacia di anakinra nella riduzione dell’emoglobina glicata in soggetti con artrite reumatoide e diabete; studio clinico randomizzato, controllato, in aperto, per gruppi paralleli. |
| --- | --- |
| **Numero protocollo/data** | Anakin-Ra-Diabetes / 15.11.12 |
| **Prodotto in studio** | Anakinra (Kineret®) |
| **Numero di Centri/Paesi** | 25 centri/Italia |
| **Periodo di studio** | L’invio della richiesta di parere etico/autorizzazione è prevista per il mese di dicembre 2012 |
| **Sperimentatore Principale** | Prof. Roberto Giacomelli  Cattedra di Reumatologia, Università degli Studi di L’Aquila  Unità Operativa complessa di Reumatologia, Ospedale San Salvatore. L’Aquila. |
| **Obiettivo dello studio** | Verificare se la somministrazione di anakinra in pazienti affetti da artrite reumatoide (AR) e da diabete di tipo 2 (DM2) come comorbidità è in grado determinare un miglioramento del controllo glicemico, oltre che dei segni e sintomi dell’AR. |
| **Disegno di studio** | Studio clinico randomizzato, controllato, per gruppi paralleli, in aperto, multicentrico.  Studio no-profit (Decreto 17 dicembre 2004). |
| **Numero di pazienti pianificati** | Un totale di 200 pazienti con diagnosi di AR e DM2 saranno arruolati in circa 20 centri in Italia, per ottenere almeno 168 pazienti valutabili (84 per gruppo di trattamento). |
| **Popolazione in studio** | **Criteri di inclusione:**   - pazienti maschi e femmine di età ≥ 18 anni; - precedente diagnosi di AR che soddisfi i criteri dell’American College or Rheumatology (ACR)/ E[uropean League Against Rheumatism](http://www.google.it/url?sa=t&rct=j&q=&esrc=s&source=web&cd=1&ved=0CFcQFjAA&url=http%3A%2F%2Fwww.eular.org%2F&ei=6jj1T7vwLcOE4gSI5O2ABw&usg=AFQjCNGmPXvJMz-hWUB05QV0iKnzlX5b3g&sig2=EcvaoARgAK5nz6BVdL8hQQ) (EULAR); - DM2 diagnosticato da almeno 6 mesi secondo i criteri dell’American Diabetes Association (ADA); - pazienti con risposta inadeguata ad un precedente trattamento con metotrexato (determinata sulla base della valutazione del medico); - BMI<35 - emoglobina glicata > 7% < 10% - punteggio minimo Disease Activity Score-28 (DAS 28) > 3.2 - per i pazienti trattati precedentemente con un farmaco biologico, sospensione del trattamento per un periodo di almeno un 1 mese prima dell’arruolamento; - per i pazienti in trattamento farmacologico con farmaci ipoglicemizzanti al momento dell’arruolamento, nessuna variazione in termini di farmaco somministrato e di posologia, per un periodo di almeno 3 mesi prima dell’arruolamento; - per i pazienti in trattamento farmacologico con altri farmaci al momento dell’arruolamento, nessuna variazione in termini di farmaco somministrato e di posologia per un periodo di almeno un 1 mese prima dell’arruolamento; - assenza di segni e sintomi riferibili a cardiopatia ischemica - firma del Consenso informato.   **Criteri d’esclusione**   - DM2 diagnosticato da più di 10 anni; - Infezione acuta in atto o infezione cronica grave in fase di riacutizzazione, che presenti uno o più dei seguenti elementi:   - livelli di proteina C reattiva maggiori > 30 mg/L  - febbre  - trattamento in corso con antibiotici;   - Infezioni granulomatose croniche (ad esempio presenza di tubercolosi diagnosticata su base radiografica o mediante specifici esami di laboratorio) - storia clinica di infezioni ricorrenti o presenza di patologie che predispongano a contrarre infezioni; - valori di peptide C < 0.5 ng/mL (0.1665 nmol/L) - presenza di neutropenia (leucociti < 2000/mm^3^ ) o anemia (emoglobina < 11g/dl per l’uomo e 10g/dl per la donna); - presenza di una o più delle Controindicazioni previste dal RCP del farmaco in studio (anakinra): - presenza di una o più delle Controindicazioni previste dal RCP del farmaco di controllo (inibitori del TNF-alfa; ATC L04AB) - presenza di una o più controindicazioni al trattamento con metotrexato; - precedente attacco ischemico o infarto del miocardio; - insufficienza cardiaca di classe NYHA III o IV; - insufficienza epatica o malattia epatica in atto (valori di ALAT/ASAT aumentati di almeno due volte rispetto ai limiti di normalità); - gravidanza in atto o donna in età fertile con non utilizza misure contraccettive; - allattamento; - partecipazione ad un altro studio clinico fino a 6 mesi prima la randomizzazione; - sindrome depressiva grave o altra malattia psichiatrica che, a giudizio del medico, possa precludere la partecipazione del paziente allo studio; - presenza di neoplasie maligne note; - storia clinicamente significativa di abuso di alcolici o di tossicodipendenza che, a giudizio del medico, possa precludere la partecipazione del soggetto allo studio; - qualsiasi condizione che, a giudizio del medico, precluda la possibilità di utilizzare il farmaco in studio (anakinra; Kineret) o il farmaco di controllo (inibitori del TNF-alfa; ATC L04AB) secondo le condizioni previste dal RCP; - qualsiasi altra condizione o parametro di laboratorio che, a giudizio del medico, precluda la partecipazione del soggetto allo studio. |
| **Trattamenti** | I pazienti saranno randomizzati in due gruppi di trattamento.  Gruppo in trattamento col farmaco sperimentale:  I soggetti verranno trattati con anakinra (Kineret®) 100 mg/UID per via sottocutanea; in conformità a quanto previsto dal RCP.  Il trattamento avrà la durata di 24 mesi.  Gruppo trattato con il farmaco di controllo:  I soggetti verranno trattati con un farmaco inibitore del TNF-alfa (ATC L04AB); la scelta dello specifico prodotto medicinale è lasciata al giudizio del medico. La posologia e le modalità di somministrazione dovranno essere conformi a quanto previsto dal RCP del prodotto medicinale somministrato.  Il trattamento avrà la durata di 24 mesi.  Trattamenti concomitanti  *Metotrexato*  Ai soggetti verrà somministrato metotrexato ad un dosaggio compreso tra 15 e 20 mg alla settimana (a giudizio del medico), in conformità a quanto previsto dal RCP di questa classe di farmaci.  *Trattamento ipoglicemizzante*  I soggetti saranno invitati a mantenere le abitudini alimentari e lo stile di vita in corso al momento della randomizzazione.  Al momento dell’arruolamento, i soggetti manterranno il trattamento farmacologico ipoglicemizzante in corso prima dello studio. Nel corso dello studio, il trattamento ipoglicemizzante potrà essere modificato a giudizio del medico secondo linee guida; (Nathan DM, Buse JB, Davidson MB, et al. Medical management of hyperglycemia in type 2 diabetes: a consensus algorithm for the initiation and adjustment of therapy: a consensus statement of the American Diabetes Association and the European Association for the Study of Diabetes. Diabetes Care. 2009;32(1):193-203).  *Trattamento con farmaci antiinfiammatori*  Al momento dell’arruolamento, i soggetti manterranno il trattamento con farmaci antiinfiammatori steroidei e non-steroidei (NSAID) in corso prima dello studio. Nel corso dello studio, il trattamento con NSAID potrà essere modificato a giudizio del medico; è prevista l’interruzione dello studio in caso di variazione del dosaggio dei farmaci antiinfiammatori steroidei.  *Altri trattamenti*  Per tutta la durata dello studio tutti i soggetti riceveranno 5 mg di acido folico ogni settimana. |
| **Criteri di valutazione** | **Endpoint Primario**  Variazione del livello di emoglobina glicata (HbA_1c_), rispetto alle condizioni basali, dopo 3 mesi, 6 mesi, 12 mesi e 24 mesi di trattamento.  I risultati di HbA_1c_ devono essere espressi da tutti i laboratori con l’unità di misura del SI (Sistema Internazionale; mmol/mol – senza decimale) e con unità derivate NGSP (% - in decimale), usando l’equazione generale IFCC-NGSP (unità DCCT).  **Endpoint Secondari**  *Efficacia*  Segni e sintomi dell’AR:  Variazione, rispetto alle condizioni basali, dopo 3 mesi, 6 mesi, 12 mesi e 24 mesi di trattamento dei seguenti parametri di laboratorio:   - Percentuale dei pazienti in remissione (criteri E[uropean League Against Rheumatism](http://www.google.it/url?sa=t&rct=j&q=&esrc=s&source=web&cd=1&ved=0CFcQFjAA&url=http%3A%2F%2Fwww.eular.org%2F&ei=6jj1T7vwLcOE4gSI5O2ABw&usg=AFQjCNGmPXvJMz-hWUB05QV0iKnzlX5b3g&sig2=EcvaoARgAK5nz6BVdL8hQQ) - EULAR) in base alla valutazione del Disability Activity Score (DAS28) e del Simplified Disease Activity Index (SDAI); - Percentuale dei pazienti migliorati (criteri EULAR) in base alla valutazione del DAS28 e dello SDAI; - Conta delle articolazioni dolenti (66 articolazioni); - Conta delle articolazioni tumefatte (68 articolazioni); - Somministrazione dell’Health Assessment Questionnaire (HAQ).   Parametri di laboratorio  Variazione, rispetto alle condizioni basali, dopo 3 mesi, 6 mesi, 12 mesi e 24 mesi di trattamento dei seguenti parametri di laboratorio:   - Proteina C reattiva (CRP) e velocità di eritrosedimentazione (ESR); - Livelli plasmatici di glucosio; - Livelli plasmatici di trigliceridi, colesterolo totale, colesterolo HDL, colesterolo LDL; - Microalbuminuria.   Variazione, rispetto alle condizioni basali, dopo 6 mesi, 12 mesi di trattamento dei seguenti parametri di laboratorio:   - anticorpi anti-DNA a doppia elica (anti-dsDNAs) e anticorpi anti-nucleo (ANAs).   Variazione, rispetto alle condizioni basali e dopo 12 mesi di trattamento dei seguenti parametri di laboratorio:   - Anticorpi anti-citrullina (anti-CCP) e fattore reumatoide.   Parametri strumentali   - Variazione dei parametri ecocardiografici:   Valutazione, rispetto al basale, dopo 6 mesi, 12 mesi e 24 mesi di trattamento dei seguenti parametri:   - Variazioni funzionali del ventricolo sinistro valutate mediante TDI (Tissue Doppler Imaging); - Variazioni del flusso coronarico valutate mediante Pulse Wave Velocity; - Cambiamenti morfologici del ventricolo sinistro valutati mediante Relative Wall Thickness (RWT)   Altri parametri clinici   - Valutazione della retinopatia diabetica mediante esame del fondo oculare (criteri AMD et al, 2002); - Indice di massa corporea (BMI)   *Sicurezza*   - Valutazione della frequenza e dell’intensità degli eventi avversi (AE) secondo le procedure definite dal protocollo di studio. - Valutazione dei parametri di laboratorio e strumentali - Valutazione dei segni vitali e dell’esame obiettivo. - Valutazione degli episodi di ipoglicemia |
| **Analisi statistica** | L’analisi statistica effettuerà un confronto tra gli effetti del trattamento nei due gruppi, aggiustando la covarianza come sesso e età, per mezzo di modelli lineari misti conformi al carattere longitudinale del disegno dello studio. |

**INDICE**

[**1.** **INTRODUZIONE 10**](#__RefHeading___Toc340824305)

[1.1 Informazioni di base e razionale 10](#__RefHeading___Toc340824306)

[1.2 Descrizione del farmaco 12](#__RefHeading___Toc340824307)

[1.3 Valutazione Rischio/Beneficio 12](#__RefHeading___Toc340824308)

[2. OBIETTIVI DELLO STUDIO 12](#__RefHeading___Toc340824309)

[3. DISEGNO SPERIMENTALE 12](#__RefHeading___Toc340824310)

[3.1 Endpoint dello Studio 12](#__RefHeading___Toc340824311)

[3.2 Disegno dello studio 14](#__RefHeading___Toc340824312)

[3.3 Randomizzazione 14](#__RefHeading___Toc340824313)

[4. POPOLAZIONE IN STUDIO 15](#__RefHeading___Toc340824314)

[4.1 Dimensione del campione 15](#__RefHeading___Toc340824315)

[4.2 Selezione dei pazienti 15](#__RefHeading___Toc340824316)

[4.2.1 Criteri di inclusione 15](#__RefHeading___Toc340824317)

[4.2.2 Criteri di esclusione 15](#__RefHeading___Toc340824318)

[4.3 Criteri per il ritiro dei soggetti dalla sperimentazione 17](#__RefHeading___Toc340824319)

[5. TRATTAMENTO DEI SOGGETTI 18](#__RefHeading___Toc340824320)

[5.1 Trattamento 18](#__RefHeading___Toc340824321)

[5.1.1 Dosaggio, Posologia e via di somministrazione 18](#__RefHeading___Toc340824322)

[5.1.2 Trattamenti concomitanti 19](#__RefHeading___Toc340824323)

[5.1.3 Altri trattamenti 20](#__RefHeading___Toc340824324)

[5.1.4 Speciali avvertenze e precauzioni d’uso 20](#__RefHeading___Toc340824325)

[5.1.5 Confezionamento, etichettatura del farmaco 20](#__RefHeading___Toc340824326)

[5.1.6 Conservazione del farmaco 20](#__RefHeading___Toc340824327)

[5.1.7 Distribuzione del farmaco ai pazienti 20](#__RefHeading___Toc340824328)

[5.1.8 Drug Accountability e restituzione dei materiali 21](#__RefHeading___Toc340824329)

[5.2 Compliance 21](#__RefHeading___Toc340824330)

[5.3 Durata del trattamento 21](#__RefHeading___Toc340824331)

[5.4 Sospensione/Interruzione del trattamento 21](#__RefHeading___Toc340824332)

[6. PROCEDURE DELLO STUDIO 22](#__RefHeading___Toc340824333)

[6.1 Valutazione endpoint primario 22](#__RefHeading___Toc340824334)

[6.2 Valutazione degli endpoint secondari 22](#__RefHeading___Toc340824335)

[6.2.1 DAS 28 22](#__RefHeading___Toc340824336)

[6.2.2 SDAI 23](#__RefHeading___Toc340824337)

[6.2.3 Conta delle Articolazioni tumefatte 23](#__RefHeading___Toc340824338)

[6.2.4 Conta delle Articolazioni dolenti 23](#__RefHeading___Toc340824339)

[6.2.5 VAS paziente 24](#__RefHeading___Toc340824340)

[6.2.6 VAS Medico 24](#__RefHeading___Toc340824341)

[6.2.7 VAS Dolore 24](#__RefHeading___Toc340824342)

[6.2.8 Questionario per la valutazione della salute (HAQ) 24](#__RefHeading___Toc340824343)

[6.2.9 Parametri di laboratorio per la valutazione dell’AR: 24](#__RefHeading___Toc340824344)

[6.2.10 Parametri di laboratorio per la valutazione del DM: 25](#__RefHeading___Toc340824345)

[6.2.11 Parametri strumentali ecocardiografici: 25](#__RefHeading___Toc340824346)

[6.2.12 Valutazione della retinopatia diabetica 25](#__RefHeading___Toc340824347)

[6.2.13 Indice di massa corporea (BMI) 26](#__RefHeading___Toc340824348)

[6.2.14 Endpoint di Safety 26](#__RefHeading___Toc340824349)

[6.3 Esami di laboratorio generali 26](#__RefHeading___Toc340824350)

[6.3.1 Ematologia 26](#__RefHeading___Toc340824351)

[Esame emocromocitometrico con formula leucocitaria e conta piastrinica. 26](#__RefHeading___Toc340824352)

[6.3.2 Chimica clinica 26](#__RefHeading___Toc340824353)

[6.3.3 Analisi delle urine: 26](#__RefHeading___Toc340824354)

[6.4 Calendario delle visite 27](#__RefHeading___Toc340824355)

[7. VALUTAZIONE DELLA SICUREZZA E TOLLERABILITA’ 34](#__RefHeading___Toc340824356)

[7.1 Definizioni 35](#__RefHeading___Toc340824357)

[7.1.1 Evento avverso (AE) 35](#__RefHeading___Toc340824358)

[7.1.2 Evento avverso serio (SAE) 36](#__RefHeading___Toc340824359)

[7.1.3 Reazione avversa da farmaco (ADR) 37](#__RefHeading___Toc340824360)

[7.1.4 Reazione avversa da farmaco inattesa (SUSAR) 37](#__RefHeading___Toc340824361)

[7.2 Intensità degli eventi avversi 37](#__RefHeading___Toc340824362)

[7.3 Relazione con il farmaco in studio 38](#__RefHeading___Toc340824363)

[7.4 Registrazione degli eventi avversi 38](#__RefHeading___Toc340824364)

[7.5 Segnalazione degli eventi avversi 39](#__RefHeading___Toc340824365)

[7.5.1 SUSAR con esito letale/pericolo di vita 39](#__RefHeading___Toc340824366)

[7.5.2 Evento Avverso Serio (SAE) 40](#__RefHeading___Toc340824367)

[8. ANALISI STATISTICA 41](#__RefHeading___Toc340824368)

[8.1 Calcolo della dimensione del campione 41](#__RefHeading___Toc340824369)

[8.2 Modello statistico 41](#__RefHeading___Toc340824370)

[8.2.1 Analisi statistica 41](#__RefHeading___Toc340824371)

[8.3 Procedure di randomizzazione 42](#__RefHeading___Toc340824372)

[8.4 Livello di significatività 42](#__RefHeading___Toc340824373)

[8.5 Gestione dei dati mancanti 42](#__RefHeading___Toc340824374)

[9. ACCESSO DIRETTO AI DOCUMENTI ORIGINALI 43](#__RefHeading___Toc340824375)

[10. GESTIONE DEI DATI CLINICI 43](#__RefHeading___Toc340824376)

[11. PROCEDURE DI CONTROLLO E DI ASSICURAZIONE DELLA QUALITA’ 43](#__RefHeading___Toc340824377)

[11.1 Scheda Raccolta Dati (CRF) 43](#__RefHeading___Toc340824378)

[11.2 Monitoraggio 43](#__RefHeading___Toc340824379)

[11.3 Audit e Ispezioni 44](#__RefHeading___Toc340824380)

[12. ASPETTI ETICI 44](#__RefHeading___Toc340824381)

[12.1 Autorizzazioni Etiche 44](#__RefHeading___Toc340824382)

[12.2 Consenso Informato 44](#__RefHeading___Toc340824383)

[12.3 Good Clinical Pratice 45](#__RefHeading___Toc340824384)

[13. PROCEDURE AMMINISTRATIVE 45](#__RefHeading___Toc340824385)

[13.1 Archiviazione 45](#__RefHeading___Toc340824386)

[13.2 Confidenzialità delle informazioni 46](#__RefHeading___Toc340824387)

[13.3 Copertura assicurativa 46](#__RefHeading___Toc340824388)

[14. RESPONSABILITA’ DELLO SPERIMENTATORE 46](#__RefHeading___Toc340824389)

[15. PUBBLICAZIONE DEI DATI 46](#__RefHeading___Toc340824390)

[16. BIBLIOGRAFIA 47](#__RefHeading___Toc340824391)

[FLOW CHART E TABELLA DEI TEMPI 48](#__RefHeading___Toc340824392)

[I pazienti verranno trattati secondo il seguente schema: 49](#__RefHeading___Toc340824393)

[ALLEGATO 1 – DICHIARAZIONE DI HELSINKY 50](#__RefHeading___Toc340824394)

[ALLEGATO 2 – RIASSUNTO DELLE CARATTERISTICHE DEL PRODOTTO DI KINERET® 50](#__RefHeading___Toc340824395)

[ALLEGATO 3 - QUESTIONARIO HAQ E I CRITERI PER L’ASSEGNAZIONE DEL PUNTEGGIO 50](#__RefHeading___Toc340824396)

**ABBREVIAZIONI**

| ***ACR*** | ***American College of Rheumatology*** | ***HAQ*** | ***Health Assessment Questionnaire*** |
| --- | --- | --- | --- |
| ***ADO*** | Antidiabetici Orali | ***HbA1c*** | Emoglobina glicata |
| ***ADR*** | Reazione avversa | ***IFCC*** | International Federation of Clinical Chemistry |
| ***AE*** | Adverse Event | ***IL-1*** | Interleuchina-1 |
| ***ALT*** | Transaminasi alanino amino transferasi | ***IL-6*** | Interleuchina-6 |
| ***Anti-CCP*** | Anti-cyclic citrullated peptide | ***IL-Ra*** | Recettore interleuchina |
| ***Anti-dsDNAs*** | Anticorpi anti DNA a doppia elica | ***IR*** | Resistenza insulinica |
| ***AR*** | Artrite reumatoide | ***NSAID*** | [Non-steroidal anti-inflammatory](http://www.google.it/url?sa=t&rct=j&q=nsaid &source=web&cd=1&cad=rja&ved=0CCMQFjAA&url=http%3A%2F%2Fen.wikipedia.org%2Fwiki%2FNon-steroidal_anti-inflammatory_drug&ei=Ks49UJPgJoSn0QX9ioDoDQ&usg=AFQjCNH1Sc8G-hzD-teFXL98TYg19UVEtw) drug |
| ***AST*** | Transaminasi aspartato transferasi | ***PPD*** | protein purified derivative |
| ***BMI*** | Indice di Massa Corporea | ***QFT*** | QuantiFERON TB |
| ***BUN*** | Azotemia | ***RCP*** | Riassunto delle Caratteristiche del Prodotto |
| ***CRF*** | Case report form | ***RNF*** | Rete Nazionale Farmacovigilanza |
| ***CRO*** | Clinical Research Organization | ***RWT*** | Relative Wall Thickness |
| ***CRP*** | Proteina C reattiva | ***SAE*** | Serious Adverse Event |
| ***CVD*** | malattie cardiovascolari | ***SDAI*** | Simplified Disease Activity Index |
| ***DAS*** | Disease Activity Score | ***Sm*** |  |
| ***DM2*** | Diabete di tipo 2 | ***SOP*** | Standard Operation Procedure |
| ***Em*** | Early Diastolic velocity | ***SUSAR*** | Suspected Unexpected Serious Adverse Reaction |
| ***ESR*** | Velocità di eritrosedimentazione | ***TDI*** | Tissue Doppler Imaging |
| ***EULAR*** | European League Against Rheumatism |  |  |
| ***GCP*** | Good Clinical Practice | ***TNF-α*** | Fattore di necrosi tumorale-α |
| ***GH*** | Stato di salute complessivo | ***VAS*** | scala visuo-analogica |
| ***NGSP*** | National Glycohemoglobin Standardization Program |  |  |

# INTRODUZIONE

## Informazioni di base e razionale

L’artrite reumatoide (AR) è la forma più comune di artrite infiammatoria e colpisce circa lo 0.5% della popolazione adulta. È caratterizzata da una progressiva infiammazione della membrana sinoviale che determina tumefazione articolare e dolore alla pressione. Nel corso del tempo si verifica un danno strutturale nell’articolazione, evidenziabile radiologicamente, che diminuisce la funzionalità articolare.

I target più promettenti per l’intervento terapeutico sono due citochine infiammatorie, il fattore di necrosi tumorale–α (TNF-α) e l’interleuchina-1 (IL-1); esse rappresentano i fattori critici per l’inizio e la progressione della infiammazione della membrana sinoviale, e per la degradazione della matrice articolare (1).

Attualmente, la ricerca si sta concentrando sui meccanismi di regolazione delle citochine che agiscono per mantenerne o ristabilirne l’omeostasi, sia in condizioni fisiologiche, che patologiche. Questi “inibitori naturali”, resi disponibili dalla clonazione e dall’espressione genica in batteri o in cellule eucariote, possono essere utilizzati per sostenere o amplificare gli effetti dell’attività degli inibitori endogeni delle citochine, presentando in tal modo la possibilità di correggere lo squilibrio nella regolazione di queste molecole in condizioni fisiopatologiche.

Nel corso degli ultimi decenni è diventato sempre più evidente che l’attivazione cronica del sistema immunitario, come nel caso della patogenesi dell’AR, è associata a cambiamenti del metabolismo intermedio, e porta potenzialmente all’aumento del rischio di malattie cardiovascolari (CVD) (2). Diversi report hanno discusso l’associazione tra lo stato di infiammazione cronica e i disordini metabolici, in particolare la resistenza periferica all’insulina (IR) (3). In pazienti affetti da AR, la riduzione del tono muscolare e uno stile di vita sedentario, correlato alla malattia, probabilmente possono contribuire ulteriormente ad indurre lo sviluppo di IR. Nonostante potremmo aspettarci che la prevalenza di diabete di tipo 2 (DM2) aumenti nei pazienti con AR, numerosi studi trasversali non hanno stabilito una definitiva associazione tra queste due condizioni. Oltre a ciò, numerosi studi indipendenti hanno indicato che AR, IR e DM2 si associano alla presenza di molecole in grado di agire su diverse vie della immunoregolazione, compreso il TNF-α, l’interleuchina-6 (IL-6) e IL-1 (4-5).

Studi sperimentali sull’uomo ed sugli animali suggeriscono che il DM2 è associato ad uno squilibrio tra IL-1 e i fattori che ne antagonizzano l’attività; in particolare, si verifica una produzione anomala di IL-1 da parte delle cellule beta, dovuta all’attivazione glucosio-indotta dell’inflammosoma caspasi-1 e alla diminuzione della sintesi di antagonista del recettore dell’ IL-1 (IL-1Ra). L’idea che nel DM2 una carenza relativa dell’antagonista dell’IL-1 possa portare al malfunzionamento glucosio-indotto delle cellule beta è confermata da studi clinici che dimostrano che, nei pazienti con DM2, migliorando l’antagonismo all’IL-1 migliorano la glicemia e la funzione delle cellule beta. In effetti, in corso di DM2, IL-1Ra induce la riduzione sia del HbA_1c_ che della glicemia e della funzione delle cellule beta, ma non influenza il peso o l’indice di massa corporea e la sensibilità all’insulina.

Anakinra (Kineret®) è una molecola ricombinante costituita dalla forma sintetica, non glicosilata, dell’antagonista del IL-1Ra, prodotta in cellule di E.Coli. Esso imita il meccanismo endogeno alla base della regolazione dell’IL-1. Ha un peso molecolare di 17.3 kilodaltons ed è composto da 153 aminoacidi. La principale differenza tra Kineret® e IL-1Ra endogena umana è l’inclusione di un singolo residuo di metionina nell’aminoacido terminale di Kineret®. Kineret® è stato progettato per ridurre lo squilibrio nel metabolismo dell’IL-1; agisce inibendo competitivamente il legame dell’IL-1 con il suo recettore di tipo 1 (IL-1R1), bloccandone così l’attività biologica. Questo processo mima l’attività dell’IL-1Ra endogena.

Recentemente Larsen et al (*Larsen, 2007*) hanno confermato i dati sopra riportati relativi alla capacità di Kineret® di migliorare il controllo glicemico nei pazienti con DM2; molto probabilmente ciò avviene grazie ad un miglioramento della funzione secretoria delle cellule beta. In effetti, si può riscontrare che, nei pazienti che hanno ricevuto Kineret®, il miglioramento della glicemia si correla con l’aumento della capacità secretoria delle cellule beta; negli stessi pazienti tuttavia non si registrano modificazioni della sensibilità insulinica valutate mediante clamp euglicemico iperinsulinemico, indici di sensibilità all'insulina basati sul test di tolleranza al glucosio, espressione genica insulino-regolata nel muscolo scheletrico o mediante i livelli sierici di adipochine. Infine i livelli di BMI dei pazienti rimangono stabili, escludendo così un effetto anoressigenico del farmaco. Comunque, gli Autori non escludono che dosi più elevate di anakinra possano migliorare la sensibilità all’insulina.

## Descrizione del farmaco

Vedi Par. 1.1 Informazione di base e razionale

## Valutazione Rischio/Beneficio

Il Comitato per i medicinali per uso umano (*CHMP*) ha stabilito che i benefici di Kineret® sono superiori ai suoi rischi, nel trattamento dei segni e sintomi dell'artrite reumatoide, in associazione con metotrexato, nei pazienti con risposta inadeguata al solo metotrexato.

Il comitato ha raccomandato il rilascio dell'autorizzazione all'immissione in commercio per Kineret® (*EPAR - EMEA/H/C/363*).

# OBIETTIVI DELLO STUDIO

Lo scopo di questo studio no-profit clinico è di verificare se la somministrazione di anakinra in pazienti affetti da AR, e DM2 come comorbidità, è in grado di determinare un miglioramento del controllo glicemico, oltre che dei segni e sintomi dell’AR, a tal fine determinando un miglioramento della pratica clinica dell’utilizzo di tale farmaco che potrebbe controllare entrambe le morbidità di tali pazienti.

Nel corso delle studio, anakinra sarà utilizzato nell’indicazione e nel dosaggio autorizzati, secondo quanto prescritto dal Riassunto delle Caratteristiche del Prodotto (RCP), con l’obiettivo di migliorare la normale pratica clinica.

# DISEGNO SPERIMENTALE

## Endpoint dello Studio

Endpoint Primario

Variazione del livello di emoglobina glicata, rispetto alle condizioni basali, dopo 3 mesi, 6 mesi, 12 mesi e 24 mesi di trattamento.

Endpoint Secondari

- ***Efficacia***

Valutazione dei segni e sintomi di AR dopo 3 mesi, 6 mesi, 12 mesi e 24 mesi di trattamento:

- Percentuale dei pazienti in remissione (criteri E[uropean League Against Rheumatism](http://www.google.it/url?sa=t&rct=j&q=&esrc=s&source=web&cd=1&ved=0CFcQFjAA&url=http%3A%2F%2Fwww.eular.org%2F&ei=6jj1T7vwLcOE4gSI5O2ABw&usg=AFQjCNGmPXvJMz-hWUB05QV0iKnzlX5b3g&sig2=EcvaoARgAK5nz6BVdL8hQQ) - EULAR) in base alla valutazione del Disability Activity Score (DAS28) e del Simplified Disease Activity Index (SDAI);
- Percentuale dei pazienti migliorati (criteri EULAR) in base alla valutazione del DAS28 e dello SDAI;
- Conta delle articolazioni dolenti (66 articolazioni);
- Conta delle articolazioni tumefatte (68 articolazioni);
- Somministrazione dell’Health Assessment Questionnaire (HAQ).

Parametri di laboratorio:

- Variazione, rispetto alle condizioni basali, dopo 3 mesi, 6 mesi, 12 mesi e 24 mesi di trattamento dei seguenti parametri di laboratorio:
- Proteina C reattiva (CRP) e velocità di eritrosedimentazione (ESR);
- Livelli plasmatici di glucosio
- Livelli plasmatici di trigliceridi, colesterolo totale, colesterolo HDL, colesterolo LDL;
- Microalbuminuria.
- Variazione, rispetto alle condizioni basali, dopo 6 mesi, 12 mesi di trattamento dei seguenti parametri di laboratorio:
- anticorpi anti-DNA a doppia elica (anti-dsDNAs) e anticorpi ANAs.
- Variazione, rispetto alle condizioni basali e dopo 12 mesi di trattamento dei seguenti parametri di laboratorio:

Anticorpi anti-citrullina (anti-CCP) e fattore reumatoide.

Parametri strumentali - Variazione dei parametri ecocardiografici:

- Valutazione, rispetto al basale, dopo 6 mesi, 12 mesi e 24 mesi di trattamento dei seguenti parametri:
- Variazioni funzionali del ventricolo sinistro;
- Variazioni del flusso coronarico;
- Cambiamenti morfologici del ventricolo sinistro.

Altri parametri clinici:

- Valutazione rispetto al basale, dopo 3 mesi, 6 mesi, 12 mesi e 24 mesi di trattamento dell’ indice di massa corporea (BMI);
- Valutazione rispetto al basale, a 12 mesi e a 24 mesi di trattamento della retinopatia diabetica.

***Sicurezza***

La valutazione dei parametri di sicurezza sarà effettuata a 3, 6, 12, 24 mesi di trattamento e saranno osservati i seguenti parametri:

- Valutazione della frequenza e dell’intensità degli eventi avversi (AE) secondo le procedure definite dal protocollo di studio;
- Valutazione dei parametri di laboratorio e strumentali;
- Valutazione dei segni vitali e dell’esame obiettivo;
- Valutazione degli episodi di ipoglicemia.

## Disegno dello studio

Studio clinico ”no-profit”per il miglioramento della pratica clinica, randomizzato, controllato, per gruppi paralleli, in aperto, multicentrico progettato per verificare la possibilità che la somministrazione di anakinra (Kineret®) determini un miglioramento del controllo glicemico, oltre che dei segni e sintomi dell’AR in pazienti affetti da AR e DM2 come comorbidità.

Un totale di 200 pazienti con diagnosi di AR, e DM2 come comorbidità, saranno arruolati in circa 20 centri in Italia.

I pazienti saranno randomizzati in due bracci di trattamento. In entrambi i bracci i farmaci saranno utilizzati nell’indicazione e nei dosaggi autorizzati, secondo la normale pratica clinica. Al braccio in trattamento col farmaco in studio sarà somministrato anakinra, mentre al gruppo di controllo sarà somministrato un altro farmaco utilizzato per il trattamento dell’AR facente parte della classe degli inibitori del TNF-α.

Lo studio prevede una fase iniziale di screening e un periodo di trattamento della durata di 24 mesi.

## Randomizzazione

L’allocazione a uno dei due gruppi di trattamento avverrà in base ad una lista di randomizzazione a blocchi permutati.

# POPOLAZIONE IN STUDIO

## Dimensione del campione

Un totale di 200 pazienti con diagnosi di AR e DM2 saranno arruolati in circa 25 centri in Italia, per ottenere almeno 168 pazienti valutabili (84 per gruppo di trattamento).

## Selezione dei pazienti

### Criteri di inclusione

- Pazienti maschi e femmine di età ≥ 18 anni;
- Precedente diagnosi AR che soddisfi i criteri dell’American College or Rheumatology (ACR)/ EULAR;
- DM2 diagnosticato da almeno 6 mesi secondo i criteri dell’American Diabetes Association (ADA);
- Pazienti con risposta inadeguata ad un precedente trattamento con metotrexato (determinata sulla base della valutazione del medico);
- BMI <35;
- Emoglobina glicata > 7% < 10%;
- Punteggio minimo DAS 28 > 3.2
- Per i pazienti trattati precedentemente con un farmaco biologico, sospensione del trattamento per un periodo di almeno un 1 mese prima dell’arruolamento;
- Per i pazienti in trattamento farmacologico con farmaci ipoglicemizzanti al momento dell’arruolamento, nessuna variazione in termini di farmaco somministrato e di posologia, per un periodo di almeno 3 mesi prima dell’arruolamento;
- Per i pazienti in trattamento farmacologico con altri farmaci al momento dell’arruolamento, nessuna variazione in termini di farmaco somministrato e di posologia per un periodo di almeno 1 mese prima dell’arruolamento;
- Assenza di segni e sintomi riferibili a cardiopatia ischemica;
- Firma del Consenso informato.

### Criteri di esclusione

Un paziente sarà escluso dallo studio se soddisfa uno dei seguenti criteri:

- DM2 diagnosticato da più di 10 anni;
- Infezione acuta in atto o infezione cronica grave in fase di riacutizzazione, che presenti uno o più dei seguenti elementi:

- livelli di CRP > 30 mg/L

- febbre

- trattamento in corso con antibiotici;

- Infezioni granulomatose croniche (ad esempio presenza di tubercolosi diagnosticata su base radiografica o mediante specifici esami di laboratorio);
- Storia clinica di infezioni ricorrenti o presenza di patologie che predispongano a contrarre infezioni;
- Valori di peptide C < 0.5 ng/mL (0.1665 ng/mL);
- Presenza di neutropenia (leucociti < 2000/mm^3^ ) o anemia (emoglobina < 11g/dl per l’uomo e 10g/dl per la donna);
- Presenza di una o più delle controindicazioni previste dal RCP del farmaco in studio (Kineret®):
- Presenza di una o più delle Controindicazioni previste dal RCP del farmaco di controllo (inibitori del TNF-alfa; ATC L04AB);
- Presenza di una o più controindicazioni al trattamento con metotrexato;
- Precedente attacco ischemico o infarto del miocardio;
- Insufficienza cardiaca di classe NYHA III o IV;
- Insufficienza epatica o malattia epatica in atto (valori di ALAT/ASAT aumentati di almeno due volte rispetto ai limiti di normalità);
- Gravidanza in atto o donna in età fertile con non utilizza misure contraccettive;
- Allattamento;
- Partecipazione ad un altro studio clinico 6 mesi prima la randomizzazione;
- Sindrome depressiva grave o altra malattia psichiatrica che, a giudizio del medico, possa precludere la partecipazione del paziente allo studio;
- Presenza di neoplasie maligne note;
- Storia clinicamente significativa di abuso di alcolici o di tossicodipendenza che, a giudizio del medico, possa precludere la partecipazione del soggetto allo studio;
- Qualsiasi condizione che, a giudizio del medico, precluda la possibilità di utilizzare il farmaco in studio o il farmaco di controllo (inibitori del TNF-alfa; ATC L04AB) secondo le condizioni previste dal RCP;
- Qualsiasi altra condizione o parametro di laboratorio che, a giudizio del medico, precluda la partecipazione del soggetto allo studio.

## Criteri per il ritiro dei soggetti dalla sperimentazione

I pazienti devono essere informati della possibilità di interrompere lo studio in qualsiasi momento lo ritengano opportuno. Ogni interruzione deve essere documentata in modo completo nella CRF dallo Sperimentatore.

Lo Sperimentatore dovrà compiere ogni ragionevole sforzo per mantenere il paziente nel protocollo di studio. Se tuttavia sarà necessario escludere il paziente, dovrà essere effettuata una valutazione conclusiva completa. Tutti i risultati di questa valutazione e una descrizione delle ragioni che hanno portato all’esclusione del paziente devono essere registrate nella CRF.

I pazienti potranno interrompere prematuramente lo studio qualora si verifichi una delle seguenti condizioni:

- Richiesta del paziente, anche non motivata;
- Ritiro del consenso da parte del paziente;
- Comparsa di reazioni avverse serie che, a giudizio dello sperimentatore, rendano impossibile la continuazione dello studio;
- Violazione del protocollo di studio che, a giudizio dello sperimentatore, pregiudichino la partecipazione del soggetto allo studio;
- Ogni situazione nella quale lo sperimentatore ritenga che l’esclusione dallo studio sia nell’interesse del paziente;
- Comparsa di criteri di esclusione.

Per quanto possibile, il ritiro dallo studio dovrebbe avvenire dopo una reciproca consultazione. Nel caso di chiusura anticipata tutta la documentazione deve essere ritornata alla CRO Pharmaceutical Development and Services srl.

Sarà cura dello sperimentatore seguire i pazienti per un periodo di tempo appropriato, in relazione all’evento che ha determinato il loro ritiro dallo studio, in maniera da verificare le condizioni cliniche, eventuali controlli di laboratorio, e/o la comparsa di eventi avversi anche a distanza di tempo dalla cessazione della terapia. Le autorità coinvolte devono essere informate.

I dati dei pazienti che hanno interrotto lo studio saranno inclusi nella valutazione complessiva sull’efficacia del farmaco e sulla sua tollerabilità (vedi par. 8.5 gestione dati mancanti).

# TRATTAMENTO DEI SOGGETTI

Sia il farmaco in studio che il farmaco di controllo saranno utilizzati nell’ambito della normale pratica clinica, in conformità all’indicazione e secondo i dosaggi previsti dal rispettivo RCP.

Nel presente studio viene valutato l’effetto di anakinra (Kineret®) sulla riduzione dell’emoglobina glicata, mentre non esistono evidenze scientifiche relative ad un effetto dei farmaci di controllo (inibitori TNF- α) su questo parametro. Gli inibitori TNF- α rappresentano invece un trattamento “usual care” per i pazienti affetti da artrite reumatoide. Di conseguenza, solo anakinra (Kineret®) viene considerato il “prodotto in sperimentazione”, in linea con quanto previsto dalle linee guida per la Buona Pratica Clinica ([*Decreto Ministeriale del 15 luglio 1997*](http://www.agenziafarmaco.gov.it/sites/default/files/Decreto_Ministeriale_del_15_luglio_1997.pdf)*, Allegato 1 Glossario Paragrafo 1.33).*

## Trattamento

### Dosaggio, Posologia e via di somministrazione

I pazienti saranno randomizzati in due gruppi di trattamento.

Gruppo in trattamento col farmaco sperimentale:

I soggetti verranno trattati con anakinra (Kineret®) 100 mg/UID per via sottocutanea; in conformità a quanto previsto dal RCP. Il farmaco sarà somministrato con auto iniezione per via sottocutanea nell’addome o nella coscia.

Il trattamento avrà la durata di 24 mesi.

Tutti i pazienti saranno istruiti dal personale dello studio sulla procedura per la corretta somministrazione del farmaco con iniezione sterile sottocutanea. La somministrazione della prima dose avverrà nel centro sperimentale sotto controllo diretto del personale del centro. Questa supervisione del personale permetterà di verificare la sicurezza del paziente e l’appropriata tecnica di iniezione sottocutanea in modo che tutte le dosi successive possano essere somministrate fuori dal centro.

Gruppo trattato con il farmaco di controllo:

I soggetti verranno trattati con un farmaco appartenente alla classe degli inibitorie del TNF-alfa (ATC L04AB); tali farmaci rappresentano un trattamento “usual care” per i pazienti affetti da artrite reumatoide; la scelta dello specifico prodotto medicinale è lasciata al giudizio del medico. La posologia e le modalità di somministrazione dovranno essere conformi a quanto previsto dal RCP del prodotto medicinale somministrato.

Il trattamento avrà la durata di 24 mesi.

### Trattamenti concomitanti

- *Trattamento con metotrexato*

A tutti i pazienti verrà anche somministrato metotrexato, in conformità a quanto previsto dal RCP ad un dosaggio compreso tra 15 e 20 mg alla settimana (a giudizio del medico).

- *Trattamento ipoglicemizzante*

I soggetti saranno invitati a mantenere le abitudini alimentari e lo stile di vita in corso al momento della randomizzazione.

Al momento dell’arruolamento, i soggetti manterranno il trattamento farmacologico ipoglicemizzante in corso prima dello studio. Nel corso dello studio, il trattamento ipoglicemizzante potrà essere ridotto a giudizio del medico secondo linee guida. Nessun incremento del trattamento ipoglicemizzante sarà consentito.

I pazienti verranno stratificati sulla base dei loro trattamenti antidiabetici, quali:

- sola dieta;
- trattamento con farmaci ipoglicemizzanti orali (ADO);
- trattamento con insulina;
- trattamento con ADO e insulina.
- *Trattamento con farmaci antiinfiammatori NSAIDs e Cortisone*

Al momento dell’arruolamento, i soggetti manterranno il trattamento con farmaci antiinfiammatori steroidei e non-steroidei (NSAID) in corso prima dello studio. Nel corso dello studio, il trattamento con NSAID potrà essere modificato a giudizio del medico; è prevista l’interruzione dello studio in caso di aumento del dosaggio dei farmaci antiinfiammatori steroidei. Il cortisone sarà consentito al dosaggio efficace più basso e, inoltre, non più di 7.5 mg/die di prednisone equivalente.

### Altri trattamenti

Per tutta la durata dello studio tutti i soggetti riceveranno 5 mg di acido folico ogni settimana.

Ogni trattamento che il soggetto ha intrapreso nel mese precedente l’inclusione nello studio, al momento dell’arruolamento o che riceve durante lo studio stesso deve essere registrato nella cartella clinica e riportato nella CRF. Ogni variazione dei trattamenti concomitanti durante il periodo di studio deve essere ugualmente registrata. Per qualsiasi trattamento concomitante dovrà essere indicato: il nome del principio attivo, la dose, la data di inizio e di fine trattamento.

### Speciali avvertenze e precauzioni d’uso

Vedi RCP di Kineret® in allegato 2.

### Confezionamento, etichettatura del farmaco

Lo studio è in aperto; verrà somministrato il prodotto medicinale Kineret®. Le confezioni utilizzate nel corso dello studio sono quelle destinate all’uso commerciale (vedi RCP allegato di Kineret).

Anakinra sarà fornito in siringhe pre-riempite (100 mg Anakinra in 0,67 ml) per auto iniezione. Il farmaco sarà somministrato per via sottocutanea nell’addome o nella coscia.

Il medicinale non utilizzato ed i rifiuti derivati da tale medicinale devono essere smaltiti in conformità alla normativa locale vigente.

### Conservazione del farmaco

Conservare in frigorifero (2 °C - 8 °C). Non congelare.

Conservare nella confezione originale per proteggere il medicinale dalla luce (vedi RCP Kineret® in allegato).

### Distribuzione del farmaco ai pazienti

Trattandosi di uno studio per il miglioramento della normale pratica clinica, in conformità a quanto previsto dall’art. 2, comma 1 del Decreto Ministero della Salute 17 dicembre 2004 (“decreto no-profit”) il farmaco resta a carico del Sistema Sanitario Nazionale. Il farmaco verrà fornito al paziente secondo le modalità previste dai vari centri ospedalieri che partecipano allo studio. Lo Sperimentatore ha l’obbligo di tenere la contabilità di tutto il farmaco somministrato ai pazienti per tutta la durata dello studio, utilizzando la modulistica predisposta.

### Drug Accountability e restituzione dei materiali

Lo sperimentatore inviterà i soggetti partecipanti allo studio a compilare un apposito diario in cui verrà registrata l’assunzione del farmaco. Il diario verrà utilizzato per la drug accountability.

Non è prevista la restituzione, da parte dei pazienti, delle confezioni vuote e non utilizzate dei medicinali.

## Compliance

Lo sperimentatore dovrà istruire i pazienti a riportare a ciascuna visita di controllo il diario attestante l’assunzione del farmaco in studio.

La valutazione della compliance del paziente sarà effettuata mediante la valutazione dei dati riportati nel diario. Tale conteggio dovrà essere riportato nella apposita sezione della CRF.

La percentuale di compliance verrà calcolata mediante la formula:

% Compliance = Numero di unità galeniche assunte X 100

Numero di unità galeniche che avrebbero dovuto essere assunte

Il paziente sarà considerato aderente al protocollo terapeutico se avrà assunto almeno l’80% del farmaco previsto.

## Durata del trattamento

Il protocollo di studio prevede un periodo di trattamento di 24 mesi.

## Sospensione/Interruzione del trattamento

Il paziente deve interrompere il trattamento, e seguire le procedure standard previste dal par. 4.3 del protocollo, se dopo 6 mesi di trattamento si verifica i seguenti casi:

- I pazienti non raggiungono la remissione clinica della AR o almeno un moderato miglioramento clinico (secondo la valutazione dello sperimentatore), che consenta un soddisfacente equilibrio nella qualità della vita;
- I pazienti presentano un peggioramento del compenso glicemico (secondo la valutazione dello sperimentatore).

È prevista l’interruzione dello studio in caso di variazione del dosaggio dei farmaci antiinfiammatori steroidei.

# PROCEDURE DELLO STUDIO

## Valutazione endpoint primario

L’endpoint primario dello studio è rappresentato dalla variazione del livello di emoglobina glicata, rispetto alle condizioni basali, dopo 3 mesi, 6 mesi, 12 mesi e 24 mesi.

I risultati dell’HbA_1c_ dovranno essere riportati utilizzando le unità di misura IFCC (mmol/mol) e le unità derivate NGSP (%), utilizzando l’equazione generale IFCC-NGSP per effettuare la conversione. La relazione tra i due modi di espressione del risultato è la seguente *(Mosca, 2009):*

*HbA1c “allineata DCCT” (%) = 0,0915 × HbA1c “allineata IFCC” (mmol/mol) + 2,15*

## Valutazione degli endpoint secondari

### DAS 28

Valutazione della percentuale dei pazienti in remissione e dei pazienti migliorati utilizzando la scala di valutazione DAS28 *(criteri EULAR;Felson Ann Rheum Dis 2011;70:404-413).*

Il DAS28 è una indice di valutazione dell’artrite reumatoide. È calcolato usando la formula che include il numero di articolazioni tumefatte e il numero di articolazioni dolenti (28 articolazioni):

*DAS28 = 0.56 • √ (t28) + 0.28 • √ (sw28) + 0.70•Ln(ESR) + 0.014•GH*

dove:

t28 = numero di articolazioni dolente su 28

sw28 = numero di articolazioni tumefatte su 28

Ln (ESR) = Logaritmo naturale della VES (mm/ora)

GH = Stato di salute complessivo (scala visuo-analogica)

Il DAS è applicabile alla valutazione della attività di malattia in un determinato momento e alla valutazione della modifica nel tempo della attività della malattia.

I criteri di valutazione DAS28 sono i seguenti:

- paziente migliorato: riduzione del punteggio di attività di malattia da > 5,1 (elevata attività di malattia) a < 3,2 (bassa attività di malattia) oppure da < 3,2 (bassa attività di malattia) a < 2,6 (remissione).
- paziente in remissione: punteggio < 2,6

### SDAI

Valutazione della percentuale dei pazienti in remissione e dei pazienti migliorati utilizzando la scala di valutazione SDAI (criteri EULAR. *criteri EULAR;Felson Ann Rheum Dis 2011;70:404-413*).

Lo SDAI è una misura semplificata di calcolo dell’attività della malattia. Si ottiene dalla somma aritmetica di cinque parametri: numero di articolazioni dolenti (0-28), numero di articolazioni tumefatte (0-28), valutazione globale della attività della malattia da parte del paziente mediante VAS (0-10 cm), valutazione globale della attività della malattia da parte del medico mediante VAS (0-10 cm), concentrazione sierica della proteina C reattiva.

I criteri di valutazione SDAI sono i seguenti:

Paziente migliorato riduzione del punteggio da > 26 (alta attività di malattia) a </= 11(bassa attività di malattia)

Paziente in remissione < 3,3

### Conta delle Articolazioni tumefatte

Saranno valutate 66 articolazioni; verrà riportato il numero di articolazioni tumefatte che saranno classificate come ”presenti” o “assenti” sul modulo di valutazione. Solo 28 articolazioni specifiche saranno considerate come calcolo per DAS28 e SDAI. Le articolazioni trattate farmacologicamente mediante iniezione intra-articolare non saranno valutate per un periodo di 3 mesi dopo il trattamento.

### Conta delle Articolazioni dolenti

Saranno valutate 68 articolazioni dolenti alla mobilizzazione passiva o alla pressione sulla interlinea articolare esprimendo il dolore alle articolazioni come “presente” o “assente” nel modulo di valutazione. Solo 28 articolazioni specifiche saranno considerate nel calcolo DAS28. Le articolazioni trattate farmacologicamente mediante iniezione intra-articolare non saranno valutate per un periodo di 3 mesi dopo il trattamento.

### VAS paziente

#### Valutazione dell’attività della malattia da parte del paziente con scala analogica visiva del dolore (VAS).

Al paziente verrà consegnato un foglio con una linea orizzontale (non graduata, lunga 10 cm, con scritto all’estremità sinistra “assente” e all’estremità destra “massima attività”) e gli verrà chiesto di segnare su questa linea l’intensità del proprio dolore.

### VAS Medico

#### Valutazione dell’attività della malattia da parte del medico mediante VAS.

Sarà valutato il giudizio del medico sul grado complessivo di attività della malattia espresso mediante scala analogica visiva riferendosi per confronto alla precedente rilevazione clinica.

Al medico sarà consegnato un foglio con una linea orizzontale (non graduata, lunga 10 cm, con scritto all’estremità sinistra “assenza di attività della malattia” e all’estremità destra “massima attività della malattia); il medico dovrà segnare su questa linea il grado complessivo di attività della malattia.

### VAS Dolore

#### Valutazione del dolore con scala analogica visiva del dolore (VAS).

Al paziente verrà consegnato un foglio con una linea orizzontale (non graduata, lunga 10 cm, con scritto all’estremità sinistra “assente” e all’estremità destra “Massimo Dolore”) e gli verrà chiesto di segnare su questa linea l’intensità del proprio dolore

### Questionario per la valutazione della salute (HAQ)

Lo Stanford Health Assessment Questionnaire HAQ; *The italian version of the functional disability index of the health assessment questionnarie. A reliable instrument for multicenter studies on rheumatoid arthritis. Ranza et al Clin exp Rheumatol 1993* è uno dei componenti dei criteri ACR ed assegna un punteggio alla capacità di svolgere le attività quotidiane da 0 (bassa disabilità) a 3 (disabilità molto grave). Il questionario HAQ e i criteri per l’assegnazione del punteggio sono riportati in allegato (Allegato n.3).

### Parametri di laboratorio per la valutazione dell’AR:

I seguenti parametri verranno misurati utilizzando le metodiche normalmente in uso nei laboratori dei centri sperimentali:

- proteina C reattiva (CRP);
- velocità di eritrosedimentazione degli eritrociti (ESR);
- fattore reumatoide;
- anticorpi anti-citrullina (anti-CCP) valutati mediante test di seconda generazione, CCP2/ELISA oppure un test di terza generazione, CCP3/ELISA;
- anticorpi anti-nucleo (ANAs) valutati mediante valutati mediante test ELISA;
- anticorpi anti-DNA a doppia elica (anti-dsDNAs) valutati mediante test radioimmunologico di Farr o l’immunofluorescenza su Crithidia luciliae, oppure ELISA.

### Parametri di laboratorio per la valutazione del DM:

I seguenti parametri verranno misurati utilizzando le metodiche normalmente in uso nei laboratori dei centri sperimentali:

- glicemia a digiuno, trigliceridemia, colesterolo totale, colesterolo HDL e colesterolo LDL.
- microalbuminuria valutata mediante il rapporto albumina/creatinina (ACR) su un campione di urina raccolto al mattino.

### Parametri strumentali ecocardiografici:

- Variazioni funzionali del ventricolo sinistro valutate mediante TDI (Tissue Doppler Imaging):
  - radial peak myocardial systolic velocity Sm: cm/sec;
  - early diastolic velocity Em: cm/sec;
  - strain and strain rate both in radial and longitudinal contractility: Cm/sec
- Variazioni del flusso coronarico valutate mediante misurazione del flusso aortico (Pulse Wave Velocity technique): L/min;
- Cambiamenti morfologici del ventricolo sinistro valutati mediante Relative Wall Thickness (RWT): mm.

### Valutazione della retinopatia diabetica

#### La retinopatia verrà valutata mediante esame del fondo oculare, in accordo con quanto previsto dalle linea guida AMD 2002.

### Indice di massa corporea (BMI)

#### L’indice di massa corporea verrà valutato usando la seguente formula:

BMI = peso (kg)/ altezza^2^ (m^2^)

### Endpoint di Safety

- Valutazione dei segni vitali e dell’esame obiettivo;
- Valutazione dei parametri di laboratorio e strumentali;
- Valutazione del tipo, frequenza e intensità degli eventi avversi (AE) secondo le procedure definite dal protocollo di studio (vedi capitolo 7);
- Valutazione degli episodi di ipoglicemia, classificati come:
- *Grave*: episodio che mette in pericolo la vita del paziente e richiede un intervento medico immediato;
- *Moderato*: livelli plasmatici di glucosio < 56 mg/dl e/o presenza di sintomatologia che non richiede un intervento medico immediato;
- *Lieve*: livelli plasmatici di glucosio > 56 mg/dl o presenza di sintomatologia che non ha richiesto la misurazione della glicemia;
- Andrà inoltre indicato se l’episodio glicemico è intercorso durante la notte tra le 23:00 e le 5:59 incluse.

## Esami di laboratorio generali

Oltre agli esami già elencati nel paragrafo 6.2 saranno effettuati i seguenti esami di laboratorio.

### Ematologia

### Esame emocromocitometrico con formula leucocitaria e conta piastrinica.

### Chimica clinica

Azotemia (BUN), creatininemia, bilirubina, transaminasi (ALAT/ASAT), fosfatasi alcalina, sodio, potassio, calcio, acido urico, proteine totali, albumina.

### Analisi delle urine:

Esame completo delle urine.

Tutti i suddetti esame verranno effettuati utilizzando le metodiche normalmente in uso nei laboratori dei centri sperimentali.

Il prelievo di sangue dove essere eseguito dopo la valutazione del dolore, la somministrazione dei questionari e la valutazione dei segni vitali.

## Calendario delle visite

Ciascun paziente sarà sottoposto a 6 visite:

Visita 0 (Visita di screening);

Visita 1 – mese 0 (Visita di inizio studio; entro 10 giorni dalla Visita 0);

Visita 2 – mese 3 (dopo 3 mesi dalla Visita 1);

Visita 3 – mese 6 (dopo 6 mesi dalla Visita 1);

Visita 4 – mese 12 (dopo 12 mesi dalla Visita 1);

Visita 5 – mese 24 (Visita finale; dopo 24 mesi dalla Visita 1).

Per ogni paziente la durata dello studio sarà di 24 mesi complessivi.

***Visita 0 (Visita di screening)***

Nel corso della Visita 0, avverrà la selezione dei pazienti. Lo Sperimentatore, dopo aver esaurientemente informato il paziente e dopo avergli lasciato tutto il tempo necessario per i chiarimenti del caso, lo inviterà a firmare il Consenso Informato.

Dopo la firma del Consenso Informato, il paziente sarà sottoposto a visita medica, prelievo ematico e urinario, e test strumentali specifici per verificare la rispondenza ai criteri di inclusione/esclusione.

In particolare, saranno effettuate le seguenti valutazioni:

- Firma del consenso informato;
- Dati demografici (iniziali, data di nascita, sesso, altezza, peso);
- Anamnesi;
- Valutazione dei segni vitali (pressione arteriosa, frequenza cardiaca, temperatura corporea);
- Esame obiettivo;
- BMI;
- Valutazione clinica dell’AR mediante:
  - punteggi diagnostici specifici DAS-28;
  - punteggi diagnostici specifici SDAI;
  - conta delle articolazioni dolenti (66 articolazioni);
  - conta delle articolazioni tumefatte (68 articolazioni);
  - somministrazione dell’HAQ.
- Valutazione biochimica dell’AR mediante: CRP, ESR, anti-CCP, anti-dsDNAs, ANAs, fattore reumatoide;
- Valutazione HbA_1c_;
- Glicemia a digiuno, trigliceridi, colesterolo totale, colesterolo HDL, colesterolo LDL;
- Test specifico per la diagnosi di tubercolosi (PPD e/o QFT);
- Esame radiografico del torace (non necessario se già effettuato nei 3 mesi precedenti la visita);
- Elettrocardiogramma a 12 derivazioni;
- Valutazione di segni e sintomi di cardiopatia ischemica;
- Esame emocromocitometrico con formula leucocitaria e conta piastrinica;
- Esami di chimica clinica;
- Esame completo delle urine e microalbuminuria;
- Test di gravidanza;
- Registrazioni patologie concomitanti;
- Registrazione dei trattamenti farmacologici concomitanti e/o di quelli intrapresi dal paziente nei 3 mesi precedenti la visita di arruolamento.

Alcuni delle suddette valutazioni potranno non essere effettuate se il paziente è già in possesso dei risultati delle stesse. L’utilizzabilità dei risultati già in possesso del paziente ai fini dello studio saranno lasciati alla discrezione dello sperimentatore, ad eccezione dell’esame radiografico del torace (che deve essere stato effettuato nei 3 mesi precedenti la visita).

Al termine delle visita, il paziente sarà invitato a presentarsi per la visita successiva (Visita 1 – Visita di inizio studio), che dovrà essere effettuata dopo che lo Sperimentatore e/o il paziente saranno entrati in possesso di tutti i risultati delle valutazioni effettuate nella Visita 0, e comunque non oltre 10 giorni dalla data della Visita 0.

***Visita 1 (Visita di inizio studio - mese 0)***

Questa visita dovrà essere effettuata entro e non oltre 10 giorni dalla Visita 0.

Nel corso della Visita 1, saranno verificati i risultati degli esami di laboratorio e degli esami strumentali effettuati nel corso della Visita 0. Successivamente lo Sperimentatore, dopo aver verificato che siano rispettati tutti i criteri di inclusione e nessun criterio di esclusione, procederà all’arruolamento dei pazienti giudicati idonei e alla loro randomizzazione in uno dei due gruppi di trattamento.

Dopo la randomizzazione, i pazienti verranno sottoposti ad una serie di procedure e, successivamente, sarà consegnato loro il Diario del Paziente per prendere nota dell’assunzione dei farmaci il paziente sarà istruito dallo sperimentatore sulla compilazione del Diario. Verrà quindi somministrata la prima dose del farmaco in sperimentazione e del farmaco di controllo; il paziente verrà istruito dallo Sperimentatore sulle modalità di auto-somministrazione dei farmaci. La somministrazione della prima dose dei farmaci dovrà essere registrata nella CRF e nel Diario del paziente. Il farmaco in sperimentazione e il farmaco di controllo verranno consegnati al paziente, tramite la farmacia dell’ospedale o secondo le modalità previste dalle procedure in vigore presso i vari centri sperimentali.

Lo sperimentatore inviterà il paziente a ripresentarsi dopo 3 mesi (+ 7 giorni) dalla data della Visita 1 portando con sé il diario del paziente; egli inoltre istruirà il paziente a mettersi tempestivamente in contatto con lui nell’eventualità di insorgenza di disturbi di qualsiasi natura.

In particolare, nel corso della Visita 1 i pazienti saranno sottoposti alle seguenti procedure:

- Valutazione dei criteri di inclusione/esclusione;
- Randomizzazione;
- Valutazione dei parametri ecocardiografici:
- Variazioni funzionali del ventricolo sinistro valutate mediante TDI;
- Variazioni del flusso coronarico valutate mediante Pulse Wave Velocity;
- Cambiamenti morfologici del ventricolo sinistro valutati RWT;
- Valutazione della retinopatia diabetica mediante esame del fondo oculare;
- Somministrazione della prima dose del farmaco sperimentale o di quello di controllo;
- Consegna del Diario del paziente;
- Profilassi anti-tubercolare specifica per pazienti senza malattia in atto, ma risultati positivi all’esame radiografico del torace e/o al test specifico per la diagnosi di tubercolosi (PPD e/o QFT).

***Visita 2 (mese 3)***

La Visita 2 verrà effettuata dopo 3 mesi (+ 7 giorni) dalla data della Visita 1.

Nel corso della Visita 2, dopo la verifica dei criteri di inclusione/esclusione, la rilevazione degli eventi avversi e la valutazione della compliance al trattamento (effettuata mediante l’esame del Diario del paziente) il paziente verrà sottoposto alle valutazioni previste. Al termine della visita, al paziente verrà consegnato il Diario del paziente. Il farmaco in sperimentazione e il farmaco di controllo verranno consegnati al paziente , tramite la farmacia dell’ospedale o secondo le modalità previste dalle procedure in vigore presso i vari centri sperimentali.

Lo sperimentatore inviterà il paziente a ripresentarsi dopo 3 mesi (+ 7 giorni) dalla data della Visita 2 portando con sé il Diario del paziente egli inoltre istruirà il paziente a mettersi tempestivamente in contatto con lui nell’eventualità di insorgenza di disturbi di qualsiasi natura.

In particolare, nel corso della Visita 2 i pazienti saranno effettuate le seguenti procedure:

- Verifica dei criteri di inclusione ed esclusione applicabili;
- Rilevazione degli eventi avversi;
- Verifica della compliance mediante valutazione del Diario del paziente;
- Registrazioni patologie concomitanti;
- Registrazione dei trattamenti concomitanti;
- Valutazione dei segni vitali (pressione arteriosa, frequenza cardiaca, temperatura corporea);
- Esame obiettivo;
- BMI;
- Valutazione clinica dell’AR mediante:
  - utilizzo di punteggi diagnostici specifici DAS-28;
  - utilizzo di punteggi diagnostici specifici SDAI;
  - conta delle articolazioni dolenti (66 articolazioni);
  - conta delle articolazioni tumefatte (68 articolazioni);
  - somministrazione dell’HAQ.
- Valutazione biochimica dell’AR mediante: CRP, ESR;
- Valutazione HbA_1c_;
- Glicemia a digiuno, trigliceridemia, colesterolo totale, colesterolo HDL, colesterolo LDL;
- Esame emocromocitometrico con formula leucocitaria e conta piastrinica;
- Esami di chimica clinica;
- Esame completo delle urine e microalbuminuria;
- Ritiro del Diario del paziente compilato e consegna del Diario successivo.

***Visita 3 (mese 6)***

La Visita 3 verrà effettuata dopo 3 mesi (+ 7 giorni) dalla data della Visita 2.

Nel corso della Visita 3, dopo la verifica dei criteri di inclusione/esclusione, la rilevazione degli eventi avversi e la valutazione della compliance al trattamento, effettuata mediante l’esame del Diario del paziente il paziente verrà sottoposto alle valutazioni previste. Al termine della visita, al paziente verrà consegnato il Diario del paziente. Il farmaco in sperimentazione e il farmaco di controllo verranno consegnati al paziente , tramite la farmacia dell’ospedale o secondo le modalità previste dalle procedure in vigore presso i vari centri sperimentali.

Lo sperimentatore inviterà il paziente a ripresentarsi dopo 6 mesi (+ 7 giorni) dalla data della Visita 3 portando con sé il Diario del paziente; egli inoltre istruirà il paziente a mettersi tempestivamente in contatto con lui nell’eventualità di insorgenza di disturbi di qualsiasi natura.

In particolare, nel corso della Visita 3 i pazienti saranno effettuate le seguenti procedure:

- Verifica dei criteri di inclusione ed esclusione applicabili;
- Rilevazione degli eventi avversi;
- Verifica della compliance mediante valutazione del Diario del paziente;
- Registrazione patologie concomitanti;
- Registrazione dei trattamenti concomitanti;
- Valutazione dei segni vitali (pressione arteriosa, frequenza cardiaca, temperatura corporea);
- Esame obiettivo;
- BMI;
- Valutazione clinica dell’AR mediante:
  - utilizzo di punteggi diagnostici specifici DAS-28;
  - utilizzo di punteggi diagnostici specifici SDAI;
  - conta delle articolazioni dolenti (66 articolazioni);
  - conta delle articolazioni tumefatte (68 articolazioni);
  - somministrazione dell’HAQ;
- Valutazione biochimica dell’AR mediante: CRP, ESR, anti-dsDNAs, ANAs;
- Valutazione HbA_1c_;
- Glicemia a digiuno; trigliceridemia, colesterolo totale, colesterolo HDL, colesterolo LDL;
- Esame emocromocitometrico con formula leucocitaria e conta piastrinica;
- Esami di Chimica clinica;
- Esame completo delle urine e microalbuminuria;
- Valutazione dei parametri ecocardiografici:
- Variazioni funzionali del ventricolo sinistro valutate mediante TDI;
- Variazioni del flusso coronarico valutate mediante Pulse Wave Velocity;
- Cambiamenti morfologici del ventricolo sinistro valutati mediante RWT;
- Valutazione della retinopatia diabetica mediante esame del fondo oculare;
- Ritiro del Diario compilato del paziente compilato e consegna del Diario successivo;
- Esame radiografico del torace per i pazienti che hanno ricevuto la profilassi anti-tubercolare.

***Visita 4 (mese 12)***

La Visita 4 verrà effettuata dopo 6 mesi (+ 7 giorni) dalla data della Visita 3.

Nel corso della Visita 4, dopo la verifica dei criteri di inclusione/esclusione, la rilevazione degli eventi avversi e la valutazione della compliance al trattamento, effettuata mediante l’esame del Diario del paziente, il paziente verrà sottoposto alle valutazioni previste. Al termine della visita, al paziente verrà consegnato il Diario del paziente. Il farmaco in sperimentazione e il farmaco di controllo verranno consegnati al paziente , tramite la farmacia dell’ospedale o secondo le modalità previste dalle procedure in vigore presso i vari centri sperimentali.

Lo sperimentatore inviterà il paziente a ripresentarsi dopo 12 mesi (+ 7 giorni) dalla data della Visita 4 portando con sé il Diario del paziente ; egli inoltre istruirà il paziente a mettersi tempestivamente in contatto con lui nell’eventualità di insorgenza di disturbi di qualsiasi natura.

In particolare, nel corso della Visita 4 i pazienti saranno effettuate le seguenti procedure:

- Verifica dei criteri di inclusione ed esclusione applicabili;
- Rilevazione degli eventi avversi;
- Verifica della compliance mediante valutazione del Diario del paziente e Registrazioni patologie concomitanti;
- Registrazione dei trattamenti concomitanti;
- Valutazione dei segni vitali (pressione arteriosa, frequenza cardiaca, temperatura corporea);
- Esame obiettivo;
- BMI;
- Valutazione clinica dell’AR mediante:
  - utilizzo di punteggi diagnostici specifici DAS28;
  - utilizzo di punteggi diagnostici specifici SDAI;
  - conta delle articolazioni dolenti (66 articolazioni);
  - conta delle articolazioni tumefatte (68 articolazioni);
  - somministrazione dell’ HAQ.
- Valutazione biochimica dell’AR mediante: CRP, ESR, anti-CCP, anti-dsDNAs, ANAs, fattore reumatoide;
- Valutazione HbA_1c_;
- Glicemia a digiuno; trigliceridi, colesterolo totale, colesterolo HDL, colesterolo LDL;
- Esame emocromocitometrico con formula leucocitaria e conta piastrinica;
- Esami di chimica clinica;
- Esame completo delle urine e microalbuminuria;
- Valutazione dei parametri ecocardiografici:
- Variazioni funzionali del ventricolo sinistro valutate mediante TDI;
- Variazioni del flusso coronarico valutate mediante Pulse Wave Velocity;
- Cambiamenti morfologici del ventricolo sinistro valutati mediante RWT;
- Valutazione della retinopatia diabetica mediante esame del fondo oculare;
- Ritiro del Diario del paziente compilato e consegna del Diario successivo.

***Visita 5 (Visita finale - mese 24)***

La Visita 5 verrà effettuata dopo 12 mesi (+ 7 giorni) dalla data della Visita 4.

Nel corso della Visita 5, dopo la verifica dei criteri d’inclusione ed esclusione, la rilevazione degli eventi avversi e la valutazione della compliance al trattamento, effettuata mediante l’esame del Diario del paziente , il paziente verrà sottoposto alle valutazioni previste. Al termine della visita si procederà alle valutazioni finali.

In particolare, nel corso della Visita 5 i pazienti saranno effettuate le seguenti procedure:

- Verifica dei criteri di inclusione ed esclusione applicabili;
- Rilevazione degli eventi avversi;
- Verifica della compliance mediante valutazione del Diario del paziente
- Registrazione patologie concomitanti;
- Registrazione dei trattamenti concomitanti;
- Valutazione dei segni vitali (pressione arteriosa, frequenza cardiaca, temperatura corporea);
- Esame obiettivo;
- BMI;
- Valutazione clinica dell’AR mediante:
  - utilizzo di punteggi diagnostici specifici DAS28;
  - utilizzo di punteggi diagnostici specifici SDAI;
  - conta delle articolazioni dolenti (66 articolazioni);
  - conta delle articolazioni tumefatte (68 articolazioni);
  - somministrazione dell’HAQ;.
- Valutazione biochimica dell’AR mediante: CRP, ESR;
- Valutazione HbA_1c_;,
- Glicemia a digiuno; trigliceridi, colesterolo Totale, colesterolo HDL, colesterolo LDL;
- Esame emocromocitometrico con formula leucocitaria e conta piastrinica;
- Esami di chimica clinica;
- Esame completo delle urine e microalbuminuria;
- Valutazione dei parametri ecocardiografici:
- Variazioni funzionali del ventricolo sinistro valutate mediante TDI
- Variazioni del flusso coronarico valutate mediante Pulse Wave Velocity;
- Cambiamenti morfologici del ventricolo sinistro valutati mediante RWT.
- Valutazione della retinopatia diabetica mediante esame del fondo oculare;
- Ritiro del Diario del paziente compilato;
- Procedure di chiusura dello studio.

# VALUTAZIONE DELLA SICUREZZA E TOLLERABILITA’

Nel corso di tutto lo studio gli sperimentatori monitoreranno sistematicamente ciascun soggetto per la presenza di segni clinici e risultati di laboratorio indicativi di eventi avversi.

Lo sperimentatore valuterà e registrerà gli eventi avversi in modo dettagliato utilizzando l’apposita sezione della CRF, e inserendo la data e l’ora d’insorgenza, la descrizione, la gravità, la durata e l’esito, la relazione con il farmaco in studio, la diagnosi finale, se conosciuta, ed ogni azione intrapresa. Sulla CRF verranno registrati sia gli eventi avversi dovuti ad una specifica segnalazione riportata dal personale sanitario, sia quelli riferiti spontaneamente dal soggetto. Tutti gli eventi avversi saranno monitorati fino ad una loro adeguata risoluzione.

## Definizioni

### Evento avverso (AE)

Un evento avverso è definito come un evento medico sfavorevole in un paziente, o in un soggetto sottoposto a sperimentazione clinica, al quale è stato somministrato un prodotto farmaceutico; tale evento non presenta necessariamente una relazione causale con il trattamento somministrato. Un evento avverso può quindi essere qualsiasi segno negativo e non voluto (inclusi valori di laboratorio anormali), sintomo o malattia che presenti una associazione temporale con la somministrazione di un farmaco in sperimentazione, sia che venga considerato correlato o meno con il trattamento stesso.

Tale evento può derivare sia dall’uso del farmaco come previsto dal protocollo o dagli stampati, che da una overdose, accidentale o intenzionale, che dal suo abuso o sospensione. Ogni peggioramento di una condizione o malattia pre-esistente è considerato un evento avverso. Le anomalie di laboratorio o i cambiamenti nei segni vitali sono considerati eventi avversi solo se comportato l'interruzione dello studio, necessitano di intervento medico e/o se il ricercatore li considera eventi avversi.

Un evento avverso “trattamento-emergente” è definito come un qualsiasi evento avverso con insorgenza o peggioramento segnalato dal soggetto dal momento in cui viene somministrata la prima dose di farmaco in studio fino a che siano trascorse cinque emivite dopo l’interruzione della somministrazione.

Uno specifico intervento chirurgico/procedura medica programmata che si svolge nel corso dello studio non sarà considerato un evento avverso se effettuato per una condizione preesistente e se programmato prima dell’ingresso nello studio (prima della firma del consenso informato). Tuttavia, se la condizione preesistente peggiora inaspettatamente durante lo studio (ad esempio se l’intervento viene eseguito prima del previsto), in questo caso il peggioramento della condizione clinica che ha determinato l'intervento chirurgico/procedura sarà considerato un evento avverso.

### Evento avverso serio (SAE)

Gli eventi avversi seri (SAE) sono definiti come segue:

1. Morte del paziente: un evento che provoca la morte del paziente.
2. Pericolo di vita: un evento che, a giudizio dello sperimentatore, sarebbe risultato immediatamente fatale se non fosse stato effettuato un intervento medico. Questa definizione non comprende un evento che sarebbe stato fatale se fosse avvenuto in una forma più grave.
3. Ricovero in ospedale: un evento che determina un ricovero in ospedale per un periodo di tempo di qualsiasi durata. Questa definizione non comprende una visita in pronto soccorso o presso una struttura ambulatoriale.
4. Prolungamento dell’ ospedalizzazione: un evento che si verifica mentre il soggetto in studio è ricoverato in ospedale e che prolunga la durata del ricovero.
5. Anomalia congenita: una anomalia rilevata in corrispondenza o dopo la nascita, o qualsiasi anomalia che si traduce in perdita del feto.
6. Persistente o significativa invalidità/incapacità: un evento che si traduce in una condizione che interferisce in modo sostanziale con le attività della vita quotidiana di un soggetto partecipante ad una sperimentazione clinica. La definizione di invalidità non è destinata ad includere gli eventi medici minori, come, ad esempio, mal di testa, nausea, vomito, diarrea, influenza e trauma accidentale (ad esempio, una distorsione alla caviglia).
7. Evento medico importante che richiede l’intervento medico o chirurgico per prevenire esiti gravi: un evento medico importante che possa non mettere in immediato pericolo di vita o provocare la morte o l’ospedalizzazione, ma che, secondo il giudizio del medico, possa compromettere l’integrità del soggetto o possa richiedere l’intervento medico o chirurgico per prevenire uno degli esiti di cui sopra (cioè, morte del soggetto, pericolo di vita, ospedalizzazione, prolungamento dell'ospedalizzazione, anomalia congenita, persistente o significativa invalidità/incapacità). Esempi di tali eventi sono: broncospasmo allergico che richiede un trattamento intensivo in pronto soccorso o a casa; discrasia ematica; convulsioni che non comportano il ricovero del paziente; sviluppo di dipendenza dal farmaco o abuso dello stesso.
8. Trasmissione di agenti infettivi da parte del farmaco in studio.
9. Aborto spontaneo: aborto spontaneo da parte del soggetto che partecipa allo studio.
10. Aborto elettivo: aborto elettivo al quale viene sottoposto il soggetto che partecipa allo studio.

Se un evento avverso soddisfa uno dei suddetti criteri, esso deve essere segnalato al responsabile per la sicurezza dell’unità di sicurezza dell'organizzazione di ricerca clinica (CRO) come SAE secondo le modalità di cui al paragrafo 7.5 (entro 24 ore dal verificarsi dell’evento o dalla notifica da parte del centro sperimentale).

### Reazione avversa da farmaco (ADR)

Per quanto riguarda i prodotti medicinali già in commercio, come quelli utilizzati nel presente studio, per reazione avversa da farmaco (ADR) si intende una risposta al farmaco che sia nociva e non voluta.

### Reazione avversa da farmaco inattesa (SUSAR)

Per reazione avversa da farmaci inattesa (Suspected Unexpected Serious Adverse Reaction; SUSAR) si intende una ADR il cui tipo o intensità non corrisponde alle informazioni esistenti sul prodotto. Nel presente studio i farmaci vengono somministrati secondo l’ indicazione e il dosaggio previsto dal Riassunto delle Caratteristiche del Prodotto; pertanto il documento di riferimento relativo alle informazioni esistenti sul prodotto è rappresentato dal RCP stesso, che sostituisce l’Investigator’s Brochure.

## Intensità degli eventi avversi

Lo sperimentatore utilizzerà le seguenti definizioni per determinare il grado di severità degli eventi avversi:

*Lieve:* l’evento avverso è transitorio e ben tollerato dal soggetto e non influenza con le normali attività quotidiane del soggetto.

*Moderato:* l’evento avverso causa malessere al soggetto e determina l’interruzione delle normali attività

*Severo:* l’evento avverso causa una considerevole interferenza con la normale attività del soggetto e può determinarne l’incapacità a svolgere le normali attività o metterne in pericolo la vita.

## Relazione con il farmaco in studio

Lo sperimentatore userà le categorie di causalità definite dalla WHO per valutare la relazione tra evento avverso e somministrazione del farmaco in studio; le categorie sono le seguenti:

| **Termine di causalità** | **Criteri di valutazione** |
| --- | --- |
| **Certo** | - Evento o alterazione di un parametro di laboratorio con una plausibile relazione temporale con l’assunzione del farmaco. - Non può essere spiegato dalla malattia per il quale il farmaco è impiegato o dall’assunzione di farmaci concomitanti. - Risposta plausibile (farmacologicamente o clinicamente) alla sospensione del farmaco. (“de-challenge”) - Evento farmacologicamente o clinicamente ben definito (ad es. un patologia oggettiva e specifica, o un fenomeno farmacologicamente riconosciuto) - Risposta soddisfacente alla eventuale ri-somministrazione del farmaco (“re challenge”). |
| **Probabile** | - Evento o alterazione di un parametro di laboratorio con una ragionevole relazione temporale con l’assunzione del farmaco. - E’ improbabile che sia attribuibile alla malattia per il quale il farmaco è impiegato o all’assunzione di farmaci concomitanti. - Risposta clinica ragionevole alla sospensione del farmaco. (“dechallenge”) - Non è richiesta la ri-somministrazione del farmaco (“re-challenge”) |
| **Possibile** | - Evento o alterazione di un parametro di laboratorio con una ragionevole relazione temporale con l’assunzione del farmaco. - Potrebbe essere spiegato anche dalla malattia o dall’assunzione di farmaci concomitanti. - Le informazioni relative alla sospensione del farmaco possono essere insufficienti o non chiare. |
| **Improbabile** | - Evento o alterazione di un parametro di laboratorio con una relazione temporale con l’assunzione del farmaco che rende la relazione improbabile (ma non impossibile) - La malattia o i farmaci concomitanti forniscono una spiegazione plausibile |
| **Non Correlata** | - Evento o alterazione di un parametro di laboratorio per il quale la relazione temporale con la somministrazione del farmaco esclude la relazione causale. - L’evento clinico è ragionevolmente determinato da altri farmaci, sostanze chimiche o patologie concomitanti |

## Registrazione degli eventi avversi

I SAE devono essere registrati dal momento in cui il paziente firma il consenso informato fino a quando sono trascorse 5 emivite dopo l’interruzione della somministrazione del farmaco, sia che tali eventi siano stati raccolti durante contatto telefonico e/o visite programmate, sia che essi siano stati riportati spontaneamente dal paziente.

Saranno raccolti tutti gli eventi avversi non seri che si verificano dal momento in cui viene iniziata la somministrazione del farmaco in studio, fino a quando sono trascorse 5 emivite dopo l’interruzione della somministrazione del farmaco, sia che tali eventi siano stati raccolti durante contatto telefonico e/o visite programmate, sia che essi siano stati riportati spontaneamente dal paziente.

Gli episodi di glicemia verranno registrati in uno specifico campo della CRF.

In caso di eventi avversi correlati alla somministrazione del farmaco dovrebbe essere presa in considerazione la riduzione del dosaggio di metrotrexato.

## Segnalazione degli eventi avversi

Le modalità di segnalazione degli eventi avversi sono le seguenti:

### SUSAR con esito letale/pericolo di vita

Lo sperimentatore comunica immediatamente, o comunque entro 24 ore dal momento in cui ne viene a conoscenza, allo sponsor e al responsabile per la sicurezza dell’unità di sicurezza della CRO Pharmaceutical Development and Services srl le SUSAR che abbiano avuto esito letale o che mettano in pericolo di vita dei soggetti; i recapiti da utilizzare per la comunicazione sono i seguenti:

Sponsor – Università degli studi dell’Aquila

Prof. Roberto Giacomelli

[roberto.giacomelli@cc.univaq.it](mailto:roberto.giacomelli@cc.univaq.it)

Tel: 0862433395

PharmaD&S

Dott.ssa Irene Barneschi

[ibarneschi@pharmades.it](mailto:ibarneschi@pharmades.it )

Tel: 055-7224179

Lo sperimentatore provvede alla registrazione dei suddetti eventi nella Rete Nazione di Farmacovigilanza (RNF) secondo le modalità ed i tempi previsti dalla normativa in tema di farmacovigilanza attualmente in vigore.

### Evento Avverso Serio (SAE)

Ad esclusione dei SUSAR di cui al paragrafo precedente, ogni SAE, sia correlato che non correlato al farmaco, dovrà essere comunicato, da parte dello sperimentatore, allo sponsor e al responsabile per la sicurezza dell’unità di sicurezza della CRO Pharmaceutical Development and Services srl entro 3 giorni dal momento in cui il SAE si è verificato o comunque dal momento in cui lo sperimentatore ne è venuto a conoscenza; i recapiti da utilizzare per la comunicazione sono i seguenti:

Sponsor – Università degli studi dell’Aquila

Prof. Roberto Giacomelli MD-PhD

[roberto.giacomelli@cc.univaq.it](mailto:roberto.giacomelli@cc.univaq.it)

Tel: 0862433395

PharmaD&S

Dott.ssa Irene Barneschi

ibarneschi@pharmades.it

Tel: 055-7224179

Lo sperimentatore provvede alla registrazione dei SAE nella RNF, secondo le modalità ed i tempi previsti dalla normativa in tema di farmacovigilanza attualmente in vigore.

**7.6 Gravidanza**

Alle pazienti che rimangono gravide nel corso dello studio sarà interrotta la somministrazione del farmaco.

L’aborto spontaneo o indotto è considerato un SAE e deve essere comunicato entro 24 ore dal momento in cui lo sperimentatore ne è venuto a conoscenza.

# ANALISI STATISTICA

## Calcolo della dimensione del campione

Questo studio clinico è stato progettato per dimostrare la superiorità di anakinra rispetto al trattamento con un farmaco anti TNF-α in termini di efficacia nel migliorare il controllo glicemico oltre che nel ridurre i segni e i sintomi dell’artrite reumatoide in pazienti affetti da artrite reumatoide e diabete di tipo 2 come comorbidità. La dimostrazione della superiorità di anakinra è basata sulla determinazione di una differenza media di 0.25 punti percentuali di emoglobina glicata tra i due gruppi di trattamento. *(Larsen, 2007).*

È stato stimato che assumendo :

- Errore di tipo 1: α = 0.05
- Potenza: 1-β = 0.90
- Differenza al numeratore di: 0.0625 (differenza di emoglobina glicata attesa tra i trattamenti)
- Deviazione standard: 0.5


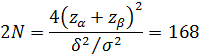


per calcolare la superiorità del farmaco sperimentale rispetto al farmaco controllo sarà valutato un campione di 168 pazienti, 84 per gruppo di trattamento.

Considerando un withdrawal rate del 10%, nonché un fattore di inflazione pari a 1.01 derivante dall’utilizzo delle soglie di arresto di O’Brian-Fleming (vedi 8.6), il campione totale dovrà essere almeno di 200, cioè 100 soggetti per gruppo di trattamento.

## Modello statistico

### Analisi statistica

L’analisi statistica effettuerà un confronto tra gli effetti del trattamento nei due gruppi, aggiustando la covarianza come sesso e età, per mezzo di modelli lineari misti
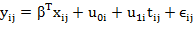
 che affronterà il carattere longitudinale del disegno dello studio.

## Procedure di randomizzazione

I pazienti sono assegnati a ciascun gruppo di trattamento utilizzando il metodo della randomizzazione a blocchi permutati per evitare possibili squilibri.

Quando il paziente entra nello studio viene assegnato ad uno dei due gruppi di trattamento definiti come A e B. All’interno di ogni blocco, l’ordine del trattamento è randomizzato e la dimensione del gruppo rimane sconosciuta al medico, per evitare qualsiasi distorsione nell’assegnazione. Per esempio, assumiamo un blocco di dimensione 4, con due A e due B.

Per ogni gruppo di 4 ci sono
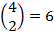
 combinazioni possibili per assegnare il trattamento, i.e:

{(A A B B); (A B A B); (A B B A); (B A A B); (B A B A); (B B A A)}.

Lo schema di randomizzazione sceglie una di queste sei possibilità con uguale probabilità (cioè 1/6) e procede come segue. I primi quattro pazienti che entrano nello studio clinico sono assegnati al trattamento A e B secondo la permutazione prescelta. Per il prossimo blocco di 4, un'altra combinazione di trattamento viene scelta in maniera random tra le sei permutazioni di cui sopra e i prossimi quattro pazienti sono assegnati ai trattamenti A o B secondo il modello. Questo processo continua fino alla fine dello studio.

Il vantaggio, utilizzando questo schema, è che la differenza nel numero di pazienti che ricevono A contro B non può superare 2, indipendentemente da quando termina lo studio.

## Livello di significatività

Per l’errore di I tipo, si assume un livello di significatività pari a 0.05.

## Gestione dei dati mancanti

La non compliance al trattamento assegnato per randomizzazione verrà trattata mediante un modello analitico *intent-to-treat*: il confronto tra trattamenti si basa sulla differenza tra le risposte medie tra gruppi randomizzati, ignorando il fatto che alcuni pazienti sono non compliant.

**8.6. Interim analysis**

Al fine di confermare l’eventuale sussistenza di una dimostrata superiorità del trattamento sperimentale a partire da dati intermedi o al contrato la dimostrata insussistenza di un trend di interesse, viene programmata una interim analysis a 12 mesi dall’inizio del reclutamento mediante metodi group-sequential utilizzando le soglie di arresto di O’Brian-Fleming. La dimensione campionaria del trial tiene conto del fattore di inflazione derivante dall’uso delle soglie.

# ACCESSO DIRETTO AI DOCUMENTI ORIGINALI

Lo Sperimentatore deve permettere alle Autorità Regolatorie ed al personale designato dal Comitato Etico o dallo Sperimentatore/Sponsor l’accesso diretto a tutta la documentazione originale dello studio, inclusi i moduli di Consenso Informato firmati dai soggetti inseriti nello studio e le cartelle ospedaliere e/o registri ambulatoriali. Coloro che hanno accesso diretto a tale documentazione devono prendere ogni ragionevole precauzione per mantenere riservata l’identità dei soggetti e le informazioni confidenziali di proprietà dello Sperimentatore/Sponsor, nel rispetto delle disposizioni normative applicabili.

# GESTIONE DEI DATI CLINICI

Solo lo Sperimentatore/Sponsor è proprietario di tutti i dati e dei risultati dello studio.

La gestione dei dati e la loro registrazione sarà affidata alla CRO Pharmaceutical Development and Services srl.

# PROCEDURE DI CONTROLLO E DI ASSICURAZIONE DELLA QUALITA’

## Scheda Raccolta Dati (CRF)

I dati saranno raccolti mediante scheda raccolta dati elettronica (eCRF). Verrà utilizzato per la raccolta e la gestione dei dati il programma PheedIt. Il software Pheedit è compliance alla normativa vigente (Eudralex Vol.4 GMP_Annex 11).

## Monitoraggio

La CRO, tramite personale da essa incaricato, prima dell’inizio dello studio illustrerà allo Sperimentatore, e ad eventuali altre persone dello staff coinvolte, il protocollo, la CRF, e tutti gli aspetti operativi inerenti lo studio.

Lo studio sarà monitorato dalla CRO Pharma D&S. Il monitor revisionerà i documenti originali pertinenti in accordo con le Standard Operating Procedures (SOP) e con le norme di Buona Pratica Clinica (GCP), come applicabili agli studi “no-profit”. Tra i documenti originali sono compresi i referti degli esami strumentali e di laboratorio richiesti dallo studio, ed ogni altra documentazione medica necessaria per confermare le informazioni contenute nelle schede raccolta dati.

Lo Sperimentatore dovrà essere disponibile per le visite di monitoraggio ogni qualvolta lo Sponsor lo riterrà necessario. Lo Sperimentatore e il suo staff dovranno cooperare con il Monitor durante le procedure di monitoraggio e fornire tutte le informazioni necessarie.

Tutte le attività di monitoraggio saranno riportate su appositi moduli, che saranno archiviati nel Trial Master File.

## Audit e Ispezioni

Gli sperimentatori e lo Sperimentatore/Sponsor devono permettere alle Istituzioni competenti e alle Autorità Regolatorie di effettuare gli audit e le ispezioni, come parte integrante del sistema di assicurazione della qualità.

In ogni caso sarà mantenuta la riservatezza dei dati. Tutti gli audit e le ispezioni saranno poi documentati e tenuti nel file dello studio.

# ASPETTI ETICI

## Autorizzazioni Etiche

Lo studio verrà iniziato solo dopo il parere etico rilasciato dal Comitato Etico e l’autorizzazione rilasciata dall’Autorità Competente.

Prima di poter iniziare lo studio, lo sperimentatore dovrà aver ricevuto una copia del documento di approvazione e autorizzazione.

Per ogni emendamento sostanziale protocollo sarà richiesta l’approvazione del Comitato Etico.

## Consenso Informato

Prima dell’inizio dello studio, le “Informazioni scritte per il Paziente” da fornire ai soggetti ed il modulo/i di Consenso Informato devono essere sottoposti all’esame e approvazione da parte del Comitato Etico locale, contestualmente al protocollo.

Il Consenso Informato deve essere richiesto, ottenuto e documentato dallo Sperimentatore in ottemperanza alle disposizioni normative applicabili, alla GCP ed ai principi etici che si originano dalla Dichiarazione di Helsinki.

Le garanzie relative alla protezione e la tutela dei dati personali ai sensi del Decreto legislativo 30.6.03 n. 196 (“codice sulla privacy”) saranno fornite nelle “Informazioni scritte per il Paziente” oppure in accordo a quanto previsto dalle procedure e dalla modulistica in vigore presso i singoli centri sperimentali.

Lo Sperimentatore è responsabile dell’ottenimento del consenso informato da parte dei pazienti o dei propri rappresentanti legali prima dell’arruolamento nello studio; pertanto, prima delle procedure di screening, egli informerà, con un linguaggio comprensibile, i pazienti e/o i propri rappresentanti legali circa il tipo di studio, lo scopo, i rischi e i benefici da esso derivanti e risponderà a tutte le domande riguardanti lo studio. Ottenute le informazioni necessarie, il modulo del consenso informato sarà firmato e datato dal paziente e/o rappresentante legale e dallo Sperimentatore. Di questi una copia sarà consegnata al soggetto o rappresentante legale, mentre gli originali saranno inseriti nella documentazione dello studio e saranno conservati dallo Sperimentatore per tempi prescritti.

## Good Clinical Pratice

Tutte le parti coinvolte nello studio concordano e verificano che questa sperimentazione sia condotta in conformità ai principi etici, che traggono la loro origine dalla Dichiarazione di Helsinki (Appendice I), ai principi della Buona Pratica Clinica (DM n. 162 del 15/07/97, come applicabile agli studi “no-profit”) e alle disposizioni normative applicabili.

Lo studio verrà condotto tenendo conto dei requisiti regolatori e degli adempimenti di legge.

# PROCEDURE AMMINISTRATIVE

## Archiviazione

Lo Sperimentatore/Sponsor deve provvedere alla conservazione dei documenti essenziali dello studio come specificato dai principi delle GCP e in accordo alle disposizioni normative applicabili. Lo Sperimentatore/Sponsor deve adottare le misure necessarie per impedire la distruzione accidentale o prematura.

Lo Sperimentatore/Istituzione deve conservare i documenti essenziali per almeno 7 anni dal termine dello studio in conformita' al D.Lvo 200/2007, art. 18, comma 1. Tuttavia, questi documenti devono essere conservati per periodi più lunghi se richiesto dalle disposizioni normative applicabili.

## Confidenzialità delle informazioni

I dati raccolti nel corso dello studio saranno trattati nel rispetto della legge relativa alla Tutela dei dati personali ( Decreto legislativo 30.6.03 n. 196; “codice sulla privacy”) e successive modifiche ed integrazioni.

I dati indicati saranno raccolti dal medico dello studio, in forma anonima, esclusivamente in funzione della realizzazione dello studio e della partecipazione ad esso. Nella Scheda Raccolta Dati (CRF) deve comparire solamente il numero del paziente e le sue iniziali e se il nome del paziente comparisse su alcun documento (ad es. esami di laboratorio o altri referti) il nome deve essere cancellato prima che la copia del documento venga consegnata allo Sperimentatore/Sponsor.

Lo sperimentatore deve conservare una lista dei pazienti in modo che sia possibile la loro identificazione.

## Copertura assicurativa

Lo studio è coperto da specifica polizza assicurativa, della compagni assicurativa HDI-Gerling.

# RESPONSABILITA’ DELLO SPERIMENTATORE

Lo Sperimentatore è tenuto a condurre lo studio in conformità al protocollo di studio, in accordo con i principi della Buona Pratica Clinica (DM n. 162 del 15/07/97 e successive integrazioni), con i principi della Dichiarazione di Helsinki (Appendice I) e in conformità alla normativa applicabile .

Lo Sperimentatore deve assicurare che tutto il personale coinvolto nello studio sia adeguatamente informato sul protocollo, sul trattamento in studio e sui controlli da eseguirsi. Lo Sperimentatore manterrà una lista del personale qualificato ai quali sono stati demandati alcune mansioni correlate allo studio.

Lo Sperimentatore è consapevole di essere responsabile di tutte le azioni da lui delegate agli altri membri del Suo staff designati alla conduzione dello studio.

# PUBBLICAZIONE DEI DATI

Lo studio è stato inserito nella banca dati EudraCT, come richiesto dalla normativa vigente.

I risultati dello studio saranno pubblicati su riviste internazionali provviste di impact factor.

Il nome e l’ordine degli autori saranno concordati tra lo Sponsor e gli Sperimentatori.

# BIBLIOGRAFIA

1. *McInnes IB, Schett G.* [*The pathogenesis of rheumatoid arthritis.*](http://www.ncbi.nlm.nih.gov/pubmed/22150039) *N Engl J Med. 2011 Dec 8;365(23):2205-19.*
2. *Avina-Zubieta JA, Thomas J, Sadatsafavi M, Lehman AJ, Lacaille D.* [*Risk of incident cardiovascular events in patients with rheumatoid arthritis: a meta-analysis of observational studies.*](http://www.ncbi.nlm.nih.gov/pubmed/22425941) *Ann Rheum Dis. 2012 Sep;71(9):1524-9.*
3. *Wasko MC, Kay J, Hsia EC, Rahman MU.* [*Diabetes mellitus and insulin resistance in patients with rheumatoid arthritis: risk reduction in a chronic inflammatory disease.*](http://www.ncbi.nlm.nih.gov/pubmed/21452264) *Arthritis Care Res (Hoboken). 2011 Apr;63(4):512-21)*
4. *Gonzalez-Gay MA, De Matias JM, Gonzalez-Juanatey C, Garcia-Porrua C, Sanchez-Andrade A, Martin J, Llorca J.* [*Anti-tumor necrosis factor-alpha blockade improves insulin resistance in patients with rheumatoid arthritis.*](http://www.ncbi.nlm.nih.gov/pubmed/16539824) *Clin Exp Rheumatol. 2006 Jan-Feb;24(1):83-6*
5. *Yudkin JS, Kumari M, Humphries SE, Mohamed-Ali V.* [*Inflammation, obesity, stress and coronary heart disease: is interleukin-6 the link?*](http://www.ncbi.nlm.nih.gov/pubmed/10657556) *Atherosclerosis 2000 Feb;148(2):209-14.*
6. *Larsen CM, Faulenbach M, Vaag A, Vølund A, Ehses JA, Seifert B, Mandrup-Poulsen T, Donath MY. Interleukin-1-receptor antagonist in type 2 diabetes mellitus. N Engl J Med. 2007 Apr 12;356(15):1517-26*
7. *A. Mosca et al, Recommendations for the implementation of the International standardization of glycated hemoglobin measurementin Italy G It Diabetol Metab 2009;29:184-188*

## FLOW CHART E TABELLA DEI TEMPI

|  | **Screening** | **Trattamento** | | | | |
| --- | --- | --- | --- | --- | --- | --- |
| **Visita** | 0 | 1 | 2 | 3 | 4 | 5 |
| **Mese** | selezione | 0 | 3 | 6 | 12 | 24 |
| **Finestra** |  | 7 gg | ±7 gg | ± 7 gg | ± 7 gg | ± 7 gg |
| **Consenso Informato** | x |  |  |  |  |  |
| **Dati Demografici** | x |  |  |  |  |  |
| **Anamnesi** | x |  |  |  |  |  |
| **Segni Vitali** | x |  | x | x | x | x |
| **Esame obiettivo** | x |  | x | x | x | x |
| **BMI** | x |  | x | x | x | x |
| **Valutazione clinica AR** | x |  | x | x | x | x |
| **Valutazione Biochimica AR** | x |  | x | x | x | x |
| **Valutazione HbA_1c_** | x |  | x | x | x | x |
| **Glicemia –Trigliceridi - Colesterolo Tot -HDL - LDL** | x |  | **x** | **x** | **x** | **x** |
| **Raggi X del Torace** | x |  |  | x ^*^ |  |  |
| **Profilassi tubercolare^**^** |  | x |  |  |  |  |
| **PPD e/o QFT** | x |  |  |  |  |  |
| **ECG a 12 derivazioni** | x |  |  |  |  |  |
| **Segni e Sintomi Cardiopatia Ischemica** | x |  |  |  |  |  |
| **Esame emocromocitometrico** | x |  | x | x | x | x |
| **Esami chimica clinica** | x |  | x | x | x | x |
| **Esame urine e microalbuminuria** | x |  | x | x | x | x |
| **Test di gravidanza** | x |  |  |  |  |  |
| **Registrazione farmaci concomitanti** | x |  | x | x | x | x |
| **Registrazione patologie concomitanti** |  |  | x | x | x | x |
| **Criteri inclusione/esclusione** |  | x | x | x | x | x |
| **Randomizzazione** |  | x |  |  |  |  |
| **Parametri ecocardiografici** |  | x |  | x | x | x |
| **Retinopatia diabetica** |  | x | x | x | x | x |
| **Consegna Diario paziente** |  | x | x | x | x |  |
| **Ritiro del Diario paziente** |  |  | x | x | x | x |
| **Avviso arruolamento** |  | x |  |  |  |  |
| **Eventi Avversi** |  |  | x | x | x | x |
| **Compliance** |  |  | x | x | x | x |
| **Procedure di chiusura** |  |  |  |  |  | x |

* L’esame si ripete alla visita a sei mesi nel caso di pazienti che hanno ricevuto la profilassi TB a causa di test positivi a precedente infezione tubercolotica

** Solo per pazienti senza malattia in atto ma risultati positivi all’esame radiografico del torace e/o al PPD e/o QFT

I pazienti verranno trattati secondo il seguente schema:

Mese 0

Mese 3

Mese 6

Mese 12

Mese 24

Visita 0

Visita 1

Visita 2

Visita 3

Visita 4

Visita 5

10 giorni

Gruppo Anakinra

Gruppo Inibitori TNF-α

Screening

Consenso Informato

Randomizzazione

Chiusura Studio Clinico

ALLEGATO 1 – DICHIARAZIONE DI HELSINKY

ALLEGATO 2 – RIASSUNTO DELLE CARATTERISTICHE DEL PRODOTTO DI KINERET®

ALLEGATO 3 - QUESTIONARIO HAQ E I CRITERI PER L’ASSEGNAZIONE DEL PUNTEGGIO
