# Supplementary material for: Anti-interleukin-1 treatment in patients with rheumatoid arthritis and type 2 diabetes (TRACK): A multicentre, open-label, randomised controlled trial
Source: PLoS Med. 2019 Sep 12;16(9):e1002901. doi: 10.1371/journal.pmed.1002901 (PMC6742232; doi:10.1371/journal.pmed.1002901)
Supplement: S1 Table — HbA1c, glycated haemoglobin; TNFi, tumour necrosis factor inhibitor. (DOCX) [file pmed.1002901.s005.docx]

**S1 Table. Mean values of HbA1c in anakinra- and TNFi-treated participants.**

| **Participants, n** | **HbA1c% (mmol/mol)**  **Mean ± SD** | **Anakinra vs TNFi**  **P values** |
| --- | --- | --- |
| Anakinra (Time 0),  n: 22 | 7.73 (61) ± 0.67 | / |
| TNFi (Time 0),  n: 17 | 7.83 (62) ± 0.76 |  |
|  |  |  |
| Anakinra (3 months),  n: 18 | 6.95 (51) ± 0.61 | **0.0038** |
| TNFi (3 months),  n: 16 | 7.63 (60) ± 0.68 |  |
|  |  |  |
| Anakinra (6 months),  n: 17 | 6.70 (50) ± 0.67 | **0.0004** |
| TNFi (6 months),  n: 15 | 7.64 (60) ± 0.65 |  |
| HbA1c=glycated haemoglobin. TNFi=tumour necrosis factor inhibitor. Statistical significance was expressed by a p value <0.05. Bolded values indicate statistically significant results. | | |
