# Supplementary material for: Anti-interleukin-1 treatment in patients with rheumatoid arthritis and type 2 diabetes (TRACK): A multicentre, open-label, randomised controlled trial
Source: PLoS Med. 2019 Sep 12;16(9):e1002901. doi: 10.1371/journal.pmed.1002901 (PMC6742232; doi:10.1371/journal.pmed.1002901)
Supplement: S2 Table — FPG, fasting plasma glucose; TNFi, tumour necrosis factor inhibitor. (DOCX) [file pmed.1002901.s006.docx]

**S2 Table. Mean values of FPG in anakinra- and TNFi-treated participants.**

| **Participants, n** | **FPG mg/dL**  **Mean ± SD** | **Anakinra vs TNFi**  **P values** |
| --- | --- | --- |
| Anakinra (Time 0),  n: 22 | 139.05 ± 50.09 | / |
| TNFi (Time 0),  n: 17 | 139.25 ± 29.55 |  |
|  |  |  |
| Anakinra (3 months),  n: 18 | 109.78 ± 30.58 | **0.027** |
| TNFi (3 months),  n: 16 | 133.06 ± 27.72 |  |
|  |  |  |
| Anakinra (6 months),  n: 17 | 100.81 ± 11.11 | **0.001** |
| TNFi (6 months),  n: 15 | 140.93 ± 39.45 |  |
| FPG=fasting plasma glucose. TNFi=tumour necrosis factor inhibitor. Statistical significance was expressed by a p value <0.05. Bolded values indicate statistically significant results. | | |
