# Supplementary material for: Anti-interleukin-1 treatment in patients with rheumatoid arthritis and type 2 diabetes (TRACK): A multicentre, open-label, randomised controlled trial
Source: PLoS Med. 2019 Sep 12;16(9):e1002901. doi: 10.1371/journal.pmed.1002901 (PMC6742232; doi:10.1371/journal.pmed.1002901)
Supplement: S3 Table — TNFi, tumour necrosis factor inhibitor. (DOCX) [file pmed.1002901.s007.docx]

**S3 table. Mean values of albuminuria in anakinra- and TNFi-treated participants.**

| **Participants, n** | **Albuminuria mg/L**  **Mean ± SD** | **Anakinra vs TNFi**  **P values** |
| --- | --- | --- |
|  |  |  |
| Anakinra (Time 0),  n: 22 | 11.93 ± 12.14 | / |
| TNFi (Time 0),  n: 17 | 5.98 ± 8.15 |  |
|  |  |  |
| Anakinra (3 months),  n: 18 | 7.24 ± 11.48 | 0.90 |
| TNFi (3 months),  n: 15 | 6.69 ± 13.17 |  |
|  |  |  |
| Anakinra (6 months),  n: 15 | 5.14 ± 8.47 | 0.58 |
| TNFi (6 months),  n: 15 | 7.33 ± 12.81 |  |
|  |  |  |
| Abbreviations: TNFi: TNF inhibitor. | | |
