# Supplementary material for: Anti-interleukin-1 treatment in patients with rheumatoid arthritis and type 2 diabetes (TRACK): A multicentre, open-label, randomised controlled trial
Source: PLoS Med. 2019 Sep 12;16(9):e1002901. doi: 10.1371/journal.pmed.1002901 (PMC6742232; doi:10.1371/journal.pmed.1002901)
Supplement: S4 Table — BMI, body mass index; TNFi, tumour necrosis factor inhibitor. (DOCX) [file pmed.1002901.s008.docx]

**S4 Table. Mean values of BMI in anakinra- and TNFi-treated participants.**

| **Patients, n** | **BMI**  **Mean ± SD** | **Anakinra vs TNFi**  **P values** |
| --- | --- | --- |
|  |  |  |
| Anakinra (Time 0),  n: 22 | 27.59 ± 4.48 | / |
| TNFi (Time 0),  n: 17 | 28.38 ± 3.47 |  |
|  |  |  |
| Anakinra (3 months),  n: 20 | 27.95 ± 4.64 | 0.76 |
| TNFi (3 months),  n: 15 | 28.37± 3.50 |  |
|  |  |  |
| Anakinra (3 months),  n: 16 | 27.89 ± 4.12 | 0.62 |
| TNFi (3 months),  n: 15 | 28.58 ± 3.64 |  |
|  |  |  |
| Abbreviations: BMI: body mass index; TNFi: TNF inhibitor. | | |
