# Supplementary material for: Anti-interleukin-1 treatment in patients with rheumatoid arthritis and type 2 diabetes (TRACK): A multicentre, open-label, randomised controlled trial
Source: PLoS Med. 2019 Sep 12;16(9):e1002901. doi: 10.1371/journal.pmed.1002901 (PMC6742232; doi:10.1371/journal.pmed.1002901)
Supplement: S5 Table — TNFi, tumour necrosis factor inhibitor. (DOCX) [file pmed.1002901.s009.docx]

**S5 Table. Diabetic Retinopathy in anakinra- and TNFi-treated participants.**

|  | **“No” Diabetic Retinopathy** | **“Yes” Diabetic Retinopathy** | **Total** |
| --- | --- | --- | --- |
| **Anakinra** |  |  |  |
| Frequency | 16 | 2 | 18 |
| row percentage | 88.89% | 11.11% | 100.00% |
| column percentage | 55.17% | 50.00% | 54.55% |
| **TNFi** |  |  |  |
| Frequency | 13 | 2 | 15 |
| row percentage | 86.67% | 13.33% | 100.00% |
| column percentage | 44.83% | 50.00% | 45.45% |
| **Total** |  |  |  |
| Frequency | 29 | 4 | 33 |
| row percentage | 87.88% | 12.12% | 100.00% |
| column percentage | 100.00% | 100.00% | 100.00% |
| Fisher's exact test = 1.000 | | | |
| TNFi=tumour necrosis factor inhibitor. Statistical significance was expressed by a p value <0.05. | | | |
