# Supplementary material for: Anti-interleukin-1 treatment in patients with rheumatoid arthritis and type 2 diabetes (TRACK): A multicentre, open-label, randomised controlled trial
Source: PLoS Med. 2019 Sep 12;16(9):e1002901. doi: 10.1371/journal.pmed.1002901 (PMC6742232; doi:10.1371/journal.pmed.1002901)
Supplement: S6 Table — DAS28, Disease Activity Score-28; TNFi, tumour necrosis factor inhibitor. (DOCX) [file pmed.1002901.s010.docx]

**S6 Table. Mean values of DAS28 in anakinra- and TNFi-treated participants.**

| **Participants, n** | **DAS28**  **Mean ± SD** | **Anakinra vs TNFi**  **P values** |
| --- | --- | --- |
| Anakinra (Time 0),  n: 22 | 5.42 ± 1.18 | / |
| TNFi (Time 0),  n: 17 | 5.70 ± 0.80 |  |
|  |  |  |
| Anakinra (3 months),  n: 19 | 2.95 ± 1.58 | **0.039** |
| TNFi (3 months),  n: 16 | 3.94 ± 1.01 |  |
|  |  |  |
| Anakinra (6 months),  n: 17 | 2.70 ± 1.16 | 0.08 |
| TNFi (6 months),  n: 15 | 3.58 ± 1.45 |  |
| DAS28=disease activity score in 28 joints. TNFi=tumour necrosis factor inhibitor. Statistical significance was expressed by a p value <0.05. Bolded values indicate statistically significant results. | | |
