# Supplementary material for: Anti-interleukin-1 treatment in patients with rheumatoid arthritis and type 2 diabetes (TRACK): A multicentre, open-label, randomised controlled trial
Source: PLoS Med. 2019 Sep 12;16(9):e1002901. doi: 10.1371/journal.pmed.1002901 (PMC6742232; doi:10.1371/journal.pmed.1002901)
Supplement: S7 Table — EULAR, European League Against Rheumatism. (DOCX) [file pmed.1002901.s011.docx]

**S7 Table. Percentage of participants reaching good EULAR clinical response and remission.**

|  | **Anakinra** | **TNFi** | **Total** |
| --- | --- | --- | --- |
| **“No” Good EULAR Response** |  |  |  |
| Frequency | 1 | 6 | 7 |
| row percentage | 14.29% | 85.71% | 100.00% |
| column percentage | 5.00% | 37.50% | 19.44% |
| **“Yes” Good EULAR Response** |  |  |  |
| Frequency | 19 | 10 | 29 |
| row percentage | 65.52% | 34.48 | 100.00% |
| column percentage | 95.00% | 62.50% | 80.56% |
| **Total** |  |  |  |
| Frequency | 20 | 16 | 36 |
| row percentage | 55.56% | 44.44% | 100.00% |
| column percentage | 100.00% | 100.00% | 100.00% |
| Fisher's exact test = **0.030** | | | |
|  | **Anakinra** | **TNFi** | **Total** |
| **“No” Remission** |  |  |  |
| Frequency | 10 | 12 | 22 |
| row percentage | 45.45% | 54.55% | 100.00% |
| column percentage | 50.00% | 75.00% | 61.11% |
| **Yes” Remission** |  |  |  |
| Frequency | 10 | 4 | 14 |
| row percentage | 71.43% | 28.57% | 100.00% |
| column percentage | 50.00% | 25.00% | 38.89% |
| **Total** |  |  |  |
| Frequency | 20 | 16 | 36 |
| row percentage | 55.56% | 44.44% | 100.00% |
| column percentage | 100.00% | 100.00% | 100.00% |
| Fisher's exact test = 0.176 | | | |
| TNFi=tumour necrosis factor inhibitor. Statistical significance was expressed by a p value <0.05. Bolded values indicate statistically significant results. | | | |
