# Supplementary material for: Anti-interleukin-1 treatment in patients with rheumatoid arthritis and type 2 diabetes (TRACK): A multicentre, open-label, randomised controlled trial
Source: PLoS Med. 2019 Sep 12;16(9):e1002901. doi: 10.1371/journal.pmed.1002901 (PMC6742232; doi:10.1371/journal.pmed.1002901)
Supplement: S8 Table — SDAI, simplified disease activity index; TNFi, tumour necrosis factor inhibitor. (DOCX) [file pmed.1002901.s012.docx]

**S8 Table. Mean values of SDAI in anakinra- and TNFi-treated participants.**

| **Participants, n** | **SDAI**  **Mean ± SD** | **Anakinra vs TNFi**  **P values** |
| --- | --- | --- |
|  |  |  |
| Anakinra (Time 0),  n: 22 | 34.98 ± 25.18 | / |
| TNFi (Time 0),  n: 17 | 35.86 ± 3.47 |  |
|  |  |  |
| Anakinra (3 months),  n: 19 | 17.70 ± 10.53 | 0.90 |
| TNFi (3 months),  n: 16 | 18.69 ± 29.55 |  |
|  |  |  |
| Anakinra (6 months),  n: 16 | 7.89 ± 9.23 | **0.048** |
| TNFi (6 months),  n: 15 | 14.93 ± 9.92 |  |
|  |  |  |
| Abbreviations: SDAI: simplified disease activity score; TNFi: TNF inhibitor. Statistical significance was expressed by a p value <0.05. Bolded values indicate statistically significant results. | | |
