# Supplementary material for: Anti-interleukin-1 treatment in patients with rheumatoid arthritis and type 2 diabetes (TRACK): A multicentre, open-label, randomised controlled trial
Source: PLoS Med. 2019 Sep 12;16(9):e1002901. doi: 10.1371/journal.pmed.1002901 (PMC6742232; doi:10.1371/journal.pmed.1002901)
Supplement: S9 Table — PGA, physician global assessment; TNFi, tumour necrosis factor inhibitor. (DOCX) [file pmed.1002901.s013.docx]

**S9 Table. Mean values of PGA in anakinra- and TNFi-treated participants.**

| **Participants, n** | **PGA**  **Mean ± SD** | **ANA vs TNFi**  **P values** |
| --- | --- | --- |
|  |  |  |
| Anakinra (Time 0),  n: 22 | 61.90 ± 19.17 | / |
| TNFi (Time 0),  n: 17 | 62.00 ± 17.81 |  |
|  |  |  |
| Anakinra (3 months),  n: 19 | 22.21 ± 21.86 | 0.27 |
| TNFi (3 months),  n: 16 | 30.13 ± 19.66 |  |
|  |  |  |
| Anakinra (6 months),  n: 16 | 18.53 ± 23.53 | 0.45 |
| TNFi (6 months),  n: 15 | 25.97 ± 19.99 |  |
|  |  |  |
| Abbreviations: PGA: physician global assessment; TNFi: TNF inhibitor.  Statistical significance was expressed by a p value <0.05. | | |
