# Supplementary material for: Anti-interleukin-1 treatment in patients with rheumatoid arthritis and type 2 diabetes (TRACK): A multicentre, open-label, randomised controlled trial
Source: PLoS Med. 2019 Sep 12;16(9):e1002901. doi: 10.1371/journal.pmed.1002901 (PMC6742232; doi:10.1371/journal.pmed.1002901)
Supplement: S10 Table — TNFi, tumour necrosis factor inhibitor; VAS, visual analogue scale. (DOCX) [file pmed.1002901.s014.docx]

**S10 Table. Mean values of VAS of pain in anakinra- and TNFi-treated participants.**

| **Participants, n** | **VAS PAIN**  **Mean ± SD** | **Anakinra vs TNFi**  **P values** |
| --- | --- | --- |
|  |  |  |
| Anakinra (Time 0),  n: 22 | 66.86 ± 29.46 | / |
| TNFi (Time 0),  n: 17 | 68.94 ± 22.86 |  |
|  |  |  |
| Anakinra (3 months),  n: 19 | 35.37 ± 23.74 | 0.28 |
| TNFi (3 months),  n: 16 | 44.00 ± 21.98 |  |
|  |  |  |
| Anakinra (6 months),  n: 16 | 27.47 ± 21.67 | 0.24 |
| TNFi (6 months),  n: 15 | 26.46 ± 28.38 |  |
|  |  |  |
| Abbreviations: VAS: visual analogue scale; TNFi: TNF inhibitor.  Statistical significance was expressed by a p value <0.05. | | |
